# Life at the Top: Extensive Green Roof Plant Species and Their Traits for Urban Use

**DOI:** 10.3390/plants14050735

**Published:** 2025-02-27

**Authors:** Cristina C. Todeschini, Arthur G. Fett-Neto

**Affiliations:** Plant Physiology Laboratory, Department of Botany and Center for Biotechnology, Federal University of Rio Grande do Sul (UFRGS), Av. Bento Gonçalves 9500, bl IV, pr. 43423, rm 220, Porto Alegre 91501-970, RS, Brazil; cristinatodeschini@hotmail.com

**Keywords:** green roofs, review, rooftop plants, plant species, climate

## Abstract

Green roofs are becoming common in cities around the world. Rooftop vegetation faces severe and unique conditions ranging from shallow substrates, high irradiance, water limitation, flooding, extreme temperatures, and strong winds. To benefit the most from this technology for improving urban climate, ecological interactions, and human life quality, it is important to have a toolbox of candidate plant species adapted to the rooftop environment. Herein, an effort was made to provide an overview of the global scenario of green roof plants. A quantitative and qualitative review examining 439 articles published within an approximate 14-year time window (2009 to mid-2023) was conducted to categorize plants amenable to use in green roof conditions, and to identify and discuss their key morphophysiological phenotypic features. This dataset allowed the establishment of archetypal green roof plants for use in different climates. Similar traits were recorded between temperate and continental climate species on one side and between tropical and dry climate plants on the other. The identified set of species and their archetypes within each major climate zone can be useful for readily adopting and expanding new green roofs and as a guideline for incorporating new taxa into this urban environmental technology.

## 1. Introduction and Background

Urbanization is dominant in several parts of the world, and the planning of sustainable cities has become essential. Roof surfaces represent a large part of the urban setting cover, with potential to significantly improve the sustainable performances of buildings [1]. The establishment of vegetation on roofs, known as green roofs, is increasingly used as it provides advantages in terms of physical, ecological, and esthetic features. Green roofs are a tool to recover lost green spaces that can help mitigate rainwater runoff by reducing the volume of flowing water in urban waterways and limiting floods [2]. In addition, green roofs provide ecosystem services in urban areas, including better regulation of building temperatures (saving energy, because of their thermal insulating effect), but also reducing the urban effects of heat islands and relieving the negative effects of pollution [3]. Green surfaces can support microecosystems providing shelter and food to native species of birds, butterflies, bees, and beetles, among others, including endangered/rare organisms [4]. These roofs also help improve air quality by sequestering carbon, releasing oxygen through photosynthesis, and increasing humidity by liberating water vapor during transpiration. Moreover, most people long for greater contact with nature, which often helps reducing psychological stress levels [5]. In an era of climate fluctuation, increased rainfall intensity, and extended periods of drought, the capacity of green roofs to act as a buffer for local climate changes will make them even more significant [6]. Finally, all these features combined contribute positively to the economic value of buildings.

Green roofs are classified based on substrate thickness, vegetation layer, need for irrigation, and maintenance demands. Although there are several classification systems, the most frequently used defines three basic types: extensive, semi-intensive, and intensive [7]. Extensive roofs are the shallower of them all (about 7 to 10 cm), have a coarse substrate media, have no separate drainage layer or no requirement for irrigation, and have a weight tolerance of 60 to 150 kg.m^−2^, being suitable for areas with reasonable precipitation. Extensive roofs are fit for succulents and ornamental plants of relatively low stature. Semi-intensive roofs range from about 15 to 31 cm in thickness, bear a separate drainage layer, have multi-course substrate media and a weight tolerance of circa 150 kg.m^−2^, and support a wider range of plant types, including ornamental, meadow species, turf grass, and some woody perennials. If meadow grass species are used, an irrigation layer is often required. Both extensive and semi-intensive roof types are relatively affordable and rather commonly employed. Intensive roofs have deeper substrates (above 31 cm), need a separate drainage system and irrigation layer, require a media type having an intensive growth layer, and have a weight tolerance ranging from 200 to 500 kg.m^−2^, being fit for ground-level plant species. As a result of the complex structure and maintenance needs, this type of green roof is less common and more costly. In this review, the focus is on species used in extensive green roofs.

The growth of plants on green roofs occurs under unique conditions. Plants on rooftops are subjected to heavy incidence of heat, irradiance, drought, salinity, wind, and substrate restriction, demanding important adaptation features [8]. The frequent recurrence of periods of stressful conditions leading to growth limitation is a common feature in rooftop environments. Plants on rooftops are often restricted by substrate availability, especially as they age, and root systems become larger. Nutrient availability can also be limiting since biomass cycling and decomposition is slower compared to ground-level environments. This condition may be partly mitigated by substrate inoculation with microorganisms such as nitrogen fixing bacteria and mycorrhiza. In fact, beneficial microbes can make plants more resilient to a wide range of stresses, both biotic and abiotic, a strategy that can be useful in green roofs [9]. Water limitation is frequently associated with heat or cold (physiological drought), and in the former case is sometimes associated with salinization of the substrate. On the other hand, temporary root hypoxia due to substrate flooding during intense rain may also occur. Excess irradiance and intense winds contribute to the stressful scenario, often leading to photo-oxidative inhibition and drying.

Individual stress factors rarely occur in isolation; various stress factors are often experienced simultaneously or in a sequential manner, requiring different defense mechanisms and timing of responses by plant metabolism [10]. Given these environmental features of green roofs, the objectives guiding plant selection include tolerance to water stress (drought and flooding), high irradiance, temperature stress (heat and cold), mineral nutrient limitation, salinity, and adequate plant architecture (i.e., shallow branched roots and compact sturdy shoots). Underlying these characteristics, an able balance between photosynthesis and transpiration under a variety of conditions will be essential whichever the plant photosynthetic mode (C3, C4, CAM, or hybrids of these that can shift, such as C3-CAM and C3-C4). Desiccation-tolerant species, such as *Selaginella* spp. [11], are an interesting option of resilient plants; however, in their dehydrated state, these individuals contribute little to the benefits of green roof vegetation.

Green roofs are indeed living systems, and the selection criteria for the species have a significant role in their effective function. Since the commercial introduction of extensive green roofs, the succulent species of Crassulaceae, particularly the genera *Sedum* and *Phedimus* (formerly classified within the *Sedum* genus) have dominated rooftop covers due to their successful proliferation on European green roofs [12]. They have shallow root systems and facultative crassulacean acid metabolism (CAM), and hence efficient water use and tolerance to extreme conditions of drought and thin substrates, characteristic of rooftops [13]. They are also the main type of plants used for green roofs due to a low requirement of irrigation and adaptability to sunny environments.

CAM metabolism confers high water use efficiency and low photorespiration, using a temporal separation between initial carbon fixation and release of CO_2_ around ribulose 1,5-bisphosphate carboxylase oxygenase (RUBISCO). Stomata in these plants open at night, and CO_2_ (in fact bicarbonate, thereby eliminating any competition with O_2_, unlike the case of RUBISCO) is fixed by the high affinity enzyme PEP (phosphoenolpyruvate) carboxylase as 4C organic acids which are stored in the vacuole. During daytime, when stomata are closed, these acids are decarboxylated. This constitutes an effective CO_2_ concentration mechanism to increase carboxylase and minimize oxidase functions of RUBISCO, as well as reduce transpiration [14]. *Sedum* species can also facilitate the performance of neighboring plants through ‘nursery effects’ by reducing soil temperature during dry weather conditions and improving moisture availability, thereby decreasing abiotic stress for different life forms [15].

Other types of plants widely used in green roofs are C4 (mostly grasses). These species have a low irrigation requirement due to spatial separation-based CO_2_ concentration mechanisms, thereby minimizing photorespiration and transpiratory losses [14]. Briefly, CO_2_ is first fixed by PEP carboxylase as 4C organic acids in the cytoplasm of mesophyll cells. These organic acids are transported to chloroplasts of the vascular bundle sheath cells (Krantz anatomy) and decarboxylated near RUBISCO. This feature of roof plants becomes highly valuable in places where water availability is limited [16].

The suitability of various plant taxa to green roofs has been investigated for approximately two decades. The use of different life forms has been shown to provide better ecosystem functioning and resistance to environmental stress, due to niche complementarity and facilitation [17]. Furthermore, diverse green roofs have higher survival probability of its components and are more esthetically pleasing even under dry conditions [18,19]. In any case, there is a benefit of using native plants to which local fauna is better adapted. However, quite often the green roof vegetation is composed of non-native and low-diversity species that are favored by ease of availability and known resilience.

During the last decade or so, research and application of green roofs have shown a remarkable rise in several countries. However, knowhow and proper incentives are, to some extent, still lacking in a large part of the world [4,20,21]. This is, for instance, the case of various countries with tropical and subtropical climates. Given the harsh and unique environmental conditions on rooftops, particularly during summer, it is important to have a better knowledge of plant species that can survive and thrive in such challenging areas [13,22].

There are five distinct climate groups identified by the Köppen classification system [23]: tropical, dry, temperate (mild), continental, and polar. The last one will not be considered in the present review due to its scarcely populated areas and limited vegetation. Climate types are further subdivided into several climate groups. The tropical climate is found in intertropical regions, which are defined by year-round temperatures above 18 °C, no winter season, and an annual precipitation greater than the annual potential evapotranspiration. In tropical climates, the difference in daily temperatures between the warmest and coldest months of the year is more than the difference in average yearly temperature. Areas that fall under the category of dry climates have little precipitation. In this environment, there are significant daily and seasonal changes in temperature. Dry climates cover the warmest zones of the world. Temperate zones are those that have moderate and continental climates. There are separate cold seasons in each type of climate. Latitude and a region’s location on the continent are the main determinants of climate in certain parts of the world. Continental areas experience longer-lasting snowfall, colder winters, and shorter growing seasons. These areas serve as a transition between temperate and polar climates. Seasonal variations are very noticeable in continental regions.

The present review compiles and critically examines data of an approximate 14-year time window (2009 to mid-2023) on green roof vegetation species composition. The main goal is to provide an overview of plant traits (morphological and physiological) that are crucial for survival in this unique environment (i.e., establish a set of features of ‘archetypal’ green roof plants) for each one of the main climates of the Köppen system [23], i.e., continental, dry, temperate, and tropical. It is hoped that the database generated may facilitate expansion and implantation of green roofs and the popularization of this important environmental technology in several types of constructions and climate zones.

## 2. Literature Survey and Data Delimitation

### 2.1. Species List

**Figure 1:**
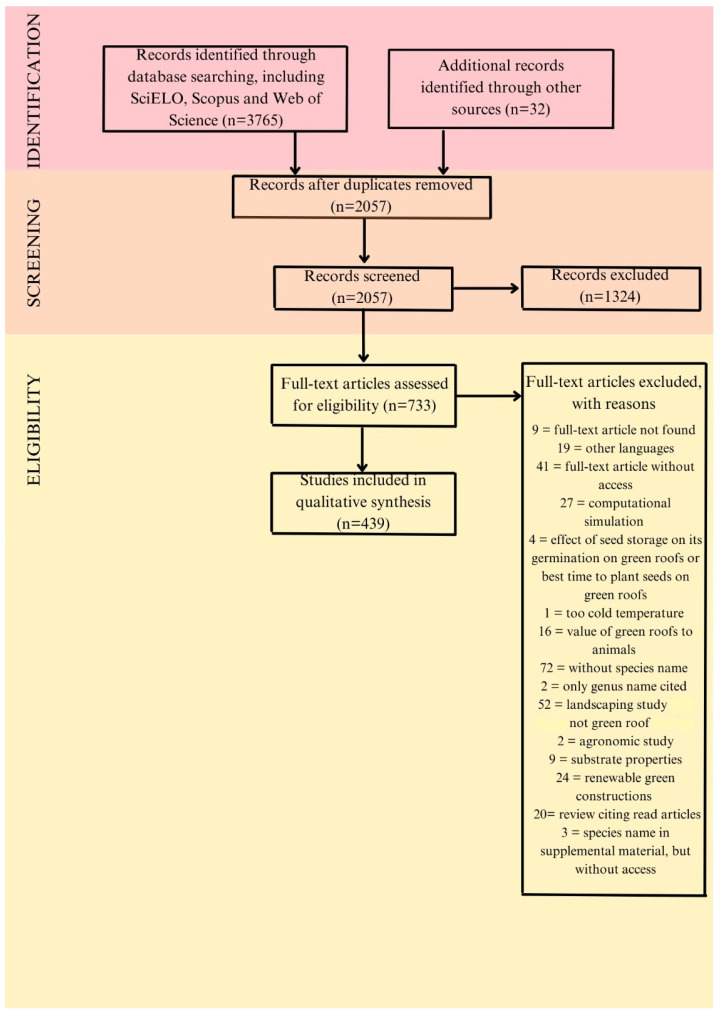
Outline of review procedure steps and results based on Prisma Flow 2009 [24].

To survey a broad set of studies, the first step entailed a thorough bibliographic search consulting different web archives and specific scientific literature following a standardized meta-analysis (Figure 1). Electronic databases (SciELO, Scopus, and Web of Science) were consulted, setting a time window from 2009 to July 2023, using the following keywords: (*techo verde* OR *telhado verde* OR *green roof* OR *rooftop* OR *roof top* OR roof garde*) AND (plant* OR *species*). Bibliographic items included articles, reviews, corrections, books, and chapters. All the data were downloaded and imported into the reference manager Mendeley (Elsevier), and duplicated records were removed. The remaining records were analyzed and the number of species that were highlighted as appropriate or not for the use in green roofs were identified, as well as the number of times a species was noted in the database (frequency of use in extensive green roof). The species list included a set of green roof plants commonly used or spontaneously occurring on extensive green roofs. Plants reported in these papers were organized in a floristic database, and their names were standardized to the most recent nomenclature using the Plant List (updated access on 12 September 2023) (SM1). To avoid repeating new scientific names and synonyms in the checklist, the taxonomic resolution of the source consulted was followed.

### 2.2. Trait Selection Procedure

Since actual species selection for extensive green roofs depends on both functional and morphological traits, the two types of features were considered. Data on plant traits easily available in the literature were used. This facilitates the use of these common traits in other studies and introduces a certain degree of independence to the analyses (i.e., parameters not necessarily restricted to specific experimental studies). Relevant traits were identified through an extensive literature search [25,26] (Table 1). Drought adaptation, morpho-physiological features, and self-regulation (regeneration strategy, presence of seed bank, plant longevity) were considered key factors for survival and overall success on extensive green roofs, which also often holds true for arid environments [26]. For simplicity, all features were referred to as plant traits and these were assembled for the 30 most frequent and unique plant species used in green roofs of the main climate types, according to the Köppen Classification System [23]. The choice of 30 species was made to provide a manageable and statistically sound dataset. All species for which no information was available were omitted as they did not contribute to the analysis. Also, some traits like a deep rooting system may be considered an adaptation to drought, but since substrate depth on extensive green roofs normally does not exceed 20 cm, all species having deep roots were removed. Other exclusion criteria were plant height exceeding 1 m, drought sensitivity, low tolerance to stresses in general, and plant inadequacy for rooftops established by the study. Thus, species obviously unsuitable for extensive green roof purposes were not included in subsequent analyses.

### 2.3. Data Analysis

The analysis followed a hierarchy, first focusing on the ordinal variable frequency [25]. Plant traits associated with highly frequent species were regarded as more important and informative. Only variables containing less than 50% missing values were considered. This procedure reduced the number of variables to fifteen. Statistical analyses were conducted using the Wilson Score Interval at 95% confidence intervals [27].

## 3. Data Analyses and Discussion

### 3.1. Species Analysis

**Figure 2:**
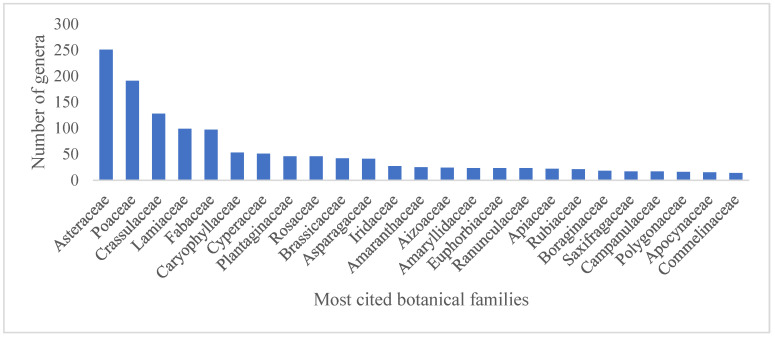
Top cited botanical families in the complete survey of green roof publications. Horizontal axis contains family names in decreasing order of mentioned genera numbers. Vertical axis indicates the number of genera mentioned per family.

**Figure 3:**
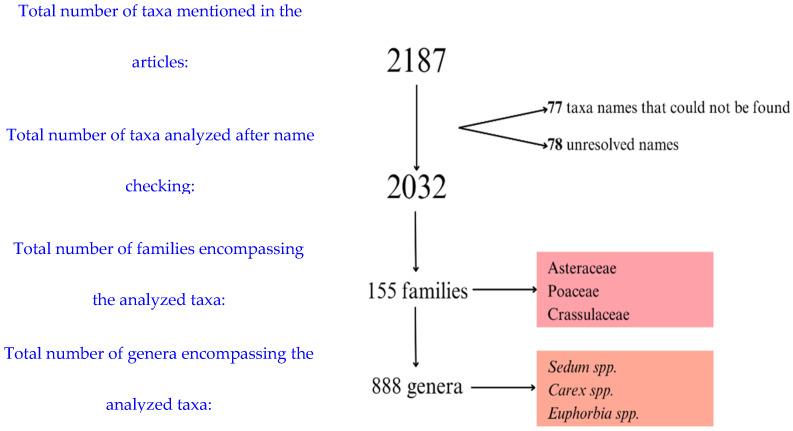
Flow chart of recorded taxa in the floristic database. Most frequently observed hits indicated in colored squares.

The review identified species used in green roofs all around the world, affording a broad database of the botanical tools applied in this technology. As a result, 439 studies were included in the qualitative synthesis (SM2). Overall, 2187 taxa were cited in the articles, including varieties and cultivars. However, after checking the species name in the Plant List [28], taxa names that could not be found (77) and unresolved names (78) were excluded. The frequency of the 2032 remaining species was analyzed. These species of green roof plants encompassed 888 genera in 155 families. Considering the entire dataset, the most cited families were Asteraceae (251 out of approx. 2812 genera), Poaceae (183 out of approx. 1768 genera), and Crassulaceae (101 out of 140 genera) (Figure 2), whereas the most cited genera were *Sedum* (109 species, Crassulaceae), *Carex* (29 species, Cyperaceae), and *Euphorbia* (26 species, Euphorbiaceae) (Figure 3). Overall, green roof studies indicate a preference for species in the triad Asteraceae, Poaceae, and Crassulaceae, which encompass a large and diverse set of species. However, it may be useful to explore species within other botanical families for use in rooftops aiming at increased diversification.

**Figure 4:**
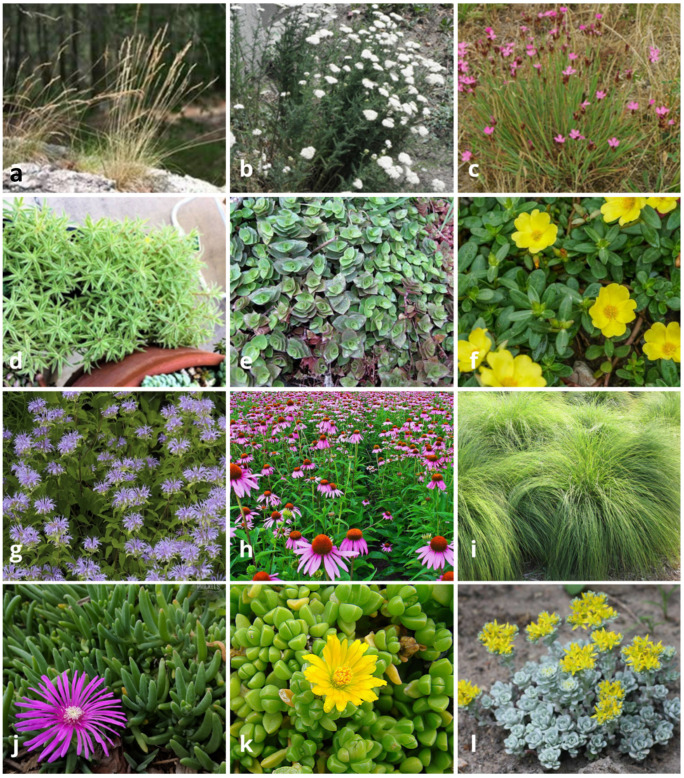
Representative species of temperate (**a**–**c**), tropical (**d**–**f**), continental (**g**–**i**), and dry (**j**–**l**) climates mentioned in articles as being useful for establishing extensive green roofs. Images are from Plants of the World Online [28} and World Flora Online [29]. (**a**). *Festuca ovina*; (**b**). *Achillea millefolium*; (**c**). *Dianthus carthusianorum*; (**d**). *Sedum lineare*; (**e**). *Callisia repens*; (**f**). *Portulaca oleracea*; (**g**). *Monarda fistulosa*; (**h**). *Echinacea purpurea*; (**i**). *Sporobolus heterolepis*; (**j**). *Delosperma cooperi*; (**k**). *Delosperma nubigenum*; (**l**). *Sedum spathulifolium*.

The thirty most frequent species for continental, dry, temperate, and tropical climates mentioned in the articles as being appropriate for use in extensive green roofs (representative examples shown in Figure 4) were chosen for analyses of their functional and morphological traits, encompassing a total of 18 features (SM3). These species names, frequency of records, and their respective families are listed for each climate in SM4.

**Figure 5:**
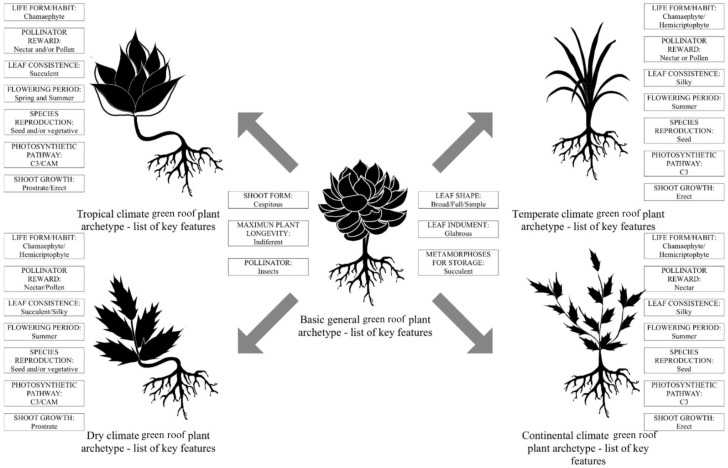
Features of the basic ideal plant archetype for use in green roofs and derived ones found as more suitable for temperate, tropical, continental, and dry climate.

Based on the records of the most used species and their respective performance described in the literature, plant trait analyses combining ideal features for green roof use in each of the four climates were discussed below. The combination of these features allowed the assembly of a putative archetype rooftop plant for temperate, tropical, continental, and dry climates (Figure 5). The main featured traits of these prototypical organisms encompass morphological, physiological, and reproductive aspects.

Some features were broadly shared by the different archetype rooftop species. Overall, these plants have glabrous broad/full/simple leaves with a linear to oblanceolate blade, often succulent. Succulence is also frequent in most cases, serving as reserve storage of water and carbon. As expected, due to constraints of the substrate, roots are not too deep and the adventitious origin of these organs is observed in several cases, which, given the thinner substrate, might be essential for successful establishment.

Regardless of climate type, the life form of rooftop plants is largely chamaephyte (38% to 47%), followed by hemicryptophyte and therophyte (Table 2). The latter is less common in green roofs of tropical climates. Geophytes and phanerophytes were the least frequent life forms of rooftop plants and no records were found for them in continental climates. In dry and continental climates there was no occurrence of phanerophytes. Dry climates had significantly lower mentions of therophyte plants (Table 2). The overall predominance of chamaephytes, which are low-growing shrubs with resting buds that are aerial but close to the substrate, may be of relevance for tolerating intense roof winds. The same reason may explain the abundance of hemicryptophytes, which essentially consist of herbaceous perennials (e.g., grasses) that produce protected buds in organs close to the substrate surface.

The shoot form of the archetypal plants included a variety of types such as branched or simple, caespitose, creeping, and only simple. However, in all climates a clear predominance of the caespitose form (i.e., dense tuft arrangement) was observed (Table 2). Branched or simple form presence was restricted to the continental climate, whereas the creeping shoot form was only recorded in the tropical climate. The caespitose form may be favored due to its potential in improving cover, reducing water evaporation, rain-induced substrate splattering, and wind damage.

The shoot growth pattern was highly variable within and among the different climates, encompassing typical and/or transitional types of ascending, erect, decumbent, prostrate, and stemless. Erect and prostrate were the overall dominant growth patterns (Table 2). Ascending, ascending to erect, and stemless were relatively less common in all climates, whereas prostrate was predominant in dry climates. Regarding leaf shape, the dominant type was clearly broad/full/simple. Leaf consistence was well distributed between silky and succulent. The latter predominated in tropical climates probably because of its improved water storage capacity. Storage adaptations in rooftop plants of most climates were succulence and succulence/rhizome (Table 2).

Seed distribution in fragmented landscapes plays a significant role in both plant species persistence and vegetation recovery. Understanding the processes by which seeds disperse in human-modified landscapes can be used to restore degraded regions more quickly, prevent habitat degeneration and biodiversity loss, and help plants and animals adapt to climate change [30]. Our knowledge of plant fecundity and seedling establishment is largely dependent on the regenerative features of seed mass and dispersal strategy [31]. The dispersal mechanism of seeds is also related to their mass; seeds larger than 100 mg are frequently carried by animals over the landscape, whereas smaller seeds are more likely to be distributed by wind or to require no assistance at all [32]. A useful proxy for determining a propagule’s capability of landscape colonization and occurrence range is its dispersal mechanism.

Temperate species are considered more likely to be wind-dispersed due to their small seed size, whereas animal-dispersed species have been reported to be more abundant in the tropics [31]. A large proportion of pollination and dispersal mode by insects was recorded for green roof species in the four climates analyzed. Overall, it is reasonable to assume that similar general principles apply to size-limited areas that are fully exposed to the elements such as green roofs. Although seed size was not evaluated, most green roof species propagate mainly through seeds, except in tropical and dry zones, where the proportion of seed and vegetative propagation is similar (circa 50%) (Table 2). Vegetative propagation is relevant in rooftops to afford a thorough substrate cover.

Winter resistance, or the species’ ability to tolerate impacts of complex environmental factors in winter and early spring periods, is regarded as the primary selection criterion when introducing useful plants under the continental climate conditions [33]. One way to survive this harsh climate and the dry conditions [34] is through seed dormancy. As evidenced by the results, a large share of green roof species in continental and dry climates relies on sexual reproduction (64% and 50%, respectively) (Table 2). The species often have nectar or pollen as a pollinator reward, except plants used for green roofs in the continental climate, in which the main pollinator reward is nectar (57%) (Table 2). Most species flower in summer and spring/summer (Table 2).

**Figure 6:**
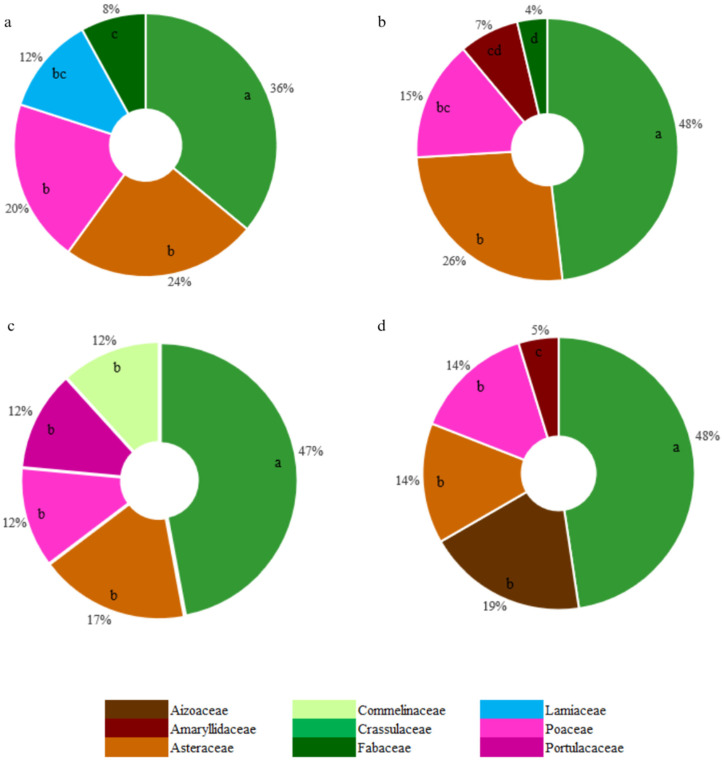
The 5 most mentioned green roof botanical families in each climate: (**a**) temperate, (**b**) continental, (**c**) tropical, and (**d**) dry. Sections not sharing a letter within each climate are significantly different (*p* ≤ 0.05).

As mentioned above, in all the climates, most green roof species were members of Crassulaceae, Asteraceae, and Poaceae (Figure 6). Tropical climates had more diverse records of families, followed by dry climates (SM5). In dry climates, there was a significant contribution of Aizoaceae.

Further examination of the data revealed that a large percentage (64.5%) of the rooftop species have medicinal (e.g., *Trifolium repens* and *Cynodon dactylon*) [35,36] or food (e.g., *Lactuca sativa* and *Portulaca oleracea*) [37,38] uses. There is also a record of some allelopathic species (e.g., *Ophiopogon japonicus* and *Achillea millefolium*) [39,40], which should be considered in the planning of rooftop vegetation composition and size. As known for most plant species, a recorded association with endophytes and/or mutualistic microorganisms, including fungi and bacteria, was also registered in the literature (e.g., *Festuca rubra* and *Trifolium arvense*) [41,42]. In addition to tolerance to drought, heat, cold, and high irradiance stresses, some species can withstand heavy metals and salt in the substrate (e.g., *Armeria maritima* and *Dianthus carthusianorum*) [43,44]. These features may be useful in dense-traffic metropolises and coastal cities.

### 3.2. Important Green Roof Plant Traits and Design Recommendations

Some traits, like CAM or C4 metabolism and succulence, are known to be relevant for the success on green roofs [6]. Because shallow soils dry up quickly and plants often have high levels of transpiration, plants on green roofs are subjected to repeated water restrictions. As pointed out before, both types of photosynthesis result in lower photorespiration and higher water use efficiency due to their carbon concentration mechanisms around RUBISCO and optimization of the stomatal aperture. However, it is well established that drought and high irradiance survival strategies vary widely, including several features that do not necessarily include the photosynthetic mechanism C4 or CAM [45,46]. On the other hand, plasticity in photosynthetic mode shifts may not always be accurately recorded in rooftop vegetation related papers.

In agreement with the observations above, quite frequently rooftop species belong to the genus *Sedum*, sharing comparable functional traits, like C3/CAM metabolism. In the present survey, most temperate climate species had a C3 photosynthetic pathway and yet are predicted to perform well on green roofs. In this case, it is possible that the importance of the photosynthetic pathway is overshadowed by other adaptive plant traits. Therefore, the presence of multiple attributes in the screening used herein may offer some novel insights that reach beyond the usually considered water storage traits. In fact, morphology may be just as significant as the photosynthetic biochemistry in the context of rooftops.

Three traits (life form, maximum plant longevity, and leaf indument) had considerable phenotypic plasticity. The results of the species list comparison showed that chamaephytes constitute the major proportion of currently applied green roof plants, and that relatively little attention has been paid to possibilities of using annual species. Chamaephytes generally have perennating buds on persistent shoots near the soil surface, often being woody plants reaching a maximum height of 25 cm above the soil surface [47]. However, therophyte and hemicryptophyte strategies also appear in species matching the requisites for rooftop life. Most therophytes germinate, grow, flower, and produce many seeds in a very short time before dying. Although not very interesting during summer months, they may be attractive for pollinators and have esthetic value in spring and early summer, due to their frequently colorful flowers [48]. Seeds produced are added to the seed bank, hence forming a buffer against eventual gap formation should other herbaceous plants die off. This property is a natural survival strategy, which may guarantee green roof performance in regions where weather conditions are very unpredictable [49]. Therefore, the incorporation of annuals in the design of green roofs can improve green roof performance and should seriously be considered.

Life form is a representation of the balance between a plant’s environment and its adaptive characteristics [47]. Hence, an adult plant’s characteristics determine its suitability for a given ecosystem. Raunkiaer’s categorization technique is merely one of several classifications systems, and it may not work well for classifying tropical plants. Our findings indicated that the proportion of chamaephytes in tropical regions was 44% (Table 2). Nevertheless, this classification undervalues significant living forms that grow in this specific region and can withstand harsh environmental conditions, such as shrubs, graminoids, vines, hemi-epiphytes, and epiphytes.

Regarding leaf indument, although glabrous was the most frequent attribute (48%, 82%, 46%, and 73% for temperate, tropical, continental, and dry climates, respectively), a great proportion of the species listed had variation among glabrous and pilous leaves (e.g., 32% for the temperate climate and 35% for the continental climate) (Table 2). Simple trichomes serve the plant in many ways, as protective and defensive structures against different stresses. Thus, the morphological and mechanical features (density, size, shape, surface texture, and orientation) of trichomes can influence many aspects of plant physiology and ecology [50]. The presence of trichomes is an important adaptation that helps decrease water loss through transpiration by stabilizing the leaf boundary layer, reducing the effects of excess irradiance on the plant (including harmful UV-B levels), increasing light reflectance, and regulating temperature [51,52].

Considering a variety of life forms for extensive green roof vegetation is a recurrent recommendation. Because of niche complementarity and facilitation, this approach might improve ecosystem functioning in terms of rainwater retention and roof cooling, as well as overall resilience to environmental stress [17]. Furthermore, even in dry conditions, extremely diversified green roofs have a greater survival rate and are typically more esthetically beautiful [18]. As a result, more than one life form species should be examined to progressively enhance green roof biodiversity without risking failure. Moreover, due to the difficult climatic circumstances in the green roof habitats, mainly drought-tolerant species should be selected [13].

### 3.3. Considerations on the Plant Trait Analysis

Although the plant trait-based approach applied in this study offered insights into the ecological background of potential green roof vegetation, the results should be interpreted with caution, as some impediments could have influenced them. First, the most extended and complete data source currently available was consulted, but still several missing values were encountered. To strengthen the analysis, species and variables with insufficient information (three species and four variables) were deleted. Indeed, potentially interesting species and traits could have been overlooked. Furthermore, substantial information was available for more general and common species (e.g., *Sedum* sp.), but was lacking for not-well-known local species. This problem is apparent in the results of the case study, with a high number of common species in the final list. Nonetheless, it is expected that, in the present study, missing traits and species did not constitute a major issue, as the purpose was not to describe a plant community but rather to give an overview of global species used in green roof systems in different climates and determine their main features.

Secondly, during the compilation of frequently used green roof species, varieties, hybrids, and subspecies were omitted because trait data are only available at the species level. This could also have caused omission of potential information. However, as mentioned before, the research objective was to find important plant traits. Furthermore, the analysis was based on 30 green roof species for each climate, so it is expected that the results are sufficiently substantiated from a statistical standpoint.

Finally, a potential issue is the phenotypic plasticity of the species. Phenotypic plasticity is the ability of an individual organism to alter its physiology and/or morphology in response to changing environmental conditions. This ability is particularly important in plants, whose sessile lifestyle requires them to deal with ambient conditions on site [53]. Other variables, such as associations with microorganisms, endophytic fungi, and bacteria, many of which have already been recorded in some of the thirty most frequent species, can potentially influence a species’ resilience and phenotypic plasticity. During the literature review, some species had different traits (leaf indument, maximum plant longevity) according to their location. Therefore, the most common feature was chosen, along with the most frequent characteristic in environments resembling green roofs.

It is important to note the existence of additional plant options for use on green roofs. Cactaceae and Bromeliaceae, for example, were not listed in our study among the five most common families, even though they have effective adaptations for the successful establishment in green roof environments. Pteridophytes, resurrection plants, and other species native to specific locations, such as coastal vegetation, mountainous areas, and rocky outcrops, as well as epiphytic plants, are also likely useful. On the other hand, the invasive potential of some species with long distance dispersion strategies should be considered when choosing green roof inhabitants.

Admittedly, the overall limited knowledge of higher numbers of species, especially for their tested performance under rooftop conditions, constitutes an inherent limitation of the present analytical strategy. Nonetheless, the approach herein outlined, along with the list of species presented, may be useful tools in the efforts to achieve more efficient, diverse, and locally adapted green roof plant communities. After testing the candidate plants under green roof conditions, nurseries could incorporate the most successful species in their supplies to meet market demands.

## 4. Conclusions

The combined analyses of several morphophysiological traits of green roof species used around the world afforded the identification of key features for successful establishment in this unique urban environment. The association of these plants with the respective climate zones in which they are used as rooftop vegetation allowed the definition of archetypes for use in dry, tropical, temperate, and continental climate green roofs. This provides a useful framework for making substantiated decisions for appropriate green roof design in different climates. The list of plants identified in this survey may assist in expanding the use of green roofs considering both sustainability and biodiversity aspects. Nonetheless, further extension of the catalog of plant species suitable for growing on rooftops is most welcome, particularly if it includes options of regional native species. Sectors to which this selection tool can be of interest (e.g., urban landscape designers, urban planning state departments, nurseries, and construction companies) will have a wider ‘menu’ of species resources to be used in extensive green roofs at their disposal.

## Figures and Tables

**Table 1:** Overview of the main functional and morphological traits analyzed.

Trait Group	Trait	Categories
Leaf adaptation	Leaf area	Small (0–500 mm^2^); large (≥501 mm^2^)
	Leaf phenology	Evergreen; winter deciduous; summer deciduous
	Leaf shape	Broad/full/simple; pinnate; palmate; digitate; needle-like; ensiform
	Leaf indument	Glabrous; pilous
	Leaf consistence	Succulent; silky
Life form	Life form/habit	Chamaephyte; hemicryptophyte; geophyte; therophyte; phanerophyte
Plant longevity	Maximum plant longevity	Annual; anual/biennial; biennial; biennial/pluriennial; pluriennial; perennial
Reserves and storage	Specialized structures for storage	None; succulent; bulb; rhizome; corm
Regeneration strategy	Adaptations for vegetative dispersion	Fragmentation; bulb; root; shoot; rhizome; corm
	Reproduction/Propagation	Seed; vegetative; seed/vegetative
	Compatibility	Compatible; incompatible
Photosynthesis	Photosynthetic pathway	C3; C4; CAM; C3/CAM; C4/CAM
Ecosystem goods or services	Flower pollinator	Wind; insects; birds; bats
	Pollinator reward	Nectar; pollen; oil
Esthetic appeal	Plant height	0–100 cm
	Flowering period	Spring; summer; winter; autumn; spring/summer; summer/winter; winter/autumn; autumn/spring; all year
	Shoot growth form	Erect; ascending to prostrate/decumbent; prostrate;
	Shoot type	Caespitose; single; climbers/lianas

**Table 2:** Percent trait occurrence in main green roof species characteristics in each climate. n = number of studies. Lowercase letters refer to trait type comparisons within the same climate; uppercase letters indicate comparisons between climates. Numbers not sharing a letter are significantly different based on Wilson Score Intervals (*p* ≤ 0.05).

		Climates							
Trait	Trait Classification	n	Temperate	n	Tropical	n	Continental	n	Dry
Vegetative Traits									
Life Form	Chamaephyte	26	38 ^a,A^	18	44 ^a,A^	18	39 ^a,A^	17	47 ^a,A^
	Geophyte		8 ^b,A^		5 ^b,A^				6 ^b,A^
	Hemicryptophyte		35 ^a,A^		17 ^ab,B^		39 ^a,A^		41 ^a,A^
	Phanerophyte		4 ^b,A^		5 ^b,A^				
	Therophyte		15 ^b,A^		28 ^a,A^		22 ^b,A^		6 ^b,B^
Shoot Form	Branched or simple	15		17		17	12 ^b^	20	
	Caespitose		100 ^A^		76 ^a,B^		82 ^a,B^		95 ^a,A^
	Creeping				5 ^b^				
	Simple				17 ^b,A^		5 ^b,B^		5 ^b,B^
Shoot Growth	Ascending	25	8 ^b,AB^	27	11 ^b,A^	25	8 ^c,AB^	23	4% ^c,B^
	Ascending to erect		8 ^b,A^		11 ^b,A^				
	Ascending to prostrate/decumbent		24 ^ab,A^		15 ^b,A^		20 ^bc,A^		8 ^bc,B^
	Erect		32 ^a,AB^		26 ^a,B^		40 ^a,A^		22 ^ab,B^
	Erect, ascending or decumbent		4 ^b,B^		7 ^b,B^		16 ^bc,A^		26 ^ab,A^
	Prostrate		16 ^ab,B^		22 ^a,B^		8 ^c,C^		39 ^a,A^
	Stemless		8 ^b,A^		7 ^b,A^		8 ^c,A^		
Leaf Shape	Broad/full/simple	30	87 ª^,A^	29	90 ª^,A^	29	90 ª^,A^	29	97 ^a,A^
	Palmate		7 ^b,A^		7 ^b,A^		7 ^b,A^		3 ^b,A^
	Pinnate		7 ^b,A^		3 ^b,A^		3 ^b,A^		
Leaf Indument	Glabrous	25	48 ^a,B^	28	82 ^a,A^	26	46 ^a,B^	22	73 ^a,AB^
	Glabrous to sparsely or fully pilous		32 ^ab,A^		7 ^b,B^		35 ^ab,A^		9 ^b,B^
	Pilous		20 ^b,A^		11 ^b,A^		19 ^b,A^		18 ^b,A^
Leaf Consistence	Silky	18	56 ^ab,A^	25	36 ^b,B^	20	55 ^a,A^	25	52 ^a,A^
	Succulent		44 ^b,B^		64 ^a,A^		45 ^a,B^		48 ^a,B^
Photosynthetic Pathway	C3	24	58 ^a,A^	22	23 ^b,B^	25	52 ^a,A^	19	26 ^b,B^
	C3/CAM		25 ^b,B^		32 ^a,AB^		24 ^b,B^		47 ^a,A^
	C4		4 ^c,B^		14 ^b,A^		12 ^b,A^		5 ^c,B^
	C4/CAM				9 ^c,A^				10 ^bc,A^
	CAM		12 ^bc,AB^		23 ^b,A^		12 ^b,AB^		5 ^c,B^
Metamorphoses for Storage	Bulb and Rhizome	15	7 ^b,A^	16	7 ^c,A^	16	12 ^b,A^	18	5 ^b,A^
	Do not have						6 ^b,A^		5 ^b,A^
	Rhizome		13 ^b,A^		7 ^c,A^		6 ^b,A^		11 ^b,A^
	Stolon				7 ^c^				
	Succulent		67 ^b,A^		62 ^a,A^		69 ^a,A^		67 ^a,A^
	Succulent and Rhizome		13 ^b,AB^		19 ^b,A^		6 ^b,B^		11 ^b,AB^
Reproductive Traits									
Maximum Longevity	Annual	27	4 ^b,A^	30		27	4 ^b,A^	28	
	Anual/Bienal		4 ^b,A^		3 ^b,A^				3 ^b,A^
	Anual/Perennial				7 ^b,A^		11 ^b,A^		11 ^b,A^
	Perennial		92 ^a,A^		90 ^a,A^		85 ^a,A^		86 ^a,A^
Reproduction/Propagation	Seed	23	52 ^a,B^	22	50 ^a,B^	22	64 ^a,A^	22	50 ^a,B^
	Seed/vegetative		43 ^a,A^		50 ^a,A^		32 ^b,B^		41 ^a,AB^
	Vegetative		4 ^b,A^				4 ^c,A^		9 ^b,A^
Flowering Period	All year	27	4 ^c,B^	24	12 ^b,A^	28		24	
	Autumn		7 ^c^						
	Spring		11 ^bc,A^		8 ^b,A^		14 ^b,A^		12 ^bc,A^
	Spring/Summer		22 ^b,AB^		33 ^a,A^		18 ^b,B^		21 ^b,AB^
	Spring/Summer/Autumn				8 ^b,A^		3 ^c,A^		4 ^c,A^
	Summer		41 ^a,A^		25 ^a,B^		39 ^a,A^		42 ^a,A^
	Summer/All year		4 ^c,A^				3 ^c,A^		
	Summer/Autumn		4 ^c,A^		8 ^b,A^		3 ^c,A^		8 ^c,A^
	Winter		7 ^c,A^		4 ^b,A^		11 ^bc,A^		4 ^c,A^
	Winter/Spring						7 ^bc,A^		8 ^c,A^
Flower Pollination	Birds	15		16	6 ^b^	18		17	
	Insects		73 ^a,A^		75 ^a,A^		67 ^a,A^		71 ^a,A^
	Insects/Birds				12 ^b,A^				6 ^b,A^
	Insects/Birds/Wind						11 ^b,A^		6 ^b,A^
	Insects/Birds/Wind/Mammals								6 ^b^
	Insects/Wind								6 ^b^
	Wind		13 ^b,AB^		6 ^b,A^		17 ^b,A^		6 ^b,A^
	Wind/Gravity		7 ^b,A^				5 ^b,A^		
	Wind/Water		7 ^b^						
Pollinator Reward	Nectar	20	45 ^a,AB^	23	35 ^a,B^	21	57 ^a,A^	20	40 ^a,AB^
	Nectar/Pollen		10 ^b,C^		30 ^a,A^		24 ^b,AB^		15 ^b,BC^
	Nectar/Pollen/Oil		5 ^b^						
	Pollen		40 ^a,A^		35 ^a,A^		19 ^b,B^		45 ^a,A^

## Data Availability

The original contributions presented in this study are included in the article and Supplementary Materials, and further inquiries can be directed to the corresponding author.

## Supplementary Materials

The following supporting information can be downloaded at: https://www.mdpi.com/article/10.3390/plants14050735/s1, Supplementary Material S1—Plant list used in this study (updated 12 September 2023). Supplementary Material S2—Full reference list. Supplementary Material S3—Plant traits. Supplementary Material S4—Plant species per climate type. Supplementary Material S5—Top mentioned green roof botanical families per climate.
