# Supplementary material for: Life at the Top: Extensive Green Roof Plant Species and Their Traits for Urban Use
Source: Plants (Basel). 2025 Feb 27;14(5):735. doi: 10.3390/plants14050735 (PMC11902263; doi:10.3390/plants14050735)
Supplement: Supplementary file 1 [file plants-14-00735-s001.zip › S1-plant list.pdf]

Supplementary Table S1-Plant list used in this study (updated 12 September 2023)

**Article Species Name**	**Family**	**Plant List Name**
Abelia × grandiflora (Ravelli ex André) Rehder	Caprifoliaceae	Unresolved
Abronia fragrans Nutt. ex Hook.	Nyctaginaceae	Accepted
Acacia confusa Merr.	Leguminosae	Accepted
Acacia cardiophylla Benth.	Leguminosae	Accepted
Acacia lasiocarpa Benth.	Leguminosae	Accepted
Acaena caesiglauca Bergmans	Rosaceae	Accepted
Acalypha australis L.	Euphorbiaceae	Accepted
Acalypha communis Müll.Arg.	Euphorbiaceae	Accepted
Acalypha poiretii Spreng.	Euphorbiaceae	Accepted
Acalypha wilkesiana Müll.Arg.	Euphorbiaceae	Accepted
Acantholimon acerosum (Willd.) Boiss.	Plumbaginaceae	Accepted
Acantholimon armenum Boiss. & A.Huet.	Plumbaginaceae	Accepted
Acantholimon litvinovii Lincz.	Plumbaginaceae	Accepted
Acer palmatum Thunb.	Sapindaceae	Accepted
Acer platanoides L.	Sapindaceae	Accepted
Acer pseudoplatanus L.	Sapindaceae	Accepted
Achillea millefolium L.	Compositae	Accepted
Achillea tomentosa L.	Compositae	Accepted
Achillea umbellata Sibth. & Sm.	Compositae	Accepted
Achyrocline bogotensis (Kunth) DC.	Compositae	Accepted
Acmispon glaber	Leguminosae	Unresolved
Acorus calamus L.	Acoraceae	Accepted
Acorus gramineus Aiton	Acoraceae	Accepted
Actinotus helianthi Labill.	Apiaceae	Accepted
Adenanthos pungens Meisn.	Proteaceae	Accepted
Adenium obesum (Forssk.) Roem. & Schult.	Apocynaceae	Accepted
Adonis aestivalis L.	Ranunculaceae	Accepted

Aechmea amicorum B.R.Silva & H.Luther.	Bromeliaceae	Accepted
Aechmea cefaloides J.A.Siqueira & Leme.	Bromeliaceae	Accepted
Aechmea pectinata Baker.	Bromeliaceae	Accepted
Aeonium arboreum Webb & Berthel.	Crassulaceae	Accepted
Aeonium subplanum	Crassulaceae	Synonym of *Aeonium canariense var. subplanum* (Praeger) H.Y.Liu.
Aeschynomene indica L.	Leguminosae	Accepted
Aethionema saxatile (L.) R.Br.	Brassicaceae	Accepted
Aethionema schistosum Boiss. & Kotschy.	Brassicaceae	Accepted
Agalinis communis (Cham. & Schltdl.) D'Arcy	Orobanchaceae	Accepted
Agapanthus praecox Willd.	Amaryllidaceae	Accepted
Agastache foeniculum (Pursh) Kuntze	Lamiaceae	Accepted
Agastache rugosa (Fisch. & C.A.Mey.) Kuntze.	Lamiaceae	Accepted
Agastache rupestris (Greene) Standl.	Lamiaceae	Accepted
Agave attenuata Salm-Dyck	Asparagaceae	Accepted
Agave gypsophylla Gentry.	Asparagaceae	Accepted
Agave parryi Engelm.	Asparagaceae	Accepted
Agave stricta Salm-Dyck	Asparagaceae	Accepted
Agave victoriae-reginae T.Moore	Asparagaceae	Accepted
Ageratum conyzoides (L.) L.	Compositae	Accepted
Ageratum houstonianum Mill.	Compositae	Accepted
Ageratum hybrid 'Artist blue'	Compositae	No results
Aglaia odorata Lour.	Meliaceae	Accepted
Agrimonia eupatoria L.	Rosaceae	Accepted
Agropyron cristatum (L.) Gaertn.	Poaceae	Accepted

Agrostemma githago L.	Caryophyllaceae	Accepted
Agrostemma gracile	Caryophyllaceae	Synonym of *Agrostemma brachyloba* (Fenzl) K.Hammer
Agrostis capillaris L.	Poaceae	Accepted
Agrostis hyemalis (Walter) Britton, Sterns & Poggenb.	Poaceae	Accepted
Agrostis pallens Trin.	Poaceae	Accepted
Agrostis stolonifera L.	Poaceae	Accepted
Agrostis tenuis	Poaceae	Synonym of *Agrostis idahoensis* Nash.
Ailanthus altissima (Mill.) Swingle	Simaroubaceae	Accepted
Aira caryophyllea L.	Poaceae	Accepted
Ajuga ciliata Bunge	Lamiaceae	Accepted
Ajuga genevensis L.	Lamiaceae	Accepted
Ajuga reptans L.	Lamiaceae	Accepted
Alliaria petiolata (M.Bieb.) Cavara & Grande	Brassicaceae	Accepted
Aletris farinosa L.	Nartheciaceae	Accepted
Allamanda cathartica L.	Apocynaceae	Accpeted
Allium acuminatum Hook.	Amaryllidaceae	Accepted
Allium ampeloprasum L.	Amaryllidaceae	Accepted
Allium caeruleum Pall.	Amaryllidaceae	Accepted
Allium canadense L.	Amaryllidaceae	Accepted
Allium carinatum L.	Amaryllidaceae	Accepted
Allium cepa L.	Amaryllidaceae	Accepted
Allium cernuum Roth.	Amaryllidaceae	Accepted
Allium flavum L.	Amaryllidaceae	Accepted
Allium karataviense Regel.	Amaryllidaceae	Accepted
Allium oleraceum L.	Amaryllidaceae	Accepted
Allium ostrowskianum	Amaryllidaceae	Synonym of *Allium oreophilum* C.A.Mey.
Allium roseum L.	Amaryllidaceae	Accepted

Allium sativum L.	Amaryllidaceae	Accepted
Allium schoenoprasum L.	Amaryllidaceae	Accepted
Allium senescens L.	Amaryllidaceae	Accepted
Allium sphaerocephalon L.	Amaryllidaceae	Accepted
Allium stellatum Nutt. ex Ker Gawl.	Amaryllidaceae	Accepted
Allium triquetrum L.	Amaryllidaceae	Accepted
Allium unifolium Kellogg.	Amaryllidaceae	Accepted
Alocasia 'Amazonica'	Araceae	Synonym of *Alocasia* × *mortfontanensis* André.
Aloe 'Always Red	Xanthorrhoeaceae	No results
Aloe 'Aries'	Xanthorrhoeaceae	No results
Aloe barbadensis Mill.	Xanthorrhoeaceae	Synonym of *Aloe vera* (L.) Burm.f.
Aloe mitriformis Mill.	Xanthorrhoeaceae	Synonym of *Aloe perfoliata* L.
Aloe 'Southern Cross'	Xanthorrhoeaceae	No results
Aloe 'Topaz'	Xanthorrhoeaceae	No results
Aloe vera (L.) Burm.f.	Xanthorrhoeaceae	Accepted
Alopecurus pratensis L.	Poaceae	Accepted
Alternanthera bettzickiana (Regel) G.Nicholson	Amaranthaceae	Accepted
Alternanthera dentata Stuchlík ex R.E.Fr.	Amaranthaceae	Synonym of *Alternanthera ramosissima* (Mart.) Chodat & Hassl.
Alternanthera ficoidea (L.) Sm.	Amaranthaceae	Accepted
Alternanthera paronychioides A.St.-Hil.	Amaranthaceae	Accepted

Alyogyne huegelii (Endl.) Fryxell	Malvaceae	Accepted
Alysicarpus vaginalis (L.) DC.	Leguminosae	Accepted
Alyssum alyssoides (L.) L.	Brassicaceae	Accepted
Alyssum maritimum	Brassicaceae	Synonym of *Lobularia maritima* (L.) Desv.
Alyssum montanum L.	Brassicaceae	Accepted
Amaranthus albus L.	Amaranthaceae	Accepted
Amaranthus blitoides S.Watson	Amaranthaceae	Accepted
Amaranthus cruentus L.	Amaranthaceae	Accepted
Amaranthus dubius Mart. ex Thell.	Amaranthaceae	Accepted
Amaranthus retroflexus L.	Amaranthaceae	Accepted
Amaranthus tricolor L.	Amaranthaceae	Accepted
Amaranthus tuberculatus (Moq.) Sauer	Amaranthaceae	Accepted
Ambrosia artemisiifolia L.	Compositae	Accepted
Ambrosia chamissonis (Less.) Greene.	Compositae	Accepted
Ammobium alatum R.Br.	Compositae	Accepted
Amorpha canescens Pursh.	Leguminosae	Accepted
Amorpha fruticosa L.	Leguminosae	Accepted
Amorpha nana C.Fraser.	Leguminosae	Accepted
Amsonia tabernaemontana var. salicifolia (Pursh) Woodson.	Apocynaceae	Accepted
Anacamptis pyramidalis (L.) Rich.	Orchidaceae	Accepted
Anacyclus depressus Ball	Compositae	Synonym of *Anacyclus pyrethrum* (L.) Lag.
Anacyclus maroccanus (Ball) Ball.	Compositae	Accepted
Anagallis arvensis L.	Primulaceae	Accepted
Anagallis monelli L.	Primulaceae	Accepted
Anaphalis margaritacea (L.) Benth. & Hook.f.	Compositae	Accepted
Anastatica hierochunta L.	Brassicaceae	Accepted
Andropogon gerardii Vitman.	Poaceae	Accepted

Andropogon gyrans Ashe.	Poaceae	Accepted
Andropogon ternarius Michx.	Poaceae	Accepted
Andropogon virginicus L.	Poaceae	Accepted
Anemone canadensis L.	Ranunculaceae	Accepted
Anemone caroliniana Walter.	Ranunculaceae	Accepted
Anemone cylindrica A.Gray.	Ranunculaceae	Accepted
Anemone hortensis L.	Ranunculaceae	Accepted
Anemone patens L.	Ranunculaceae	Accepted
Anemone pulsatilla L.	Ranunculaceae	Accepted
Anemone sylvestris L.	Ranunculaceae	Accepted
Anemone virginiana L.	Ranunculaceae	Accepted
Anethum graveolens L.	Apiaceae	Accepted
Anigozanthos 'Big Red'	Haemodoraceae	No results
Anoda cristata (L.) Schltdl.	Malvaceae	Accepted
Anredera cordifolia (Ten.) Steenis	Basellaceae	Accepted
Antennaria dioica (L.) Gaertn.	Compositae	Accepted
Antennaria neglecta Greene.	Compositae	Accepted
Antennaria parvifolia 'McClintock'	Compositae	Variety of *Antennaria parvifolia* Nutt.
Antennaria parvifolia Nutt.	Compositae	Accepted
Antennaria plantaginifolia (L.) Richardson	Compositae	Accepted
Anthemis arvensis L.	Compositae	Accepted
Anthemis maritima L.	Compositae	Accepted
Anthemis pseudocotula Boiss.	Compositae	Accepted
Anthemis tinctoria	Compositae	Synonym of *Cota tinctoria* (L.) J.Gay.
Anthericum liliago L.	Asparagaceae	Accepted
Anthoxanthum aristatum Boiss.	Poaceae	Accepted
Anthoxanthum occidentale	Poaceae	Synonym of *Hierochloe occidentalis* Buckley.
Anthoxanthum odoratum L.	Poaceae	Accepted
Anthyllis montana L.	Leguminosae	Accepted

Anthyllis vulneraria L.	Leguminosae	Accepted
Antigonon leptopus Hook. & Arn.	Polygonaceae	Accepted
Antirrhinum linkianum Boiss. & Reut.	Plantaginaceae	Synonym of *Antirrhinum majus subsp. linkianum* (Boiss. & Reut.) Rothm.
Antirrrinum majus L.	Plantaginaceae	Accepted
Antitrichia californica Sull.	Leucodontaceae	Accepted
Anundo donax var. veriscolor	Poaceae	Synonym of *Arundo donax* L.
Aphanes arvensis L.	Rosaceae	Accepted
Apium graveolens L.	Apiaceae	Accepted
Aptenia cordifolia	Aizoaceae	Synonym of *Mesembryanthemum cordifolium* L.f.
Aptenia lancifolia L.Bolus	Aizoaceae	Synonym of *Mesembryanthemum lancifolium* (L.Bolus) Klak
Aquilegia canadensis L.	Ranunculaceae	Accepted
Aquilegia formosa Fisch. ex DC.	Ranunculaceae	Accepted
Aquilegia vulgaris L.	Ranunculaceae	Accepted
Arabidopsis thaliana (L.) Heynh.	Brassicaceae	Accepted
Arabis alpina L.	Brassicaceae	Accepted
Arabis alpina ssp. caucasica	Brassicaceae	Synonym of *Arabis caucasica* Willd.
Arabis collina Ten.	Brassicaceae	Accepted
Arabis lyrata	Brassicaceae	Synonym of *Arabidopsis lyrata* (L.) O'Kane & Al-Shehbaz.
Arachis duranensis Krapov. & W.C.Greg.	Leguminosae	Accepted
Arachis hypogaea L.	Leguminosae	Accepted
Arachis pintoi Krapov. & W.C.Greg.	Leguminosae	Accepted

Arachis repens Handro	Leguminosae	Accepted
Arbutus unedo L.	Ericaceae	Accepted
Arctostaphyllos uva-ursi (L.) Spreng.	Ericaceae	Accepted
Arctostaphylos edmundsii 'Big Sur'	Ericaceae	Variety of *Arctostaphylos edmundsii* J.T.Howell
Arctostaphylos patula Greene.	Ericaceae	Accepted
Arctostaphylos x coloradoensis 'Cascade'	Ericaceae	Unresolved
Arenaria alfacarensis Pamp.	Caryophyllaceae	Accepted
Arenaria serpyllifolia L.	Caryophyllaceae	Accepted
Arenaria stricta	Caryophyllaceae	Unresolved
Argemone polyanthemos (Fedde) Ownbey	Papaveraceae	Accepted
Argyrolobium zanonii (Turra) P.W.Ball	Leguminosae	Accepted
Arisaema triphyllum (L.) Schott	Araceae	Accepted
Aristida oligantha Michx.	Poaceae	Accepted
Armeria arenaria (Pers.) Schult.	Plumbaginaceae	Accepted
Armeria juniperifolia (Vahl) Hoffmanns. & Link.	Plumbaginaceae	Accepted
Armeria marítima (Mill.) Willd.	Plumbaginaceae	Accepted
Armeria maritima ssp. californica (Boiss.) Porsild.	Plumbaginaceae	Accepted
Armeria maritima subsp. maritima	Plumbaginaceae	Synonym of *Armeria maritima* (Mill.) Willd.
Armeria pungens (Link) Hoffmanns. & Link.	Plumbaginaceae	Accepted
Arrhenatherum elatius (L.) P.Beauv. ex J.Presl & C.Presl.	Poaceae	Accepted
Artemisia absinthium L.	Compositae	Accepted
Artemisia alba Turra	Compositae	Accepted
Artemisia annua L.	Compositae	Accepted
Artemisia californica Less.	Compositae	Accepted

Artemisia campestris L.	Compositae	Accepted
Artemisia campestris subsp. caudata (Michx.) H.M.Hall & Clem.	Compositae	Accepted
Artemisia caudata	Compositae	Synonym of *Artemisia campestris subsp. caudata* (Michx.) H.M.Hall & Clem.
Artemisia douglasiana Besser ex Besser.	Compositae	Accepted
Artemisia frigida Willd.	Compositae	Accepted
Artemisia judaica L.	Compositae	Accepted
Artemisia indica Willd.	Compositae	Accepted
Artemisia ludoviciana Nutt.	Compositae	Accepted
Artemisia pycnocephala (Less.) DC.	Compositae	Accepted
Artemisia sieberi Besser.	Compositae	Accepted
Artemisia stelleriana Besser.	Compositae	Accepted
Artemisia vulgaris L.	Compositae	Accepted
Arthrocnemum macrostachyum (Moric.) K.Koch	Amaranthaceae	Accepted
Arthropodium milleflorum (DC.) J.F.Macbr.	Asparagaceae	Accepted
Arthropodium minus R.Br.	Asparagaceae	Accepted
Arthropodium strictum R.Br.	Asparagaceae	Synonym of *Dichopogon strictus* (R.Br.) Baker
Asarum caudatum Lindl.	Aristolochiaceae	Accepted
Asclepias curassavica L.	Apocynaceae	Accepted
Asclepias purpurascens L.	Apocynaceae	Accepted
Asclepias syriaca L.	Apocynaceae	Accepted
Asclepias tuberosa L.	Apocynaceae	Accepted
Asclepias verticillata L.	Apocynaceae	Accepted
Asclepias viridiflora Raf.	Apocynaceae	Accepted
Asparagus densiflorus (Kunth) Jessop	Asparagaceae	Accepted

Asparagus plumosus	Asparagaceae	Synonym of *Asparagus setaceus* (Kunth) Jessop.
Asphodelus fistulosus L.	Xanthorrhoeaceae	Accepted
Aster alpinus L.	Compositae	Accepted
Aster amellus L.	Compositae	Accepted
Aster laevis	Compositae	Synonym of *Symphyotrichum laeve* (L.) Á.Löve & D.Löve.
Aster linosyris	Compositae	Synonym of *Galatella linosyris* (L.) Rchb.f.
Aster novaeangliae	Compositae	Synonym of *Symphyotrichum novae-angliae* (L.) G.L.Nesom.
Aster novae-belgii	Compositae	Synonym of *Symphyotrichum novi-belgii* (L.) G.L.Nesom.
Aster oolentangiensis	Compositae	Synonym of *Symphyotrichum oolentangiense* (Riddell) G.L.Nesom.
Aster pilosus	Compositae	Synonym of *Symphyotrichum pilosum* (Willd.) G.L.Nesom.
Aster subulatus (Michx.) Hort. ex Michx.	Compositae	Accepted
Asteriscus maritimus	Compositae	Synonym of *Pallenis maritima* (L.) Greuter.
Astilbe 'Purple Lance'	Saxifragaceae	Synonym of *Astilbe rubra* Hook.f. & Thomson.
Astragalus agrestis G.Don.	Leguminosae	Accepted
Astragalus alpinus L.	Leguminosae	Accepted
Astragalus canadensis L.	Leguminosae	Accepted

Astragalus nuttallii (Torr. & A.Gray) J.T.Howell.	Leguminosae	Accepted
Astragalus tennesseensis Chapman.	Leguminosae	Accepted
Astralagus crassicarpus Nutt.	Leguminosae	Accepted
Athamanta cretensis L.	Apiaceae	Accepted
Atrichum undulatum (Hedw.) P. Beauv.	Polytrichaceae	Accepted
Atriplex confertifolia (Torr. & Frém.) S.Watson.	Amaranthaceae	Accepted
Atriplex halimus L.	Amaranthaceae	Accepted
Atriplex lentiformis (Torr.) S.Watson	Amaranthaceae	Accepted
Atriplex patula L.	Amaranthaceae	Accepted
Atriplex portulacoides L.	Amaranthaceae	Accepted
Atriplex prostrata Boucher ex DC.	Amaranthaceae	Accepted
Atriplex semibaccata R.Br.	Amaranthaceae	Accepted
Aurinia saxatilis (L.) Desv.	Brassicaceae	Accepted
Austrodanthonia caespitosa	Poaceae	Synonym of *Rytidosperma caespitosum* (Gaudich.) Connor & Edgar.
Austrodanthonia carphoides (Benth.) H.P.Linder	Poaceae	Synonym of *Rytidosperma carphoides* (F.Muell. ex Benth.) Connor & Edgar
Austrodanthonia setacea	Poaceae	Synonym of *Rytidosperma setaceum* (R.Br.) Connor & Edgar.
Austrostipa densiflora (Hughes) S.W.L.Jacobs & J.Everett	Poaceae	Synonym of *Stipa densiflora* Hughes
Austrostipa elegantissima (Labill.) S.W.L.Jacobs & J.Everett	Poaceae	Synonym of *Stipa elegantissima* Labill.
Austrostipa mollis (R.Br.) S.W.L.Jacobs & J.Everett	Poaceae	Synonym of *Stipa mollis* R.Br.

Austrostipa scabra	Poaceae	Synonym of *Stipa scabra* Lindl.
Avena barbata Pott ex Link.	Poaceae	Accepted
Avena sterilis L.	Poaceae	Accepted
Avenella flexuosa (L.) Drejer		Synonym of *Deschampsia flexuosa* (L.) Trin.
Axonopus compressus (Sw.) P.Beauv.	Poaceae	Accepted
Baccharis pilularis DC.	Compositae	Accepted
Baccharis pilularis 'Twin Peaks'	Compositae	Variety of *Baccharis pilularis* DC.
Bacopa monnieri (L.) Wettst.	Plantaginaceae	Accepted
Baeckea astarteoides Benth.	Myrtaceae	Synonym of *Astartea astarteoides* (Benth.) Rye
Baileya multiradiata Harv. & A.Gray ex Torr.	Compositae	Accepted
Ballota acetabulosa (L.) Benth.	Lamiaceae	Accepted
Ballota nigra L.	Lamiaceae	Accepted
Banksia baueri R.Br.	Proteaceae	Accepted
Banksia nivea Labill.	Proteaceae	Accepted
Banksia petiolaris F.Muell.	Proteaceae	Accepted
Baptisia alba (L.) Vent.	Leguminosae	Accepted
Baptisia australis (L.) R.Br.	Leguminosae	Accepted
Baptisia tinctoria (L.) Vent.	Leguminosae	Accepted
Barbarea vulgaris R.Br.	Brassicaceae	Accepted
Barleria repens Nees	Acanthaceae	Accepted
Bauhinia coccinea (Lour.) DC.	Leguminosae	Accepted
Bauhinia kockiana Korth.	Leguminosae	Accepted
Belamcanda chinensis	Iridaceae	Synonym of *Iris domestica* (L.) Goldblatt & Mabb.
Bellis perennis L.	Compositae	Accepted

Belosynapsis ciliata (Blume) R.S.Rao.	Commelinaceae	Accepted
Bergenia cordifolia	Saxifragaceae	Synonym of *Bergenia crassifolia* (L.) Fritsch
Berteroa incana (L.) DC.	Brassicaceae	Accepted
Beta vulgaris L.	Amaranthaceae	Accepted
Betula pubescens Ehrh.	Betulaceae	Accepted
Bidens alba (L.) DC.	Compositae	Synonym of *Bidens pilosa* L.
Bidens pilosa L.	Compositae	Accepted
Bidens subalternans DC.	Compositae	Accepted
Biscutella laevigata L.	Brassicaceae	Accepted
Blackstonia perfoliata (L.) Huds.	Gentianaceae	Accepted
Blumea mollis	Compositae	Synonym of *Blumea axillaris* (Lam.) DC.
Bossiaea prostrata R.Br.	Leguminosae	Accepted
Bothriospermum zeylanicum	Boraginaceae	Synonym of *Bothriospermum tenellum* (Hornem.) Fisch. & C.A.Mey.
Bougainvillea 'sakura variegata'	Nyctaginaceae	No results
Bouteloua curtipendula (Michx.) Torr.	Poaceae	Accepted
Bouteloua gracilis	Poaceae	Synonym of *Chondrosum gracile* Kunth.
Bouteloua hirsuta	Poaceae	Synonym of *Chondrosum hirsutum* (Lag.) Sweet.
Bouteloua rigidiseta (Steud.) Hitchc.	Poaceae	Accepted
Brachypodium distachyon (L.) P.Beauv.	Poaceae	Accepted
Brachypodium phoenicoides (L.) Roem. & Schult.	Poaceae	Accepted

Brachypodium retusum (Pers.) P.Beauv.	Poaceae	Accepted
Brachyscome angustifolia A.Cunn. ex DC.	Compositae	Accepted
Brachyscome basaltica F.Muell.	Compositae	Accepted
Brachyscome formosa P.S.Short	Compositae	Accepted
Brachyscome multifida DC.	Compositae	Accepted
Brachythecium salebrosum (Hoffm. ex F. Weber & D. Mohr) Schimp.	Brachytheciaceae	Accepted
Brassica oleraceae L.	Brassicaceae	Accepted
Brassica oleracea var. sabauda L.	Brassicaceae	Synonym of *Brassica oleracea* L.
Brassica rapa L.	Brassicaceae	Accepted
Braya alpina Sternb. & Hoppe	Brassicaceae	Accepted
Briza maxima L.	Poaceae	Accepted
Briza media L.	Poaceae	Accepted
Brodiaea coronária (Salisb.) Jeps.	Asparagaceae	Accepted
Brodiaea hyancinthina	Asparagaceae	Synonym of *Triteleia hyacinthina* (Lindl.) Greene.
Bromus erectus Huds.	Poaceae	Accepted
Bromus hordeaceus L.	Poaceae	Accepted
Bromus inermis Leyss.	Poaceae	Accepted
Bromus madritensis L.	Poaceae	Accepted
Bromus rubens L.	Poaceae	Accepted
Bromus sterilis L.	Poaceae	Accepted
Bromus tectorum L.	Poaceae	Accepted
Brunonia australis R.Br.	Goodeniaceae	Accepted
Bryophyllum daigremontianum (Raym.-Hamet & Perrier) A.Berger	Crassulaceae	Accepted
Bryophyllum pinnatum (Lam.) Oken.	Crassulaceae	Accepted
Bryum argenteum Hedw.	Bryaceae	Accepted
Buchloe dactyloides (Nutt.) Engelm.	Poaceae	Accepted

Buddleja davidii Franch.	Scrophulariaceae	Accepted
Bulbine bulbosa (R.Br.) Haw.	Xanthorrhoeaceae	Accepted
Bulbine caulescens L.	Xanthorrhoeaceae	Synonym of *Bulbine frutescens* (L.) Willd.
Bulbine frutescens (L.) Willd.	Xanthorrhoeaceae	Accepted
Bulbine vagans E.M.Watson	Xanthorrhoeaceae	Accepted
Bupleurum falcatum L.	Apiaceae	Accepted
Burchardia umbellata R.Br.	Colchicaceae	
Cajanus scarabaeoides (L.) Thouars	Leguminosae	Accepted
Cakile edentula (Bigelow) Hook.	Brassicaceae	Accepted
Calamagrostis × acutiflora (Schrad.) DC.	Poaceae	Accepted
Calamagrostis brachytricha	Poaceae	Synonym of *Calamagrostis arundinacea* (L.) Roth.
Calamagrostis epigejos (L.) Roth.	Poaceae	Accepted
Calamagrostis effusa (Kunth) Steud.	Poaceae	Accepted
Calamagrostis pickeringii A.Gray.	Poaceae	Accepted
Calamagrostis stricta (Timm) Koeler.	Poaceae	Accepted
Calamintha arkansana (Nutt.) Kuntze.	Lamiaceae	Accepted
Calamintha nepeta	Lamiaceae	Synonym of *Clinopodium nepeta* (L.) Kuntze.
Calandrinia polyandra Benth.	Portulacaceae	Accepted
Calendula arvensis M.Bieb.	Compositae	Accepted
Callirhoe involucrata (Torr. & A.Gray) A.Gray.	Malvaceae	Accepted
Callisia fragrans (Lindl.) Woodson.	Commelinaceae	Accepted
Callisia repens (Jacq.) L.	Commelinaceae	Accepted

Callisia warszewicziana (Kunth & C.D.Bouché) D.R.Hunt.	Commelinaceae	Accepted
Callistemon citrinus (Curtis) Skeels	Myrtaceae	Accepted
Calocephalus citreus Less.	Compositae	Accepted
Calothamnus graniticus Hawkeswood	Myrtaceae	Accepted
Calothamnus hirsutus Hawkeswood	Myrtaceae	Accepted
Calylophus hartwegii (Benth.) P.H.Raven	Onagraceae	Accepted
Calystegia sepium (L.) R. Br.	Convolvulaceae	Accepted
Calytrix tetragona Labill.	Myrtaceae	Accepted
Camassia quamash (Pursh) Greene.	Asparagaceae	Accepted
Campanula americana L.	Campanulaceae	Accepted
Campanula cochleariifolia Lam.	Campanulaceae	Accepted
Campanula glomerata L.	Campanulaceae	Accepted
Campanula incurva Aucher ex A.DC.	Campanulaceae	Accepted
Campanula kemulariae	Campanulaceae	Synonym of *Campanula raddeana* Trautv.
Campanula persicifolia L.	Campanulaceae	Accepted
Campanula portenschlagiana Schult.	Campanulaceae	Accepted
Campanula poscharskyana Degen.	Campanulaceae	Accepted
Campanula rotundifolia L.	Campanulaceae	Accepted
Campanula spicata L.	Campanulaceae	Accepted
Campanula trachelium L.	Campanulaceae	Accepted
Campanulastrum americanum	Campanulaceae	Synonym of *Campanula americana* L.
Canna indica L.	Cannaceae	Accepted
Capsella bursa-pastoris (L.) Medik.	Brassicaceae	Accepted
Capsicum annuum L.	Solanaceae	Accepted
Cardamine flexuosa With.	Brassicaceae	Accepted
Cardamine hirsuta L.	Brassicaceae	Accepted
Carex alba Scop.	Cyperaceae	Accepted

Carex arenaria L.	Cyperaceae	Accepted
Carex argyranthra Tuck. ex Boott.	Cyperaceae	Accepted
Carex bicknelli Britton & A.Br.	Cyperaceae	Accepted
Carex densa (L.H.Bailey) L.H.Bailey.	Cyperaceae	Accepted
Carex eburnea Boott.	Cyperaceae	Accepted
Carex flacca Schreb.	Cyperaceae	Accepted
Carex fagellifera 'Bronzita'	Cyperaceae	Variety of *Carex flagellifera* Colenso
Carex glaucodea Tuck. ex Olney.	Cyperaceae	Accepted
Carex gracillima Schwein.	Cyperaceae	Accepted
Carex halleriana Asso.	Cyperaceae	Accepted
Carex hirta L.	Cyperaceae	Accepted
Carex inops L.H.Bailey.	Cyperaceae	Accepted
Carex meridiana Akiyama.	Cyperaceae	Accepted
Carex mucronata All.	Cyperaceae	Accepted
Carex muhlenbergii Willd.	Cyperaceae	Accepted
Carex nigra (L.) Reichard.	Cyperaceae	Accepted
Carex oshimensis Nakai.	Cyperaceae	Accepted
Carex pachystachya	Cyperaceae	Synonym of *Carex macloviana subsp. pachystachya* (Cham. ex Steud.) Hultén.
Carex pansa L.H.Bailey	Cyperaceae	Accepted
Carex pensylvanica Lam.	Cyperaceae	Accepted
Carex praegracilis W.Boott.	Cyperaceae	Accepted
Carex radiata (Wahlenb.) Small.	Cyperaceae	Accepted
Carex retroflexa Muhl. ex Willd.	Cyperaceae	Accepted
Carex sprengelii Dewey ex Spreng.	Cyperaceae	Accepted
Carex sylvatica Huds.	Cyperaceae	Accepted
Carex texensis (Torr. ex L.H.Bailey) L.H.Bailey.	Cyperaceae	Accepted
Carex tumulicola Mack.	Cyperaceae	Accepted

Carex vulpinoidea Michx.	Cyperaceae	Accepted
Carpinus betulus L.	Betulaceae	Accepted
Carpobrotus acinaciformis (L.) L.Bolus.	Aizoaceae	Accepted
Carpobrotus edulis (L.) N.E.Br.	Aizoaceae	Accepted
Carpobrotus glaucescens (Haw.) Schwantes	Aizoaceae	Accepted
Carpobrotus modestus S.T.Blake.	Aizoaceae	Accepted
Carpobrotus rossii (Haw.) Schwantes.	Aizoaceae	Accepted
Carthamus carduncellus L.	Compositae	Accepted
Cassia fasciculata	Leguminosae	Synonym of *Chamaecrista fasciculata* (Michx.) Greene.
Castilleja coccínea (L.) Spreng.	Orobanchaceae	Accepted
Castilleja integra A.Gray.	Orobanchaceae	Accepted
Catapodium rigidum (L.) C.E.Hubb.	Poaceae	Accepted
Catharanthus roseus (L.) G.Don.	Apocynaceae	Accepted
Ceanothus americanUs L.	Rhamnaceae	Accepted
Ceanothus maritimus Hoover.	Rhamnaceae	Accepted
Ceanothus thyrsiflorus var. griseus (Trel. ex B.L.Rob.) McMinn.	Rhamnaceae	Accepted
Celtis australis L.	Cannabaceae	Accepted
Celtis sinensis Pers.	Cannabaceae	Accepted
Celosia argentea L.	Amaranthaceae	Accepted
Centaurea cyanus	Compositae	Synonym of *Cyanus segetum* Hill.
Centaurea jacea L.	Compositae	Accepted
Centaurea nigra L.	Compositae	Accepted
Centaurea scabiosa L.	Compositae	Accepted
Centaurium erythraea Rafn.	Gentianaceae	Accepted
Centella asiática (L.) Urb.	Apiaceae	Accepted

Centipeda mínima (L.) A.Braun & Asch.	Compositae	Accepted
Centranthus calcitrapa (L.) Dufr.	Caprifoliaceae	Accepted
Centranthus macrosiphon Boiss	Caprifoliaceae	Accepted
Centranthus ruber (L.) DC.	Caprifoliaceae	Accepted
Centranthus ruber subsp. sibthorpii (Heldr. & Sart. ex Boiss.) Hayek	Caprifoliaceae	Accepted
Cephalotaxus harringtonii (Knight ex J.Forbes) K.Koch	Taxaceae	Accepted
Cerastium alpinum L.	Caryophyllaceae	Accepted
Cerastium arvense L.	Caryophyllaceae	Accepted
Cerastium biebersteinii DC.	Caryophyllaceae	Accepted
Cerastium fontanum Baumg.	Caryophyllaceae	Accepted
Cerastium fontanum subsp. vulgare (Hartm.) Greuter & Burdet.	Caryophyllaceae	Accepted
Cerastium glomeratum Thuill.	Caryophyllaceae	Accepted
Cerastium holosteoides Fr.	Caryophyllaceae	Synonym of *Cerastium fontanum subsp. vulgare* (Hartm.) Greuter & Burdet
Cerastium pumilum Curtis.	Caryophyllaceae	Accepted
Cerastium pumilum var. glutinosum (Čelak.) E.Rico.	Caryophyllaceae	Accepted
Cerastium semidecandrum L.	Caryophyllaceae	Accepted
Cerastium tomentosum L.	Caryophyllaceae	Accepted
Cerastium velutinum Raf.	Caryophyllaceae	Accepted
Cerastium velutinum var. villosissimum (Pennell) J.K.Morton.	Caryophyllaceae	Accepted
Cerastium vulgatum	Caryophyllaceae	Synonym of *Cerastium glomeratum* Thuill.
Ceratophyllum demersum L.	Ceratophyllaceae	Accepted
Ceratodon purpureus (Hedw.) Brid.	Ditrichaceae	Accepted
Cercocarpus breviflorus	Rosaceae	Unresolved

Ceterach officinarum Willd.	Aspleniaceae	Accepted
Chaetosciadium trichospermum (L.) Boiss.	Apiaceae	Accepted
Chamaebatiaria millefolium (Torr.) Maxim.	Rosaceae	Accepted
Chamaecrista fasciculata (Michx.) Greene.	Leguminosae	Accepted
Chamaedorea seifrizii Burret.	Arecaceae	Accepted
Chamaemelum nobile (L.) All.	Compositae	Accepted
Chamaesyce maculata	Euphorbiaceae	Synonym of *Euphorbia maculata* L.
Chamelaucium ciliatum Desf.	Myrtaceae	Accepted
Chamomilla recutita	Compositae	Synonym of *Matricaria chamomilla* L.
Chasmanthium latifolium (Michx.) H.O.Yates	Poaceae	Accepted
Cheilanthes austrotenuifolia H.Quirk & T.C.Chambers		Unresolved
Cheiranthera linearis A.Cunn. ex Lindl.	Pittosporaceae	Accepted
Chelidonium majus L.	Papaverceae	Accepted
Chenopodium album L.	Amaranthaceae	Accepted
Chenopodium carinatum R.Br.	Amaranthaceae	Unresolved
Chenopodium pumilio R.Br.	Amaranthaceae	Synonym of *Dysphania pumilio* (R.Br.) Mosyakin & Clemants
Chilopsis linearis (Cav.) Sweet.	Bignoniaceae	Accepted
Chloris gayana Kunth	Poaceae	Accepted
Chloris halophila Parodi	Poaceae	Accepted
Chloris truncata R.Br.	Poaceae	Accepted
Chlorophytum comosum (Thunb.) Jacques	Asparagaceae	Accepted
Chrysactinia mexicana A.Gray.	Compositae	Accepted

Chrysanthemum × morifolium 'Golden times'	Compositae	Unresolved
Chrysanthemum coronarium	Compositae	Synonym of *Glebionis coronaria* (L.) Cass. ex Spach.
Chrysanthemum myconis	Compositae	Synonym of *Coleostephus myconis* (L.) Rchb.f.
Chrysanthemum pacificum	Compositae	Synonym of *Dendranthema pacificum* (Nakai) Kitam.
Chrysanthemum segetum	Compositae	Synonym of *Glebionis segetum* (L.) Fourr.
Chrysanthemum weyrichii	Compositae	Unresolved
Chrysocephalum apiculatum (Labill.) Steetz.	Compositae	Accepted
Chrysocephalum semipapposum (Labill.) Steetz.	Compositae	Accepted
Chrysothemis pulchella (Donn ex Sims) Decne.	Gesneriaceae	Accepted
Chrysopogon zizanioides (L.) Roberty.	Poaceae	Accepted
Chrysopsis villosa	Compositae	Synonym of *Heterotheca villosa* (Pursh) Shinners.
Cicer arietinum L.	Legumnosae	Accepted
Cichorium endívia L.	Compositae	Accepted
Cichorium intybus L.	Compositae	Accepted
Cichorium pumilum Jacq.	Compositae	Accepted
Cirsium arvense (L.) Scop.	Compositae	Accepted
Cirsium helenioides (L.) Hill	Compositae	Accepted
Cirsium horridulum Michx.	Compositae	Accepted
Cirsium vulgare (Savi) Ten.	Compositae	Accepted
Cissus nodosa Blume	Vitaceae	Accepted
Cistus creticus L.	Cistaceae	Accepted

Cistus salviifolius L.	Cistaceae	Accepted
Citrus hystrix DC.	Rutaceae	Accepted
Citrus × latifolia (Yu.Tanaka) Yu.Tanaka	Rutaceae	Accepted
Citrus limon (L.) Osbeck	Rutaceae	Accepted
Citrus sinensis (L.) Osbeck	Rutaceae	Accepted
Cladanthus mixtus (L.) Oberpr. & Vogt.	Compositae	Accepted
Clematis columbiana var. tenuiloba (A.Gray) J.S.Pringle.	Ranunculaceae	Accepted
Clinopodium acinos (L.) Kuntze.	Lamiaceae	Accepted
Clinopodium alpinum (L.) Kuntze.	Lamiaceae	Accepted
Clinopodium chinense (Benth.) Kuntze.	Lamiaceae	Accepted
Clinopodium douglasii	Lamiaceae	Synonym of *Micromeria douglasii* Benth.
Clinopodium nepeta (L.) Kuntze.	Lamiaceae	Accepted
Clinopodium vulgare L.	Lamiaceae	Accepted
Codiaeum variegatum (L.) Rumph. ex A.Juss.	Euphorbiaceae	Accepted
Codiaeum variegatum var. pictum	Euphorbiaceae	Synonym of Codiaeum variegatum (L.) Rumph. ex A.Juss.
Colchicum autumnale L.	Colchicaceae	Accepted
Collinsia grandiflora Douglas ex Lindl.	Plantaginaceae	Accepted
Comandra umbellata (L.) Nutt.	Santalaceae	Accepted
Commelina communis L.	Commelinaceae	Accepted
Commelina erecta L.	Commelinaceae	Accepted
Complaya trilobata	Compositae	Synonym of *Sphagneticola trilobata* (L.) Pruski.
Conostylis candicans Endl.	Haemodoraceae	Accepted

Consolida regalis Gray	Ranunculaceae	Accepted
Convolvulus angustissimus R. Br.	Convolvulaceae	Accepted
Convolvulus arvensis L.	Convolvulaceae	Accepted
Convolvulus cantabricus	Convolvulaceae	Unresolved
Convolvulus cneorum	Convolvulaceae	Unresolved
Convolvulus mauritanicus	Convolvulaceae	Unresolved
Convolvulus sabatius Viv.	Convolvulaceae	Unresolved
Convolvulus tricolor L.	Convolvulaceae	Accepted
Conyza blakei (Cabrera) Cabrera	Compositae	Synonym of *Erigeron blakei* Cabrera
Conyza bonariensis	Compositae	Synonym of *Erigeron bonariensis* L.
Conyza canadensis	Compositae	Synonym of *Erigeron canadensis* L.
Conyza sumatrensis (S.F.Blake) Pruski & G.Sancho.	Compositae	Accepted
Coprosma acerosa A.Cunn.	Rubiaceae	Accepted
Cordyline fruticosa (L.) A.Chev.	Asparagaceae	Accepted
Cordyline fruticosa ´Firebrand'	Asparagaceae	Variety of *Cordyline fruticosa* (L.) A.Chev.
Coreopsis auriculata L.	Compositae	Accepted
Coreopsis grandiflora Hogg ex Sweet	Compositae	Accepted
Coreopsis lanceolata L.	Compositae	Accepted
Coreopsis palmata Nutt.	Compositae	Accepted
Coreopsis rosea Nutt.	Compositae	Accepted
Coreopsis tinctoria Nutt.	Compositae	Accepted
Corethrogyne filaginifolia (Hook. & Arn.) Nutt.	Compositae	Accepted
Coriandrum sativum L.	Apiaceae	Accepted
Coris monspeliensis L.	Primulaceae	Accepted
Cornus florida L.	Cornaceae	Accepted
Cornus mas L.	Cornaceae	Accepted

Coronilla varia	Leguminosae	Synonym of *Securigera varia* (L.) Lassen.
Correa alba Andrews	Rutaceae	Accepted
Correa glabra Lindl.	Rutaceae	Accepted
Correa reflexa (Labill.) Vent.	Rutaceae	Accepted
Corylus avellana L.	Betulaceae	Accepted
Corynephorus canescens (L.) P.Beauv**.**	Poaceae	Accepted
Cosmos bipinnatus Cav.	Compositae	Accepted
Cota tinctoria (L.) J.Gay	Compositae	Accepted
Cotinus coggigrya Scop.	Anacardiaceae	Accepted
Cotoneaster franchetii Bois.	Rosaceae	Accepted
Cotula hispida (DC.) Harv.	Compositae	Accepted
Cotula squalida (Hook.f.) Hook.f.	Compositae	Accepted
Cotyledon orbiculata L.	Crassulaceae	Accepted
Craspedia variabilis J.Everett & Doust	Compositae	Accepted
Crassula helmsii (Kirk) Cockayne	Crassulaceae	Accepted
Crassula multicava Lem.	Crassulaceae	Accepted
Crassula muscosa L.	Crassulaceae	Accepted
Crassula ovata (Mill.) Druce.	Crassulaceae	Accepted
Crassula tillaea Lest.-Garl.	Crassulaceae	Accepted
Crassula falcata J.C.Wendl.	Crassulaceae	Synonym of *Crassula perfoliata var. falcata* (J.C.Wendl.) Toelken
Crataegus laevigata (Poir.) DC.	Rosaceae	Accepted
Crataegus monogyna Jacq.	Rosaceae	Accepted
Crepidiastrum keiskeanum (Maxim.)	Compositae	Accepted
Crepis bursifolia L.	Compositae	Accepted
Crepis capillaris (L.) Wallr.	Compositae	Accepted
Crepis foetida ssp. rhoeadifolia (M.Bieb.) Čelak.	Compositae	Accepted
Crepis tectorum L.	Compositae	Accepted

Crithmum maritimum	Apiaceae	Unresolved
Crocus minimus DC.	Iridaceae	Accepted
Crocus sativus L.	Iridaceae	Accepted
Crocus sieberi J.Gay.	Iridaceae	Accepted
Crocus tommasinianus Herb.	Iridaceae	Accepted
Crocus vernus (L.) Hill	Iridaceae	Accepted
Crossostephium chinense (A.Gray ex L.) Makino.	Compositae	Accepted
Crotalaria verrucosa L.	Leguminosae	Accepted
Chrysanthellum indicum DC.	Compositae	Accepted
Chrysanthemum morifolium Ramat.	Compositae	Accepted
Cucumis sativus L.	Cucurbitaceae	Accepted
Cucurbita maxima Duchesne	Cucurbitaceae	Accepted
Cullen tenax (Lindl.) J.W.Grimes	Leguminosae	Unresolved
Cuphea hyssopifolia Kunth	Lythraceae	Accepted
Cuphea viscosíssima Jacq.	Lythraceae	Accepted
Curcuma longa L.	Zingiberaceae	Accepted
Cyanotis obtusa (Trimen) Trimen	Commelinaceae	Accepted
Cyanus segetum Hill.	Compositae	Accepted
Cyathula prostrata (L.) Blume.	Amaranthaceae	Accepted
Cycas revoluta Thunb.	Cycadaceae	Accepted
Cyclanthera pedata (L.) Schrad.	Cucurbitacea	Accepted
Cymbopogon ambiguus (Hack.) A.Camus	Poaceae	Accepted
Cymbopogon citratus (DC.) Stapf.	Poaceae	Accepted
Cymbopogon parkeri	Poaceae	Synonym of *Cymbopogon commutatus* (Steud.) Stapf.
Cynara cardunculus L.	Compositae	Accepted
Cynara cardunculus var. scolymus (L.) Benth.	Compositae	Synonym of *Cynara cardunculus* L.
Cynodon dactylon (L.) Pers.	Poaceae	Accepted

Cynodon dactylon x C. transvaalensis Burtt Davy.	Poaceae	Hybrid
Cynosurus cristatus L.	Poaceae	Accepted
Cyperus aggregatus (Willd.) Endl.	Cyperaceae	Accepted
Cyperus alternifolius L.	Cyperaceae	Accepted
Cyperus compressus L.	Cyperaceae	Accepted
Cyperus difformis L.	Cyperaceae	Accepted
Cyperus haspan L.	Cyperaceae	Accepted
Cyperus iria L.	Cyperaceae	Accepted
Cyperus javanicus Houtt.	Cyperaceae	Accepted
Cyperus lupulinus (Spreng.) Marcks.	Cyperaceae	Accepted
Cyperus rotundus L.	Cyperaceae	Accepted
Cyperus strigosus L.	Cyperaceae	Accepted
Cyrtomium falcatum subsp. littorale	Dryopteridaceae	No results
Cystopteris fragilis (L.) Bernh.	Cystopteridaceae	Accepted
Cytisus purgans (L.) Spach.	Leguminosae	Accepted
Dactylis glomerata L.	Poaceae	Accepted
Dactylis polygama Horv.	Poaceae	Synonym of *Dactylis glomerata* L.
Dactylorhiza majalis (Rchb.) P.F.Hunt & Summerh.	Orchidaceae	Accpeted
Dalbergia balansae Prain.	Leguminosae	Accepted
Dalea candida Willd.	Leguminosae	Accepted
Dalea greggii A.Gray.	Leguminosae	Accepted
Dalea purpurea Vent.	Leguminosae	Accepted
Dalea villosa (Nutt.) Spreng.	Leguminosae	Accepted
Dampiera diversifolia de Vriese.	Goodeniaceae	Accepted
Dampiera teres Lindl.	Goodeniaceae	Accepted
Danthonia spicata (L.) Roem. & Schult.	Poaceae	Accepted
Darwinia citriodora (Endl.) Benth.	Myrtaceae	Accepted
Darwinia meeboldii C.A.Gardner	Myrtaceae	Accepted

Darwinia taxifolia subsp. macrolaena B.G.Briggs	Myrtaceae	Synonym of *Darwinia taxifolia* A.Cunn.
Daucus broteroi Ten.	Apiaceae	Accepted
Daucus carota L.	Apiaceae	Accepted
Daucus carota subsp. sativus (Hoffm.) Arcang.	Apiaceae	Accepted
Daviesia ulicifolia C.R.P.Andrews.	Leguminosae	Accepted
Delosperma ashtonii L.Bolus.	Aizoaceae	Accepted
Delosperma basuticum L.Bolus	Aizoaceae	Accepted
Delosperma cooperi (Hook.f.) L.Bolus.	Aizoaceae	Accepted
Delosperma cooperi 'Kelaidis'	Aizoaceae	Cultivar of *Delosperma cooperi* (Hook.f.) L.Bolus.
Delosperma cooperi 'Tiffindell Magenta'	Aizoaceae	Cultivar of *Delosperma cooperi* (Hook.f.) L.Bolus.
Delosperma dyeri L.Bolus	Aizoaceae	Accepted
Delosperma ecklonis var. latifolia	Aizoaceae	No results
Delosperma nubigenum (Schltr.) L.Bolus.	Aizoaceae	Accepted
Delosperma ousberg	Aizoaceae	No results
Delosperma sutherlandii (Hook.f.) N.E.Br.	Aizoaceae	Accepted
Delphinium virescens	Ranunculaceae	Synonym of *Delphinium carolinianum subsp. virescens* (Nutt.) R.E.Brooks.
Derwentia derwentiana	Plantaginaceae	Synonym of *Veronica derwentiana* Andrews.
Derwentia perfoliata	Plantaginaceae	Synonym of *Veronica perfoliata* R.Br.
Deschampsia cespitosa (L.) P.Beauv.	Poaceae	Accepted

Deschampsia flexuosa (L.) Trin.	Poaceae	Accepted
Deschampsia holciformus	Poaceae	Synonym of *Deschampsia cespitosa subsp. holciformis* (J.Presl) W.E.Lawr.
Desmanthus illinoensis (Michx.) MacMill.	Leguminosae	Accepted
Desmodium canadense (L.) DC.	Leguminosae	Accepted
Desmodium gangeticum (L.) DC.	Leguminosae	Accepted
Desmodium marilandicum Darl.	Leguminosae	Accepted
Desmodium paniculatum (L.) DC.	Leguminosae	Accepted
Desmodium triflorum (L.) DC.	Leguminosae	Accepted
Deyeuxia quadriseta (Labill.) Benth.	Poaceae	Synonym of *Calamagrostis quadriseta* (Labill.) Spreng.
Dianella admixta	Xanthorrhoeaceae	Synonym of *Dianella revoluta* R.Br.
Dianella caerula 'Breeze Sims.	Xanthorrhoeaceae	Accepted
Dianella ensifolia (L.) DC.	Xanthorrhoeaceae	Accepted
Dianella ensifolia cv. 'Silvery Stripe	Xanthorrhoeaceae	No results
Dianella revoluta R.Br.	Xanthorrhoeaceae	Accepted
Dianthus anatolicus Boiss.	Caryophyllaceae	Accepted
Dianthus arenarius L.	Caryophyllaceae	Accepted
Dianthus armeria L.	Caryophyllaceae	Accepted
Dianthus carthusianorum L.	Caryophyllaceae	Accepted
Dianthus deltoides L.	Caryophyllaceae	Accepted
Dianthus fruticosus sub. fruticosus	Caryophyllaceae	No results
Dianthus gratianopolitanus Vill.	Caryophyllaceae	Accepted
Dianthus hyssopifolius L.	Caryophyllaceae	Accepted

Dianthus pavonius Tausch	Caryophyllaceae	Accepted
Dianthus superbus L.	Caryophyllaceae	Accepted
Dianthus sylvestris Wulfen.	Caryophyllaceae	Accepted
Dichanthelium acuminatum	Poaceae	Synonym of *Panicum acuminatum* Sw.
Dichanthelium clandestinum	Poaceae	Synonym of *Panicum clandestinum* L.
Dichanthelium dichotomum	Poaceae	Synonym of *Panicum dichotomum* L.
Dichanthelium oligosanthes var. scribnerianum (Nash) Gould	Poaceae	Synonym of *Panicum oligosanthes* Schult.
Dichelachne crinita (L.f.) Hook.f.	Poaceae	Accepted
Dichondra argentea Humb. & Bonpl. ex Willd.	Convolvulaceae	Accepted
Dichondra repens J.R. Forst. & G. Forst.	Convolvulaceae	Accepted
Dicliptera squarrosa Nees	Acanthaceae	Accepted
Dichondra sericea Sw.	Convolvulaceae	Accepted
Dicranoweisia cirrata (Hedw.) Lindb.	Dicranaceae	Accepted
Dietes iridioides (L.) Sweet ex Klatt	Iridaceae	Accepted
Digitaria ciliaris (Retz.) Koeler	Poaceae	Accepted
Digitaria sanguinalis (L.) Scop.	Poaceae	Accepted
Digitaria setigera Roth.	Poaceae	Accepted
Dillwynia sericea A.Cunn.	Leguminosae	Accepted
Dimeria ornithopoda Trin.	Poaceae	Accepted
Diplolaena grandiflora Desf.	Rutaceae	Accepted
Diplotaxis tenuifolia (L.) DC.	Brassicaceae	Accepted
Disphyma australe (Sol. ex Aiton) J.M.Black.	Aizoaceae	Accepted
Disphyma crassifolium (L.) L.Bolus.	Aizoaceae	Accepted

Disphyma crassifolium subsp. Clavellatum	Aizoaceae	Synonym of *Disphyma clavellatum* (Haw.) Chinnock.
Dysphania carinata (R.Br.) Mosyakin & Clemants	Amaranthaceae	Accepted
Dittrichia viscosa (L.) Greuter.	Compositae	Accepted
Dodecatheon media L.	Primulaceae	Accepted
Dodonaea stenozyga F.Muell.	Sapindaceae	Accepted
Draba aizoides L.	Brassicaceae	Accepted
Draba hispanica Boiss.	Brassicaceae	Accepted
Draba nemorosa L.	Brassicaceae	Accepted
Draba streptocarpa A.Gray.	Brassicaceae	Accepted
Dracaena marginata 'bicolor'	Asparagaceae	Variety of *Dracaena marginata* hort.
Dracaena marginata 'colorama'	Asparagaceae	Variety of *Dracaena marginata* hort.
Dracaena reflexa Lam.	Asparagaceae	Accepted
Drosanthemum floribundum (Haw.) Schwantes.	Aizoaceae	Accepted
Drosanthemum hispidum (L.) Schwantes.	Aizoaceae	Accepted
Dryas octopetala subsp. hookeriana (Juz.) Hult,n.	Rosaceae	Accepted
Dudleya caespitosa (Haw.) Britton & Rose.	Crassulaceae	Accepted
Dudleya cymosa (Lem.) Britton & Rose.	Crassulaceae	Accepted
Dudleya farinosa (Lindl.) Britton & Rose.	Crassulaceae	Accepted
Dudleya lanceolata (Nutt.) Britton & Rose.	Crassulaceae	Accepted
Dudleya pulverulenta (Nutt.) Britton & Rose.	Crassulaceae	Accepted

Duranta erecta L.	Verbenaceae	Accepted
Duranta repens	Verbenaceae	Synonym of *Duranta erecta* L.
Dymondia margaretae Compton.	Compositae	Accepted
Ebenus cretica L.	Leguminosae	Accepted
Echeveria affinis E.Walther	Crassulaceae	Accepted
Echeveria atropurpurea (Baker) E.Morren.	Crassulaceae	Accepted
Echeveria ballsii E.Walther	Crassulaceae	Accepted
Echeveria bicolor (Kunth) E.Walther	Crassulaceae	Accepted
Echeveria carnicolor (Baker) E.Morren.	Crassulaceae	Accepted
Echeveria chihuahuaensis Poelln.	Crassulaceae	Accepted
Echeveria cv. hanatsukiyo	Crassulaceae	No results
Echeveria gibbiflora DC.	Crassulaceae	Accepted
Echeveria prolifica Moran & J.Meyran.	Crassulaceae	Accepted
Echeveria pulidonis E.Walther.	Crassulaceae	Accepted
Echeveria quitensis (Kunth) Lindl.	Crassulaceae	Accepted
Echinacea pallida (Nutt.) Nutt.	Compositae	Accepted
Echinacea pupurea (L.) Moench.	Compositae	Accepted
Echinacea tennesseensis (Beadle) Small.	Compositae	Accepted
Echinocereus fendleri (Engelm.) Rümpler.	Cactaceae	Accepted
Echinocereus triglochidiatus Engelm.	Cactaceae	Accepted
Echinocereus viridiflorus Engelm.	Cactaceae	Accepted
Echinochloa colona (L.) Link	Poaceae	Accepted
Echinochloa crus-galli (L.) P.Beauv.	Poaceae	Accepted
Echium amoenum Fisch. & C.A.Mey.	Boraginaceae	Accepted
Echium judaeum Lacaita.	Boraginaceae	Accepted

Echium plantagineum 'Blue Bedder'	Boraginaceae	Variety of *Echium plantagineum* L.
Echium vulgare L.	Boraginaceae	Accepted
Eclipta prostrata (L.) L.	Compositae	Accepted
Einadia hastata (R.Br.) A.J.Scott	Amaranthaceae	Acccepted
Einadia nutans (R.Br.) A.J.Scott	Amaranthaceae	Acccepted
Egeria densa Planch.	Hydrocharitaceae	Accepted
Eleocharis dulcis (Burm.f.) Trin. ex Hensch.	Cyperaceae	Accepted
Eleusine indica (L.) Gaertn.	Poaceae	Accepted
Elymus canadensis L.	Poaceae	Accepted
Elymus hystrix	Poaceae	Synonym of *Hystrix patula* Moench.
Elymus repens (L.) Gould.	Poaceae	Accepted
Elymus virginicus L.	Poaceae	Accepted
Elytrigia repens	Poaceae	Synonym of *Elymus repens* (L.) Gould.
Emerus major	Leguminosae	Unresolved
Emilia fosbergii Nicolson	Compositae	Accepted
Emilia sonchifolia (L.) DC. ex DC.	Compositae	Accepted
Empetrum eamsii Fernald & Wiegand.	Ericaceae	Accepted
Empetrum nigrum L.	Ericaceae	Accepted
Encelia farinosa A.Gray ex Torr.	Compositae	Accepted
Enchylaena tomentosa R.Br.	Amaranthaceae	Accepted
Epacris impressa Labill.	Ericaceae	Accepted
Epacris longiflora Cav.	Ericaceae	Accepted
Ephedra minuta Florin.	Ephedraceae	Accepted
Epilobium adenocaulon	Onagraceae	Synonym of *Epilobium ciliatum* Raf.
Epilobium angustifolium L.	Onagraceae	Accepted

Epilobium canum ssp. canum	Onagraceae	Synonym of *Epilobium canum* (Greene) P.H.Raven.
Epilobium ciliatum Raf.	Onagraceae	Accepted
Epilobium coloratum Biehler.	Onagraceae	Accepted
Epilobium hirsutum L.	Onagraceae	Accepted
Epilobium montanum L.	Onagraceae	Accepted
Epilobium parviflorum Schreb.	Onagraceae	Accepted
Epilobium tetragonum L.	Onagraceae	Accepted
Episcia cupreata (Hook.) Hanst.	Gesneriaceae	Accepted
Equisetum arvense L.	Equisetaceae	Accepted
Equisetum hyemale L.	Equisetaceae	Accepted
Eragrostis atrovirens (Desf.) Trin. ex Steud.	Poaceae	Accepted
Eragrostis barrileri Daveau.	Poaceae	Accepted
Eragrostis cilianensis (All.) Janch.	Poaceae	Accepted
Eragrostis minor Host.	Poaceae	Accepted
Eragrostis pastoensis (Kunth) Trin.	Poaceae	Accepted
Eragrostis pectinacea (Michx.) Nees.	Poaceae	Accepted
Eragrostis retinens Hack. & Arechav.	Poaceae	Accepted
Eragrostis spectabilis (Pursh) Steud.	Poaceae	Accepted
Eragrostis tenella	Poaceae	Synonym of *Eragrostis amabilis* (L.) Wight & Arn.
Eragrostis virescens J.Presl	Poaceae	Accepted
Eremochloa ophiuroides (Munro) Hack.	Poaceae	Accepted
Eremophila divaricata (F.Muell.) F.Muell.	Scrophulariaceae	Accepted
Eremophila gibbifolia (F.Muell.) F.Muell.	Scrophulariaceae	Accepted

Eremophila polyclada (F.Muell.) F.Muell.	Scrophulariaceae	Accepted
Eremophila glabra 'Murchison Magic'	Scrophulariaceae	Variety of *Eremophila glabra* (R.Br.) Ostenf.
Eremophila nivea Chinnock	Scrophulariaceae	Accepted
Ericameria nauseosa ssp. nauseosa var. nauseosa	Compositae	Synonym of *Ericameria nauseosa* (Pall. ex Pursh) G.L.Nesom & G.I.Baird
Erigeron acer	Compositae	Unresolved
Erigeron aureus Greene	Compositae	Accepted
Erigeron acris L.	Compositae	Accepted
Erigeron annuus (L.) Pers.	Compositae	Accepted
Erigeron caespitosus Nutt.	Compositae	Accepted
Erigeron canadensis L.	Compositae	Accepted
Erigeron elatior (A.Gray) Greene.	Compositae	Accepted
Erigeron glaucus Ker Gawl.	Compositae	Accepted
Erigeron karvinskianus	Compositae	Unresolved
Erigeron leiomerus A.Gray.	Compositae	Accepted
Erigeron strigosus Muhl. ex Willd.	Compositae	Accepted
Erigeron vetensis Rydb.	Compositae	Accepted
Eriogonum grande var. rubescens (Greene) Munz.	Polygonaceae	Accepted
Eriogonum latifolium Sm.	Polygonaceae	Accepted
Eriogonum parvifolium Sm.	Polygonaceae	Accepted
Eriogonum umbellatum Torr.	Polygonaceae	Accepted
Eriogonum umbellatum var. aureum (Gand.) Reveal.	Polygonaceae	Accepted
Eriogonum umbellatum var. aureum 'Psdowns'	Polygonaceae	Variey of *Eriogonum umbellatum var. aureum* (Gand.) Reveal.
Eriophyllum lanatum (Pursh) J.Forbes.	Compositae	Accepted
Eriophyllum stoechadifolium Lag.	Compositae	Accepted

Erodium cicutarium (L.) L'Hér.	Geraniaceae	Accepted
Erodium ciliatum	Geraniaceae	No results
Erodium malacoides (L.) L'Hér.	Geraniaceae	Accepted
Erodium manescavii Coss.	Geraniaceae	Accepted
Erophila verna (L.) DC.	Brassicaceae	Accepted
Eruca vesicaria (L.) Cav.	Brassicaceae	Accepted
Eryngium ovinum A.Cunn.	Apiaceae	Accepted
Eryngium yuccifolium Michx.	Apiaceae	Accepted
Erysimum capitatum (Douglas) Greene.	Brassicaceae	Accepted
Erysimum capitatum var. purshii	Brassicaceae	Synonym of *Erysimum capitatum* (Douglas) Greene
Eschscholzia californica 'Maritima'	Papaveraceae	Variety of *Eschscholzia californica* Cham.
Eschscholzia californica Cham.	Papaveraceae	Accepted
Escobaria missouriensis (Sweet) D.R.Hunt.	Cactaceae	Accepted
Escobaria vivípara (Nutt.) Buxb.	Cactaceae	Accepted
Euonymus japonicus Thunb.	Celastraceae	Accepted
Euonymus nanus var. turkestanicus	Celastraceae	Synonym of *Euonymus nanus* M.Bieb.
Eupatorium hyssopifolium L.	Compositae	Accepted
Eupatorium maculatum L.	Compositae	Accepted
Eupatorium perfoliatum L.	Compositae	Accepted
Eupatorium purpureum L.	Compositae	Accepted
Eupatorium serotinum Michx.	Compositae	Accepted
Euphorbia acerensis Boiss.	Euphorbiaceae	Accepted
Euphorbia albomarginata Torr. & A.Gray	Euphorbiaceae	Accepted
Euphorbia antisyphilitica Zucc.	Euphorbiaceae	Accepted
Euphorbia characias L.	Euphorbiaceae	Accepted
Euphorbia corollata L.	Euphorbiaceae	Accepted

Euphorbia cyparissias L.	Euphorbiaceae	Accepted
Euphorbia exígua L.	Euphorbiaceae	Accepted
Euphorbia graminea Jacq.	Euphorbiaceae	Accepted
Euphorbia hirta L.	Euphorbiaceae	Accepted
Euphorbia humifusa Willd.	Euphorbiaceae	Accepted
Euphorbia hypericifolia L.	Euphorbiaceae	Accepted
Euphorbia hyssopifolia L.	Euphorbiaceae	Accepted
Euphorbia jolkinii Boiss.	Euphorbiaceae	Accepted
Euphorbia klotzschii Oudejans	Euphorbiaceae	Accepted
Euphorbia maculata L.	Euphorbiaceae	Accepted
Euphorbia milii Des Moul.	Euphorbiaceae	Accepted
Euphorbia myrsinites L.	Euphorbiaceae	Accepted
Euphorbia peplus L.	Euphorbiaceae	Accepted
Euphorbia pithyusa L.	Euphorbiaceae	Accepted
Euphorbia polychroma 'Candy	Euphorbiaceae	Synonym of *Euphorbia epithymoides* L.
Euphorbia sciadophila Boiss.	Euphorbiaceae	Accepted
Euphorbia segetalis L.	Euphorbiaceae	Accepted
Euphorbia serpens Kunth	Euphorbiaceae	Accepted
Euphorbia stenoclada Baill.	Euphorbiaceae	Accepted
Euphorbia thymifolia L.	Euphorbiaceae	Accepted
Euphorbia veneris M.L.S.Khan.	Euphorbiaceae	Accepted
Euryomyrtus ramosissima (A.Cunn.) Trudgen	Myrtaceae	Accepted
Eustachys distichophylla (Lag.) Nees	Poaceae	Accepted
Eustachys retusa (Lag.) Kunth.	Poaceae	Accepted
Eutaxia microphylla (R.Br.) J.M.Black.	Leguminosae	Accepted
Euthamia graminifolia (L.) Nutt.	Compositae	Accepted
Euthamia tenuifolia (Pursh) Nutt.	Compositae	Synonym of *Euthamia graminifolia* (L.) Nutt.
Evolvulus alsinoides (L.) L.	Convolvulaceae	Accepted

Evolvulus glomeratus Nees & C. Mart.	Convolvulaceae	Accepted
Evolvulus pilosus	Convolvulaceae	Unresolved
Evolvulus nummularius (L.) L.	Convolvulaceae	Accepted
Excoecaria cochinchinensis Lour.	Euphorbiaceae	Accepted
Fagopyrum esculentum Moench.	Polygonaceae	Accepted
Fallopia convolvulus (L.) Á.Löve.	Polygonaceae	Accepted
Fallopia dumetorum (L.) Holub	Polygonaceae	Accepted
Fallopia japonica	Polygonaceae	Synonym of *Reynoutria japonica* Houtt.
Fendlera rupicola var. wrightii (Engelm. & A.Gray) A.Heller.	Hydrangeaceae	Accepted
Festuca amethystina L.	Poaceae	Accepted
Festuca arundinacea Schreb.	Poaceae	Accepted
Festuca brachyphylla Schult. & Schult.f.	Poaceae	Accepted
Festuca brachyphylla ssp. breviculmis 'Pt. Joe'	Poaceae	Synonym of *Festuca brachyphylla* Schult. & Schult.f.
Festuca californica Vasey.	Poaceae	Accepted
Festuca filiformis Pourr.	Poaceae	Accepted
Festuca gigantea (L.) Vill.	Poaceae	Accepted
Festuca glauca Vill.	Poaceae	Accepted
Festuca idahoensis Elmer.	Poaceae	Accepted
Festuca longifolia Thuill.	Poaceae	Accepted
Festuca marginata (Hack.) K.Richt.	Poaceae	Accepted
Festuca ovina L.	Poaceae	Accepted
Festuca ovina subsp. cinerea	Poaceae	No results
Festuca ovina subsp. glauca	Poaceae	Synonym of *Festuca arvernensis* Auquier, Kerguélen & Markgr.-Dann.

Festuca ovina subsp. ovina	Poaceae	Synonym of *Festuca ovina* L.
Festuca ovina var. vulgaris	Poaceae	Synonym of *Festuca ovina* L.
Festuca roemeri	Poaceae	Synonym of *Festuca idahoensis subsp. roemeri* (Pavlick) S.G.Aiken.
Festuca rubra 'Patricks Point'	Poaceae	Synonym of *Festuca rubra* L.
Festuca rubra L.	Poaceae	Accepted
Festuca rubra subsp. arenaria	Poaceae	Synonym of *Festuca rubra* L.
Festuca rubra subsp. fallax	Poaceae	Synonym of *Festuca rubra* L.
Festuca saximontana Rydb.	Poaceae	Accepted
Festuca trachyphylla (Hack.) Hack.	Poaceae	Accepted
Ficinia nodosa (Rottb.) Goetgh., Muasya & D.A.Simpson.	Cyperaceae	Accepted
Ficus carica L.	Moraceae	Accepted
Ficus microcarpa 'golden'	Moraceae	Variety of *Ficus microcarpa* L.f.
Ficus microcarpa L.f.	Moraceae	Accepted
Ficus pumila L.	Moraceae	Accepted
Ficus radicans Desf.	Moraceae	Synonym of *Ficus sagittata* Vahl
Ficus religiosa L.	Moraceae	Accepted
Filago pyramidata L.	Compositae	Accepted
Filipendula purpurea	Rosaceae	Synonym of *Filipendula glaberrima* Nakai.
Filipendula vulgaris 'Kahome'	Rosaceae	Variety of *Filipendula vulgaris* Moench.
Filipendula vulgaris Moench.	Rosaceae	Accepted
Fimbristylis cymosa R.Br.	Cyperaceae	Accepted

Fimbristylis dichotoma (L.) Vahl.	Cyperaceae	Accepted
Fimbristylis ovata (Burm.f.) J.Kern	Cyperaceae	Accepted
Fittonia verschaffeltii (Lem.) Van Houtte	Acanthaceae	Synonym of *Fittonia albivenis* (Lindl. ex Veitch) Brummitt
Foeniculum vulgare Mill.	Apiaceae	Accepted
Fragaria chiloensis (L.) Mill.	Rosaceae	Accepted
Fragaria vesca L.	Rosaceae	Accepted
Fragaria virginiana Mill.	Rosaceae	Accepted
Fragaria x ananassa (Duchesne ex Weston) Duchesne ex Rozier.	Rosaceae	Accepted
Frangula californica 'Eve Case'	Rhamnaceae	Variety of *Frangula californica* (Eschsch.) A.Gray.
Frankenia laevis L.	Frankeniaceae	Accepted
Frankenia thymifolia Desf.	Frankeniaceae	Accepted
Fraxinus excelsior L.	Oleaceae	Accepted
Galeopsis pubescens Besser	Lamiaceae	Accepted
Gaillardia aristata Pursh	Compositae	Accepted
Gaillardia pulchella Foug.	Compositae	Accepted
Galinsoga ciliata Ruiz & Pav.	Compositae	Accepted
Galinsoga parviflora Cav.	Compositae	Accepted
Galium aparine L.	Rubiaceae	Accepted
Galium boreale L.	Rubiaceae	Accepted
Galium lucidum All.	Rubiaceae	Accepted
Galium mollugo L.	Rubiaceae	Accepted
Galium odoratum (L.) Scop.	Rubiaceae	Accepted
Galium pusillum L.	Rubiaceae	Accepted
Galium verum L.	Rubiaceae	Accepted
Galphimia glauca Cav.	Malpighiaceae	Accepted
Gasteria gracilis	Xanthorrhoeaceae	Unresolved
Gaultheria procumbens L.	Ericaceae	Accepted

Gaura lindheimeri Engelm. & A.Gray.	Onagraceae	Accepted
Gazania nivea	Compositae	Synonym of *Gazania jurineifolia* DC.
Gazania rigens (L.) Gaertn.	Compositae	Accepted
Gazania uniflora	Compositae	Synonym of *Gazania rigens* (L.) Gaertn.
Genista pilosa L.	Leguminosae	Accepted
Gentiana andrewsii Griseb.	Gentianaceae	Accepted
Geranium × cantabrigiense P.F.Yeo	Geraniaceae	Unresolved
Geranium cinereum ‘Ballerina’	Geraniaceae	Cutivar of *Geranium cinereum* Cav.
Geranium columbinum L.	Geraniaceae	Accepted
Geranium endressii J.Gay.	Geraniaceae	Accepted
Geranium lucidum L.	Geraniaceae	Accepted
Geranium molle L.	Geraniaceae	Accepted
Geranium purpureum Vill.	Geraniaceae	Accepted
Geranium pusillum L.	Geraniaceae	Accepted
Geranium robertianum L.	Geraniaceae	Accepted
Geranium sanguineum L.	Geraniaceae	Accepted
Geranium zonale L.	Geraniaceae	Synonym of *Pelargonium zonale* (L.) L'Hér. ex Aiton
Geum rivale L.	Rosaceae	Accepted
Geum triflorum Pursh.	Rosaceae	Accepted
Geum urbanum L.	Rosaceae	Accepted
Glandularia x hybrid	Verbenaceae	Unresolved
Glaucium flavum Crantz.	Papaveraceae	Accepted
Glebionis coronarium (L.) Cass. ex Spach.	Compositae	Accepted
Glechoma hederacea L.	Lamiaceae	Accepted
Glechoma longituba (Nakai) Kuprian. (Nakai) Kuprian.	Lamiaceae	Accepted

Glycyrrhiza glabra L.	Leguminosae	Accepted
Gnaphalium antennarioides DC.	Compositae	Accepted
Gnaphalium pensylvanicum Willd.	Compositae	Accepted
Gomphrena globosa L.	Amaranthaceae	Accepted
Gomphrena pulchella Mart.	Amaranthaceae	Accepted
Gomphocarpus fruticosus (L.) W.T.Aiton	Apocynaceae	Accepted
Goodenia benthamiana Carolin.	Goodeniaceae	Accepted
Goodenia pinnatifida Schltdl.	Goodeniaceae	Accepted
Goodenia varia R.Br.	Goodeniaceae	Accepted
Glandularia tenera (Spreng.) Cabrera	Verbenaceae	Accepted
Grahamia bracteata Gillies ex Hook. & Arn.	Anacampserotaceae	Accepted
Graptopetalum paraguayense (N.E.Br.) E.Walther.	Crassulaceae	Accepted
Graptopetalum paraguayense subsp. rose	Crassulaceae	No results
Grevillea alpina Lindl.	Proteaceae	Accepted
Grevillea bipinnatifida R.Br.	Proteaceae	Accepted
Grevillea humilis ‘Coastal Gem’	Proteaceae	Variety of *Grevillea humilis* Makinson
Grevillea leptobotrys Meisn.	Proteaceae	Accepted
Grevillea rosmarinifolia A.Cunn.	Proteaceae	Accepted
Grindelia cabrerae Ariza.	Compositae	Accepted
Grindelia hirsutula Hook. & Arn.	Compositae	Accepted
Grindelia stricta	Compositae	Synonym of *Grindelia hirsutula* Hook. & Arn.
Gymnadenia odoratissima (L.) Rich.	Orchidaceae	Accepted
Gypsophila muralis L.	Caryophyllaceae	Accepted
Gypsophila repens ‘Dorothy Teacher’	Caryophyllaceae	Variety of *Gypsophila repens* L.

Gypsophila repens 'Rosea'	Caryophyllaceae	Variey of *Gypsophila repens* L.
Halimione portulacoides	Amaranthaceae	Synonym of *Atriplex portulacoides* L.
Haplopappus macrocephalus (Poepp. ex Less.) DC.	Compositae	Accepted
Hardenbergia violácea (Schneev.) Stearn.	Leguminosae	Accepted
Hedera helix L.	Araliaceae	Accepted
Hedera hibérnica (G.Kirchn.) Carrière.	Araliaceae	Accepted
Hedyotis corymbosa	Rubiaceae	Synonym of *Oldenlandia corymbosa* L.
Hedyotis longifolia	Rubiaceae	Synonym of *Houstonia longifolia* Gaertn.
Hedyotis pinifolia	Rubiaceae	Synonym of *Oldenlandia pinifolia* (Wall. ex G.Don) Kuntze.
Hedyotis strigulosa var. parvifolia	Rubiaceae	Synonym of *Oldenlandia strigulosa* Bartl. ex DC.
Hedyotis verticillata	Rubiaceae	Synonym of *Oldenlandia verticillata* L.
Heimia salicifolia (Kunth) Link	Lythraceae	Accepted
Helenium autumnale L.	Compositae	Accepted
Helianthemum italicum	Cistaceae	Synonym of *Helianthemum oelandicum subsp. italicum* (L.) Font Quer & Rothm.
Helianthemum mummularium (L.) Mill.	Cistaceae	Accepted
Helianthus annuus L.	Compositae	Accepted
Helianthus mollis Lam.	Compositae	Accepted

Helichrysum arenarium (L.) Moench.	Compositae	Accepted
Helichrysum italicum (Roth) G.Don.	Compositae	Accepted
Helichrysum italicum subsp. microphyllum (Willd.) Nyman.	Compositae	Accepted
Helichrysum luteoalbum (L.) Rchb.	Compositae	Accepted
Helichrysum orientale (L.) Vaill.	Compositae	Accepted
Helichrysum rutidolepis DC.	Compositae	Accepted
Helichrysum stoechas (L.) Moench.	Compositae	Accepted
Helichrysum stoechas subsp. barrelieri (Ten.) Nyman	Compositae	Accepted
Heliconia 'American Dwarf'	Heliconiaceae	No results
Helicotrichon sempervirens (Vill.) Pilg.	Poaceae	Accepted
Helictotrichon pratense (L.) Pilg.	Poaceae	Accepted
Heliopsis helianthoides (L.) Sweet.	Compositae	Accepted
Heliotropium curassavicum L.	Boraginaceae	Accepted
Heliotropium europaeum L.	Boraginaceae	Accepted
Heliotropium hirsutissimum Grauer.	Boraginaceae	Accepted
Helleborus foetidus L.	Ranunculaceae	Accepted
Hemerocallis 'Golden Chimes',	Xanthorrhoeaceae	No results
Hemerocallis 'Mary's Gold'	Xanthorrhoeaceae	No results
Hemerocallis 'Stella de Oro'	Xanthorrhoeaceae	No results
Hemerocallis fulva (L.) L.	Xanthorrhoeaceae	Accepted
Hemerocallis fulva var. littorea	Xanthorrhoeaceae	Synonym of *Hemerocallis littorea* Makino.
Hemigraphis alternata (Burm.f.) T.Anderson	Acanthaceae	Accepted
Herniaria glabra L.	Caryophyllaceae	Accepted

Herniaria glabra 'Sea Foam'	Caryophyllaceae	Variety of *Herniaria glabra* L.
Herniaria hirsuta L.	Caryophyllaceae	Accepted
Hesperaloe parviflora (Torr.) J.M.Coult.	Asparagaceae	Accepted
Hesperostipa spartea (Trin.) Barkworth	Poaceae	Synonym of *Stipa spartea* Trin.
Heterocentron elegans (Schltdl.) Kuntze.	Melastomataceae	Accepted
Heterosperma ovatifolium Cav.	Compositae	Accepted
Heterotheca jonesii (S.F.Blake) S.L.Welsh & N.D.Atwood.	Compositae	Accepted
Heuchera 'Electra'	Saxifragaceae	No results
Heuchera 'Obsidian'	Saxifragaceae	No results
Heuchera abramsii Rydb.	Saxifragaceae	Accepted
Heuchera americana L.	Saxifragaceae	Accepted
Heuchera cylindrica Douglas.	Saxifragaceae	Accepted
Heuchera elegans Abrams.	Saxifragaceae	Accepted
Heuchera grossulariifolia	Saxifragaceae	Synonym of *Heuchera hallii var. grossularifolia* (Rydb.) Rosend.
Heuchera maxima Greene.	Saxifragaceae	Accepted
Heuchera micrantha Douglas ex Lindl.	Saxifragaceae	Accepted
Heuchera parishii Rydb.	Saxifragaceae	Accepted
Heuchera parvifolia Nutt. ex Torr. & A. Gray.	Saxifragaceae	Accepted
Heuchera pulchella Wooton & Standl.	Saxifragaceae	Accepted
Heuchera richardsonii R. Br.	Saxifragaceae	Accepted
Hibbertia obtusifolia	Dilleniaceae	Unresolved
Hibiscus sabdariffa L.	Malvaceae	Accepted

Hieracium aurantiacum	Compositae	Synonym of *Pilosella aurantiaca* (L.) F.W.Schultz & Sch.Bip.
Hieracium canadense	Compositae	Synonym of *Hieracium umbellatum* L.
Hieracium murorum C.B.Clarke.	Compositae	Accepted
Hieracium pilosella	Compositae	Synonym of *Pilosella officinarum* Vaill.
Hieracium umbellatum	Compositae	Accepted
Hilaria jamesii (Torr.) Benth.	Poaceae	Accepted
Hippocrepis comosa L.	Leguminosae	Accepted
Hirschfeldia incana (L.) Lagr.-Foss.	Brassicaceae	Accepted
Holcus lanatus L.	Poaceae	Accepted
Hordeum geniculatum	Poaceae	Synonym of *Hordeum marinum subsp. gussoneanum* (Parl.) Thell.
Hordeum glaucum	Poaceae	Synonym of *Hordeum murinum subsp. glaucum* (Steud.) Tzvelev.
Hornungia petraea (L.) Rchb.	Brassicaceae	Accepted
Houstonia caerulea L.	Rubiaceae	Accepted
Humulus lupulus L.	Cannabaceae	Accepted
Hydrocotyle sibthorpioides Lam.	Araliaceae	Accepted
Hydrocotyle verticillata Thunb.	Araliaceae	Accepted
Hylotelephium cyaneum H. Ohba	Crassulaceae	Synonym of *Sedum cyaneum* J. Rudolph
Hylotelephium erythrostictum (Miq.) H. Ohba.	Crassulaceae	Accepted
Hylotelephium ewersii	Crassulaceae	Synonym of *Sedum ewersii* Ledeb.
Hylotelephium maximum	Crassulaceae	Synonym of *Sedum maximum* (L.) Suter.

Hylotelephium spectabile (Boreau) H. Ohba.	Crassulaceae	Accepted
Hypericum calycinum L.	Hypericaceae	Accepted
Hypericum empetrifolium Willd.	Hypericaceae	Accepted
Hylotelephium telephium	Crassulaceae	Synonym of *Sedum telephium* L.
Hypericum moserianum	Hypericaceae	Unresolved
Hypericum monogynum L.	Hypericaceae	Accepted
Hypericum olympicum L.	Hypericaceae	Accepted
Hypericum perforatum L.	Hypericaceae	Accepted
Hypochaeris radicata	Compositae	Unresolved
Hypoxis hirsuta (L.) Coville.	Hypoxidaceae	Accepted
Hyssopus officinalis L.	Lamiaceae	Accepted
Hysterionica jasionoides Willd.	Compositae	Accepted
Hystrix patula Moench.	Poaceae	Accepted
Iberis amara L.	Brassicaceae	Accepted
Iberis sempervirens L.	Brassicaceae	Accepted
Iberis umbellata 'Fairy'	Brassicaceae	Variety of *Iberis umbellata* L.
Ilex cornuta Lindl. & Paxton.	Aquifoliaceae	Accepted
Illecebrum verticillatum L.	Caryophyllaceae	Accepted
Illicium verum Hook.f.	Schisandraceae	Accepted
Impatiens noli-tangere L.	Balsaminaceae	Accepted
Impatiens parviflora DC.	Balsaminaceae	Accepted
Impatiens walleriana Hook.f.	Balsaminaceae	Accepted
Imperata cylindrica (L.) Raeusch.	Poaceae	Accepted
Imperata cylindrica var. major	Poaceae	Synonym of *Imperata cylindrica* (L.) Raeusch.
Indigofera australis Willd.	Leguminosae	Accepted
Inula britannica L.	Compositae	Accepted
Inula conyza (Griess.) DC.	Compositae	Accepted
Inula hirta L.	Compositae	Accepted
Ionactis linariifolia (L.) Greene.	Compositae	Accepted

Ipheion uniflorum Raf.	Amaryllidaceae	Accepted
Ipomoea batatas (L.) Lam.	Convolvulaceae	Accepted
Ipomoea cairica (L.) Sweet	Convolvulaceae	Accepted
Ipomoea nil (L.) Roth	Convolvulaceae	Accepted
Ipomoea pes-caprae (L.) R. Br.	Convolvulaceae	Accepted
Ipomoea purpurea (L.) Roth	Convolvulaceae	Accepted
Ipomoea quamoclit L.	Convolvulaceae	Accepted
Ipomoea triloba L.	Convolvulaceae	Accepted
Ipomopsis aggregata (Pursh) V.E. Grant.	Polemoniaceae	Accepted
Ipomopsis rubra (L.) Wherry.	Polemoniaceae	Accepted
Iris 'Music Box'	Iridaceae	No results
Iris bucharica Foster.	Iridaceae	Accepted
Iris chamaeiris	Iridaceae	Synonym of *Iris lutescens* Lam.
Iris chrysophylla Howell.	Iridaceae	Accepted
Iris danfordiae (Baker) Boiss.	Iridaceae	Accepted
Iris douglasiana Herb.	Iridaceae	Accepted
Iris germanica dwarf	Iridaceae	Variety of *Iris* × *germanica* L.
Iris lutescens Lam.	Iridaceae	Accepted
Iris pumila L.	Iridaceae	Accepted
Iris reticulata M.Bieb.	Iridaceae	Accepted
Iris sibirica L.	Iridaceae	Accepted
Iris tectorum Maxim.	Iridaceae	Accepted
Isotoma axillaris Lindl.	Campanulaceae	Accepted
Ixiolirion pallasii	Ixioliriaceae	Synonym of *Ixiolirion tataricum* (Pall.) Schult. & Schult.f.
Ixora chinensis Lam.	Rubiaceae	Accepted
Ixora coccinea L.	Rubiaceae	Accepted
Ixora williamsii	Rubiaceae	Synonym of *Ixora casei* Hance.

Jacobaea maritima (L.) Pelser & Meijden	Compositae	Accepted
Jasione montana L.	Campanulaceae	Accepted
Juncus balticus Willd.	Juncaceae	Accepted
Juncus patens E.Mey.	Juncaceae	Accepted
Juncus secundus P.Beauv. ex Poir.	Juncaceae	Accepted
Juncus tenuis Willd.	Juncaceae	Accepted
Juncus xiphioides E.Mey.	Juncaceae	Accepted
Juniperus communis L.	Cupressaceae	Accepted
Juniperus horizontalis Moench	Cupressaceae	Accepted
Justicia squarrosa Griseb.	Acanthaceae	Accepted
Kalanchoe blossfeldiana Poelln.	Crassulaceae	Accepted
Kalanchoe daigremontiana Raym.-Hamet & H. Perrier	Crassulaceae	Accepted
Kalanchoe fedtschenkoi	Crassulaceae	Synonym of *Bryophyllum fedtschenkoi* (Raym.-Hamet & H.Perrier) Lauz.-March.
Kalanchoe garambiensis Kudô.	Crassulaceae	Accepted
Kalanchoe hildebrandtii Baill.	Crassulaceae	Accepted
Kalanchoe longiflora var. coccinea	Crassulaceae	No results
Kalanchoe milloti Raym.-Hamet & H. Perrier.	Crassulaceae	Accepted
Kalanchoe thyrsiflora	Crassulaceae	Synonym of *Kalanchoe tetraphylla* H. Perrier.
Kalanchoe tomentosa Baker	Crassulaceae	Accepted
Kalanchoe × houghtonii D.B.Ward	Crassulaceae	Unresolved
Kalmia polifolia Wangenh.	Ericaceae	Accepted
Kleinia mandraliscae Tineo.	Compositae	Accepted
Knautia arvensis (L.) Coult.	Caprifoliaceae	Accepted
Kniphofia uvaria (L.) Oken	Xanthorrhoeaceae	Accepted
Koeleria cristata	Poaceae	Unresolved

Koeleria glauca (Spreng.) DC.	Poaceae	Accepted
Koeleria macrantha (Ledeb.) Schult.	Poaceae	Accepted
Koeleria pyramidata (Lam.) P.Beauv.	Poaceae	Accepted
Koeleria vallesiana (Honck.) Bertol. ex Schult.	Poaceae	Accepted
Kuhnia eupatorioides	Compositae	Synonym of *Brickellia eupatorioides* (L.) Shinners.
Kyllinga brevifolia Rottb.	Cyperaceae	Accepted
Lactuca perennis L.	Compositae	Accepted
Lactuca quercina L.	Compositae	Accepted
Lactuca sativa L.	Compositae	Accepted
Lactuca sativa subsp. crispa (L.) Schübl. & G.Martens	Compositae	Synonym of *Lactuca sativa* L.
Lactuca sativa var. longifolia	Compositae	Synonym of *Lactuca sativa* L.
Lactuca serriola L.	Compositae	Accepted
Laennecia sophiifolia (Kunth) G.L.Nesom	Compositae	Accepted
Lagerstroemia indica L.	Lythraceae	Accepted
Lagurus ovatus L.	Poaceae	Accepted
Lamium album L.	Lamiaceae	Accepted
Lampranthus blandus Schwantes.	Aizoaceae	Accepted
Lampranthus deltoides (L.) Glen ex Wijnands.	Aizoaceae	Accepted
Lampranthus multiradiatus (Jacq.) N.E.Br.	Aizoaceae	Accepted
Lampranthus spectabilis (Haw.) N.E. Br.	Aizoaceae	Accepted
Lantana camara L.	Verbenaceae	Accepted
Lapsana communis L.	Compositae	Accepted
Lasthenia california	Compositae	Unresolved

Lathyrus maritima	Leguminosae	Synonym of *Lathyrus japonicus subsp. maritimus* (L.) P.W.Ball
Lathyrus pratensis L.	Leguminosae	Accepted
Laurus nobilis L.	Lauraceae	Accepted
Lavandula angustifolia Mill.	Lamiaceae	Accepted
Lavandula dentata L.	Lamiaceae	Accepted
Lavandula dentata var. dentata	Lamiaceae	Synonym of *Lavandula dentata* L.
Lavandula dentata var. candicans Batt.	Lamiaceae	Accepted
Lavandula angustifolia Mill.	Lamiaceae	Accepted
Lavandula spica L.	Lamiaceae	Synonym of *Lavandula angustifolia* Mill.
Lavandula stoechas L.	Lamiaceae	Accepted
Lavandula stoechas subsp. luisieri (Rozeira) Rozeira.	Lamiaceae	Accepted
Lavatera cretica	Malvaceae	Synonym of *Malva multiflora* (Cav.) Soldano, Banfi & Galasso.
Layia platyglossa (Fisch. & C.A.Mey.) A.Gray.	Compositae	Accepted
Lechea minor L.	Cistaceae	Accepted
Lens culinaris Medik	Leguminosae	Accepted
Leontodon autumnalis	Compositae	Synonym of *Scorzoneroides autumnalis* (L.) Moench.
Leontodon hispidus L.	Compositae	Accepted
Leontodon saxatilis Lam.	Compositae	Accepted
Leontodon tuberosus L.	Compositae	Accepted
Leonurus japonicus Houtt.	Lamiaceae	Accepted
Leopoldia comosa (L.) Parl.	Asparagaceae	Accepted
Lepidosperma concavum R.Br.	Cyperaceae	Accepted

Lepironia articulata (Retz.) Domin.	Cyperaceae	Accepted
Leptinella squalida Hook.f.	Compositae	Accepted
Leptochloa crinita	Poaceae	Accepted
Leptorhynchos tenuifolius F.Muell.	Compositae	Accepted
Leptorhynchos squamatus (Labill.) Less.	Compositae	Accepted
Leptospermum rotundifolium (Maiden & Betche) F.A.Rodway	Myrtaceae	Accepted
Lespedeza capitata Michx.	Leguminosae	Accepted
Lespedeza virginica (L.) Britton	Leguminosae	Accepted
Leucanthemum × superbum (Bergmans ex J.W.Ingram) D.H.Kent.	Compositae	Accepted
Leucanthemum atlanticum	Compositae	Synonym of *Rhodanthemum atlanticum* (Ball) "B.H.Wilcox, K.Bremer & Humphries".
Leucanthemum vulgare (Vaill.) Lam.	Compositae	Accepted
Leucochrysum albicans (A.Cunn.) Paul G.Wilson	Compositae	Accepted
Leucophyllium frutescens (Berland.) I.M. Johnst.	Scrophulariaceae	Accepted
Leymus mollis (Trin.) Pilg.	Poaceae	Accepted
Leymus racemosus (Lam.) Tzvelev	Poaceae	Accepted
Liatris aspera Michx.	Compositae	Accepted
Liatris cylindracea Michx.	Compositae	Accepted
Liatris ligulistylis (A.Nelson) K.Schum.	Compositae	Accepted
Liatris punctata Hook.	Compositae	Accepted
Liatris pycnostachya Michx.	Compositae	Accepted
Liatris spicata (L.) Willd.	Compositae	Accepted
Ligustrum sinense Lour.	Oleaceae	Accepted

Ligustrum vicaryi	Oleaceae	Unresolved
Ligustrum vulgare L.	Oleaceae	Accepted
Lilium pavia	Liliaceae	No results
Lilium philadelphicum L.	Liliaceae	Accepted
Limoniastrum monopetalum (L.) Boiss.	Plumbaginaceae	Accepted
Limonium carolinianum (Walter) Britton.	Plumbaginaceae	Accepted
Limonium graecum (Poir.) Kuntze	Plumbaginaceae	Accepted
Limonium latifolium	Plumbaginaceae	Synonym of *Limonium platyphyllum* Lincz.
Limonium virgatum	Plumbaginaceae	Synonym of *Limonium oleifolium* Mill.
Linaria elegans Cav.	Plantaginaceae	Accepted
Linaria maroccana Hook. f.	Plantaginaceae	Accepted
Linaria vulgaris Mill.	Plantaginaceae	Accepted
Lindernia antípoda (L.) Alston.	Linderniaceae	Accepted
Lindernia crustácea (L.) F.Muell.	Linderniaceae	Accepted
Lindernia rotundifolia (L.) Alston.	Linderniaceae	Accepted
Linum bienne Mill.	Linaceae	Accepted
Linum catharticum L.	Linaceae	Accepted
Linum grandiflorum var. rubrum	Linaceae	No results
Linum intercursum E.P. Bicknell.	Linaceae	Accepted
Linum lewisii Pursh.	Linaceae	Accepted
Linum marginale A.Cunn. ex Planch.	Linaceae	Accepted
Linum perenne L.	Linaceae	Accepted
Linum strictum L.	Linaceae	Accepted
Linum suffruticosum L.	Linaceae	Accepted
Linum tenuifolium L.	Linaceae	Accepted
Linum usitatissimum L.	Linaceae	Accepted
Liriope muscari (Decne.) L.H.Bailey	Asparagaceae	Accepted
Liriope spicata Lour.	Asparagaceae	Accepted
Lobelia cardinalis L.	Campanulaceae	Accepted

Lobelia erinus L.	Campanulaceae	Accepted
Lobelia pratioides Benth.	Campanulaceae	Accepted
Lobelia siphilitica L.	Campanulaceae	Accepted
Lobelia spicata var. spicata	Campanulaceae	Synonym of *Lobelia spicata* Lam.
Lobularia maritima (L.) Desv.	Brassicaceae	Accepted
Lolium multiflorum Lam.	Poaceae	Accepted
Lolium perenne L.	Poaceae	Accepted
Lomandra filiformis (Thunb.) Britten.	Asparagaceae	Accepted
Lomandra longifolia 'Tanika	Asparagaceae	Variety of *Lomandra longifolia* Labill.
Lomandra longifolia Labill.	Asparagaceae	Accepted
Lomandra micrantha (Endl.) Ewart.	Asparagaceae	Accepted
Lomandra multiflora (R.Br.) Britten.	Asparagaceae	Accepted
Lomatium utriculatum (Nutt. ex Torr. & A. Gray) J.M. Coult. & Rose	Apiaceae	Accepted
Lomelosia cretica (L.) Greuter & Burdet	Caprifoliaceae	Unresolved
Lomelosia prolifera	Caprifoliaceae	Unresolved
Lonicera dioica L.	Caprifoliaceae	Accepted
Lonicera hispidula Douglas ex Torr. & A. Gray.	Caprifoliaceae	Accepted
Lonicera pileata	Caprifoliaceae	Synonym of *Lonicera ligustrina var. pileata* (Oliv.) Franch.
Loropetalum chinense (R. Br.) Oliv.	Hamamelidaceae	Accepted
Loropetalum chinense var. rubrum Yieh.	Hamamelidaceae	Accepted
Lotus alpinus (DC.) Ramond.	Leguminosae	Accepted
Lotus corniculatus L.	Leguminosae	Accepted
Lotus corniculatus subsp. corniculatus	Leguminosae	Synonym of *Lotus corniculatus* L.

Lotus creticus L.	Leguminosae	Accepted
Luffa cylindrica (L.) M.Roem.	Cucurbitaceae	Accepted
Lupinus albus L.	Leguminosae	Accepted
Lupinus arboreus Sims.	Leguminosae	Accepted
Lupinus bicolor Lindl.	Leguminosae	Accepted
Lupinus nanus Benth.	Leguminosae	Accepted
Lupinus perennis L.	Leguminosae	Accepted
Lupinus variicolor Steud.	Leguminosae	Accepted
Luzula campestris (L.) DC.	Juncaceae	Accepted
Luzula capitata (Miq. ex Franch. & Sav.) Kom.	Juncaceae	Accepted
Luzula multiflora (Ehrh.) Lej.	Juncaceae	Accepted
Lychnis chalcedonica L.	Caryophyllaceae	Synonym of *Silene chalcedonica* (L.) E.H.L.Krause
Lychnis coronaria var 'Alba'	Caryophyllaceae	Synonym of *Silene coronaria* (Desr.) Clairv. ex Rchb.
Lychnis viscaria	Caryophyllaceae	Synonym of *Silene viscaria* (L.) Jess.
Lycopersicon esculentum Mill.	Solanaceae	Accepted
Lygodium scandens (L.) Sw.	Lygodiaceae	Accepted
Lysimachia linum-stellatum L.	Primulaceae	Accepted
Lysimachia mauritiana Lam.	Primulaceae	Accepted
Lythrum salicaria L.	Lythraceae	Accepted
Macaranga tanarius (L.) Müll.Arg.	Euphorbiaceae	Accepted
Macropidia fuliginosa (Hook.) Druce	Haemodoraceae	Accepted
Macroptilium lathyroides (L.) Urb.	Leguminosae	Accepted
Magnolia grandiflora L.	Magnoliaceae	Accepted
Maianthemum canadense Desf.	Asparagaceae	Accepted
Maihuenia poeppigii (Otto ex Pfeiff.) F.A.C.Weber.	Cactaceae	Accepted

Maireana georgei (Diels) Paul G. Wilson.	Amaranthaceae	Accepted
Malephora lutea Schwantes.	Aizoaceae	Accepted
Malephora crocea (Jacq.) Schwantes	Aizoaceae	Accepted
Malus 'Ballerina'	Rosaceae	No results
Malus domestica Borkh.	Rosaceae	Accepted
Malus pumila Mill.	Rosaceae	Accepted
Malva moschata L.	Malvaceae	Accepted
Malva neglecta Wallr.	Malvaceae	Accepted
Malva parviflora L.	Malvaceae	Accepted
Malvastrum coromandelianum (L.) Garcke	Malvaceae	Accepted
Manfreda maculosa (Hook.) Rose.	Asparagaceae	Accepted
Manfreda virginica (L.) Salisb. ex Rose.	Asparagaceae	Accepted
Marsilea drummondii A. Braun		Unresolved
Matricaria chamomilla L.	Compositae	Accepted
Matricaria perforata	Compositae	Synonym of *Tripleurospermum inodorum* (L.) Sch.Bip.
Mazus pumilus (Burm.f.) Steenis.	Phrymaceae	Accepted
Mecardonia procumbens (Mill.) Small.	Plantaginaceae	Accepted
Medicago arborea L.	Leguminosae	Accepted
Medicago lupulina L.	Leguminosae	Accepted
Medicago polymorpha L.	Leguminosae	Accepted
Medicago sativa L.	Leguminosae	Accepted
Meeboldina scariosa (R.Br.) B.G.Briggs & L.A.S.Johnson	Restionaceae	Accepted
Melampodium leucanthum Torr. & A.Gray.	Compositae	Accepted
Melica ciliata L.	Poaceae	Accepted
Melica macra Nees	Poaceae	Accepted

Melilotus albus Medik.	Leguminosae	Accepted
Melilotus officinalis (L.) Pall.	Leguminosae	Accepted
Melinis repens (Willd.) Zizka.	Poaceae	Accepted
Melissa officinalis L.	Lamiaceae	Accepted
Melissa officinalis subsp. altissima (Sm.) Arcang.	Lamiaceae	Synonym of *Melissa officinalis* L.
Melothria pendula L.	Cucurbitaceae	Accepted
Melstoma malabathricum L.	Melastomataceae	Accepted
Mentha aquatica L.	Lamiaceae	Accepted
Mentha × *piperita* L.	Lamiaceae	Accepted
Mentha pulegium L.	Lamiaceae	Accepted
Mentha spicata L.	Lamiaceae	Accepted
Mesembryanthemum barbatum	Aizoaceae	Unresolved
Microlepia hancei Prantl.	Dennstaedtiaceae	Accepted
Micromyrtus leptocalyx (F.Muell.) Benth.	Myrtaceae	Accepted
Microseris lanceolata (Walp.) Sch.Bip**.**	Compositae	Accepted
Microtis unifolia (G.Forst.) Rchb.f.	Orchidaceae	Accepted
Microlaena stipoides (Labill.) R.Br.	Poaceae	Synonym of *Ehrharta stipoides* Labill.
Mimosa pudica L.	Leguminosae	Accepted
Mimulus aurantiacus Curtis.	Phrymaceae	Accepted
Mimulus cardinalis Douglas ex Benth.	Phrymaceae	Accepted
Mimulus guttatus DC.	Phrymaceae	Accepted
Minuartia groenlandica (Retz.) Ostenf.	Caryophyllaceae	Accepted
Minuartia hybrida (Vill.) Schischk.	Caryophyllaceae	Accepted
Minuartia michauxii (Fenzl) Farw.	Caryophyllaceae	Accepted
Minuria leptophylla DC.	Compositae	Accepted
Miscanthus sinensis Andersson	Poaceae	Accepted

Miscanthus sinensis 'Silver Arrow'	Poaceae	Variety of Miscanthus sinensis Andersson.
Molinia caerulea (L.) Moench.	Poaceae	Accepted
Mollugo verticillata L.	Molluginaceae	Accepted
Momordica charantia L.	Cucurbitaceae	Accepted
Monarda didyma L.	Lamiaceae	Accepted
Monarda fistulosa L.	Lamiaceae	Accepted
Monarda punctata L.	Lamiaceae	Accepted
Monardella villosa Benth.	Lamiaceae	Accepted
Morella californica	Myricaceae	Synonym of *Myrica californica* Cham. & Schltdl.
Morella pensylvanica	Myricaceae	Unresolved
Mucuna pruriens (L.) DC.	Leguminosae	Accepted
Muhlenbergia cuspidata (Torr.) Rydb.	Poaceae	Accepted
Muhlenbergia emersleyi Vasey.	Poaceae	Accepted
Muhlenbergia mexicana (L.) Trin.	Poaceae	Accepted
Muhlenbergia montana (Nutt.) Hitchc.	Poaceae	Accepted
Muhlenbergia reverchonii Vasey & Scribn.	Poaceae	Accepted
Muhlenbergia rigens (Benth.) Hitchc.	Poaceae	Accepted
Murraya paniculata (L.) Jack.	Rutaceae	Accepted
Muscari arachnoideum	Asparagaceae	No results
Muscari armeniacum Leichtlin ex Baker.	Asparagaceae	Accepted
Muscari azureum	Asparagaceae	Synonym of *Pseudomuscari azureum* (Fenzl) Garbari & Greuter.

Muscari comosum	Asparagaceae	Synonym of *Leopoldia comosa* (L.) Parl.
Myoporum parvifolium	Scrophulariaceae	Unresolved
Myosotis arvensis (L.) Hill.	Boraginaceae	Accepted
Myosotis discolor Pers.	Boraginaceae	Accepted
Myosotis ramosíssima Rochel.	Boraginaceae	Accepted
Myosotis stricta Link ex Roem. & Schult.	Boraginaceae	Accepted
Myosotis sylvatica Hoffm.	Boraginaceae	Accepted
Nama undulatum Kunth.	Boraginaceae	Accepted
Narcissus bulbocodium var. conspicuus (Haw.) Baker	Amaryllidaceae	Synonym of *Narcissus bulbocodium subsp. quintanilhae* A.Fern.
Narcissus cyclamineus DC.	Amaryllidaceae	Accepted
Narcissus tazetta L.	Amaryllidaceae	Accepted
Nassella neesiana (Trin. & Rupr.) Barkworth	Poaceae	Accepted
Nassella pulchra (Hitchc.) Barkworth.	Poaceae	Accepted
Nassella tenuissima (Trin.) Barkworth.	Poaceae	Accepted
Nectaroscordum siculum (Ucria) Lindl.	Amaryllidaceae	Synonym of Allium siculum Ucria
Nemophila menziesii Hook. & Arn.	Boraginaceae	Accepted
Neomarica longifolia (Link & Otto) Sprague.	Iridaceae	Accepted
Neoregelia 'Fireball'	Bromeliaceae	No results
Neoregelia concentrica (Vell.) L.B.Sm.	Bromeliaceae	Accepted
Neoregelia cruenta (Graham) L.B.Sm.	Bromeliaceae	Accepted
Neottia auriculata (Wiegand) Szlach.	Orchidaceae	Accepted

Nepeta × faasenii Bergmans ex Stearn.	Lamiaceae	Accepted
Nepeta cataria L.	Lamiaceae	Accepted
Nepeta racemosa Lam.	Lamiaceae	Accepted
Nephrolepis bisserata (Sw.) Schott.	Nephrolepidaceae	Accepted
Nephrolepis exaltata (L.) Schott.	Nephrolepidaceae	Accepted
Nephrolepis multiflora (Roxb.) F.M. Jarrett ex C.V. Morton	Nephrolepidaceae	Accepted
Nerium oleander 'pink'	Apocynaceae	Variety of *Nerium oleander* L.
Neyraudia reynaudiana (Kunth) Keng ex Hitchc.	Poaceae	Accepted
Nierembergia linariifolia Graham	Solanaceae	Accepted
Nigella damascena L.	Ranunculaceae	Accepted
Nolina microcarpa S.Watson.	Asparagaceae	Accepted
Nothoscordum arenarium Herter	Amaryllidaceae	Accepted
Ocimum basilicum L.	Lamiaceae	Accepted
Ocinum x citriodolum	Lamiaceae	Synonym of *Ocimum × africanum* Lour.
Oenothera biennis L.	Onagraceae	Accepted
Oenothera caespitosa	Onagraceae	Unresolved
Oenothera elata ssp.hookeri	Onagraceae	Synonym of *Oenothera elata* Kunth.
Oenothera fruticosa ssp. glauca (Michx.) Straley.	Onagraceae	Accepted
Oenothera lamarckiana	Onagraceae	Unresolved
Oenothera macrocarpa Nutt.	Onagraceae	Accepted
Oenothera rhombipetala Nutt. ex Torr. & A. Gray	Onagraceae	Accepted
Oenothera speciosa Nutt.	Onagraceae	Accepted
Olea 'Garden Harvest' (Olive)	Oleaceae	No results

Olearia axillaris (DC.) F.Muell. ex Benth.	Compositae	Accepted
Olearia pannosa Hook.	Compositae	Accepted
Oligoneuron album	Compositae	Synonym of *Solidago ptarmicoides* (Torr. & A.Gray) B.Boivin.
Olsynium biflorum (Thunb.) Goldblatt.	Iridaceae	Accepted
Onobrychis viciifolia Scop.	Leguminosae	Accepted
Ononis striata Gouan.	Leguminosae	Accepted
Ophiopogon intermedius 'Argenteo-marginatus'	Asparagaceae	Variety of *Ophiopogon intermedius* D.Don.
Ophiopogon jaburan (Siebold) Lodd.	Asparagaceae	Accepted
Ophiopogon japonicus (Thunb.) Ker Gawl.	Asparagaceae	Accepted
Ophiopogon japonicus 'Nanus'	Asparagaceae	Variety of *Ophiopogon japonicus* (Thunb.) Ker Gawl.
Ophrys insectifera L.	Orchidaceae	Accepted
Opisthopappus taihangensis (Ling) C.Shih.	Compositae	Accepted
Opuntia aurea E.M. Baxter.	Cactaceae	Accepted
Opuntia ficus-indica (L.) Mill.	Cactaceae	Accepted
Opuntia fragilis (Nutt.) Haw.	Cactaceae	Accepted
Opuntia humifusa (Raf.) Raf.	Cactaceae	Accepted
Opuntia phaeacantha Engelm.	Cactaceae	Accepted
Opuntia polyacantha Haw.	Cactaceae	Accepted
Orchis laxiflora	Orchidaceae	Synonym of *Anacamptis laxiflora* (Lam.) R.M.Bateman, Pridgeon & M.W.Chase.
Organum x majoricum Cambess.	Lamiaceae	Accepted
Origanum dayi Post.	Lamiaceae	Accepted

Origanum dictamnus L.	Lamiaceae	Accepted
Origanum laevigatum 'Herrenhausen'	Lamiaceae	Variety of *Origanum laevigatum* Boiss.
Origanum majorana L.	Lamiaceae	Accepted
Origanum onites L.	Lamiaceae	Accepted
Origanum vulgare L.	Lamiaceae	Accepted
Origanum vulgare subsp. hirtum (Link) Ietsw.	Lamiaceae	Accepted
Ornithogalum umbellatum L.	Asparagaceae	Accepted
Ornithopus perpusillus L.	Leguminosae	Accepted
Orostachys boehmeri	Crassulaceae	Unresolved
Orthrosanthus chimboracensis (Kunth) Baker	Iridaceae	Accepted
Orthrosanthus multiflorus Sweet	Iridaceae	Accepted
Oscularia deltoides (L.) Schwantes	Aizoaceae	Synonym of *Lampranthus deltoides* (L.) Glen ex Wijnands
Osmanthus fragrans Lour.	Oleaceae	Accepted
Osmoxylon lineare (Merr.) Philipson.	Araliaceae	Accepted
Otanthus maritimus (L.) Hoffmanns. & Link.	Compositae	Accepted
Oxalis articulata Savigny	Oxalidaceae	Accepted
Oxalis conorrhiza Jacq.	Oxalidaceae	Accepted
Oxalis corniculata L.	Oxalidaceae	Accepted
Oxalis debilis subsp. corymbosa (DC.) Lourteig.	Oxalidaceae	Accepted
Oxalis pes-caprae L.	Oxalidaceae	Accepted
Oxalis stricta L.	Oxalidaceae	Accepted
Oxytropis campestris (L.) DC.	Leguminosae	Accepted
Oxytropis deflexa (Pall.) DC.	Leguminosae	Accepted
Oxytropis lambertii Pursh.	Leguminosae	Accepted
Pachyveria pachyphytoides	Crassulaceae	Unresolved

Padus avium Mill.	Rosaceae	Synonym of *Prunus padus* L.
Padus serotina (Ehrh.) Borkh.	Rosaceae	Synonym of *Prunus serotina* Ehrh.
Paliurus spina-christi Mill.	Rhamnaceae	Accepted
Pandanus amaryllifolius Roxb.	Pandanaceae	Accepted
Pandanus pygmaeus Thouars.	Pandanaceae	Accepted
Panicum bergii Arechav.	Poaceae	Accepted
Panicum dichotomiflorum Michx.	Poaceae	Accepted
Panicum hallii Vasey.	Poaceae	Accepted
Panicum lanuginosum	Poaceae	Synonym of *Panicum acuminatum* Sw.
Panicum leibergi (Vasey) Scribn.	Poaceae	Accepted
Panicum miliaceum L.	Poaceae	Accepted
Panicum philadelphicum Bernh. ex Trin.	Poaceae	Accepted
Panicum virgatum L.	Poaceae	Accepted
Papaver argemone L.	Papaveraceae	Accepted
Papaver alpinum L.	Papaveraceae	Accepted
Papaver carmeli	Papaveraceae	Unresolved
Papaver dubium L.	Papaveraceae	Accepted
Papaver rhoeas L.	Papaveraceae	Accepted
Parietaria judaica L.	Urticaceae	Accepted
Paronychia kapela ssp. serpyllifolia (Chaix) Graebn.	Caryophyllaceae	Accepted
Parthenium hysterophorus L.	Compositae	Accepted
Parthenocissus quinquefolia (L.) Planch.	Vitaceae	Accepted
Parthenium integrifoilum L.	Compositae	Accepted
Pascopyron smithii	Poaceae	Synonym of *Elymus smithii* (Rydb.) Gould.
Paspalum conjugatum P.J.Bergius.	Poaceae	Accepted
Paspalum dilatatum Poir.	Poaceae	Accepted

Paspalum notatum Flüggé.	Poaceae	Accepted
Paspalum paniculatum L.	Poaceae	Accepted
Paspalum setaceum Michx.	Poaceae	Accepted
Paspalum vaginatum Sw.	Poaceae	Accepted
Passiflora foetida L.	Passifloraceae	Accepted
Pedilanthus tithymaloides	Euphorbiaceae	Synonym of *Euphorbia tithymaloides* L.
Pedilanthus tithymaloides cv. 'Nanus'	Euphorbiaceae	Variety of *Euphorbia tithymaloides* L.
Pediocactus simpsonii (Engelm.) Britton & Rose.	Cactaceae	Accepted
Pelargonium × *hortorum* L.H. Bailey.	Geraniaceae	Accepted
Pelargonium odoratissimum (L.) L'Hér.	Geraniaceae	Accepted
Pelargonium rodneyanum	Geraniaceae	Unresolved
Pennisetum alopecuroides (L.) Spreng.	Poaceae	Accepted
Pennisetum clandestinum Hochst. ex Chiov.	Poaceae	Accepted
Pennisetum purpureum Schumach.	Poaceae	Accepted
Pennisetum setaceum (Forssk.) Chiov.	Poaceae	Accepted
Pennisetum x advena 'Rebrum'	Poaceae	Variety of *Pennisetum advena* Wipff & Veldkamp.
Penstemon angustifolius Nutt. ex Pursh.	Plantaginaceae	Accepted
Penstemon barbatus var. praecox f. nanus 'Rondo'	Plantaginaceae	Variety of *Penstemon barbatus* (Cav.) Roth.
Penstemon caespitosus Nutt. ex A. Gray.	Plantaginaceae	Accepted
Penstemon cyananthus Hook.	Plantaginaceae	Accepted

Penstemon digitalis Nutt. ex Sims.	Plantaginaceae	Accepted
Penstemon fendleri Torr. & A. Gray.	Plantaginaceae	Accepted
Penstemon grahamii D.D. Keck ex E.H. Graham.	Plantaginaceae	Accepted
Penstemon grandiflora	Plantaginaceae	Synonym of *Penstemon grandiflorus* Nutt.
Penstemon grandiflorus Nutt.	Plantaginaceae	Accepted
Penstemon heterophyllus Lindl.	Plantaginaceae	Accepted
Penstemon hirsutus (L.) Willd.	Plantaginaceae	Accepted
Penstemon linarioides ssp. coloradensis (A. Nelson) D.D. Keck.	Plantaginaceae	Accepted
Penstemon pachyphyllus A. Gray ex Rydb.	Plantaginaceae	Accepted
Penstemon pinifolius Greene.	Plantaginaceae	Accepted
Penstemon procerus Douglas ex Graham.	Plantaginaceae	Accepted
Penstemon secundiflorus Benth.	Plantaginaceae	Accepted
Penstemon strictus Benth.	Plantaginaceae	Accepted
Penstemon tenuiflorus Pennell.	Plantaginaceae	Accepted
Penstemon triflorus A. Heller.	Plantaginaceae	Accepted
Pentapogon quadrifidus (Labill.) Baill.	Poaceae	Accepted
Peperomia pellucida (L.) Kunth.	Piperaceae	Accepted
Pernettya prostrata (Cav.) DC.	Ericaceae	Synonym of *Gaultheria myrsinoides* Kunth
Perovskia atriplicifolia 'Little Spire'	Lamiaceae	Cultivar of *Perovskia atriplicifolia* Benth.
Persicaria capitata (Buch.-Ham. ex D.Don) H.Gross.	Polygonaceae	Accepted
Persicaria lapathifolia (L.) Delarbre	Polygonaceae	Accepted
Persicaria maculosa Gray	Polygonaceae	Accepted
Persoonia daphnoides A.Cunn. ex R.Br.	Proteaceae	Accepted

Persoonia oxycoccoides Sieber ex Schult.	Proteaceae	Accepted
Petrophytum caespitosum (Nutt. ex Torr. & A.Gray) Rydb.	Rosaceae	Accepted
Petrorhagia prolifera (L.) P.W.Ball & Heywood.	Caryophyllaceae	Accepted
Petrorhagia saxifraga (L.) Link.	Caryophyllaceae	Accepted
Petroselinum crispum (Mill.) Fuss	Apiaceae	Accepted
Petunia hybrid 'Headliner Red Star'	Solanaceae	Variety of *Petunia hybrida* Vilm.
Petunia hybrida Vilm.	Solanaceae	Accepted
Peucedanum japonicum Thunb.	Apiaceae	Accepted
Peucedanum oreoselinum (L.) Moench.	Apiaceae	Accepted
Phacelia californica Cham.	Boraginaceae	Accepted
Phacelia campanularia A. Gray.	Boraginaceae	Accepted
Phagnalon saxatile (L.) Cass.	Compositae	Accepted
Phalaris arundinacea L.	Poaceae	Accepted
Phaseolus vulgaris L.	Leguminosae	Accepted
Phedimus aizoon (L.) 't Hart	Crassulaceae	Synonym of *Sedum aizoon* L.
Phedimus floriferus cv. "Weihenstephaner Gold"	Crassulaceae	Synonym of *Sedum kamtschaticum* Fisch.
Phedimus kamtschaticus	Crassulaceae	Synonym of *Sedum kamtschaticum* Fisch.
Phedimus selskianus (Regel & Maack) 't Hart.	Crassulaceae	Accepted
Phedimus spurius	Crassulaceae	Synonym of *Sedum spurium* M.Bieb.
Phedimus spurius 'Leningrad White'	Crassulaceae	Variety of *Sedum spurium* M.Bieb.
Phemeranthus calycinus	Talinaceae	Synonym of *Talinum calycinum* Engelm.

Phemeranthus teretifolius (Pursh) Raf.	Montiaceae	Accepted
Phillyrea angustifolia L.	Oleaceae	Accepted
Philodendron hastatum K.Koch & Sello.	Araceae	Accepted
Philodendron warszewiczii K.Koch & C.D.Bouché.	Araceae	Accepted
Phleum boehmeri	Poaceae	Synonym of *Phleum phleoides* (L.) H.Karst.
Phleum pratense L.	Poaceae	Accepted
Phlomis fruticosa L.	Lamiaceae	Accepted
Phlomis lychnitis L.	Lamiaceae	Accepted
Phlox bifida 'Betty Blake'	Polemoniaceae	Variety of *Phlox bifida* L.C. Beck.
Phlox bífida L.C. Beck.	Polemoniaceae	Accepted
Phlox divaricata L.	Polemoniaceae	Accepted
Phlox douglasii Hook.	Polemoniaceae	Accepted
Phlox pilosa L.	Polemoniaceae	Accepted
Phlox subulata L.	Polemoniaceae	Accepted
Photinia serrulata	Rosaceae	Synonym of *Photinia bodinieri* H.L,v.
Phragmites communis	Poaceae	Synonym of *Phragmites australis* (Cav.) Trin. ex Steud.
Phragmites karka (Retz.) Trin. ex Steud.	Poaceae	Accepted
Phyla nodiflora (L.) Greene.	Verbenaceae	Accepted
Phyllanthus cochinchinensis Spreng.	Phyllanthaceae	Accepted
Phyllanthus myrtifolius (Wight) Müll.Arg.	Phyllanthaceae	Accepted
Phyllanthus urinaria L.	Phyllanthaceae	Accepted
Physaria bellii G.A. Mulligan.	Brassicaceae	Accepted
Phytolacca americana L.	Phytolaccaceae	

Picris echioides	Compositae	Synonym of *Helminthotheca echioides* (L.) Holub.
Picris hieracioides Sibth. & Sm.	Compositae	Accepted
Pilea libanensis Urb.	Urticaceae	Unresolved
Pilea microphylla (L.) Liebm.	Urticaceae	Accepted
Pilosella officinarum Vaill.	Compositae	Accepted
Pilosella rubra (Peter) Soják.	Compositae	Accepted
Pimpinella saxifraga L.	Apiaceae	Accepted
Pinus mugo Turra.	Pinaceae	Accepted
Pinus taeda L.	Pinaceae	Accepted
Piper sarmentosum Roxb.	Piperaceae	Accepted
Pisonia grandis R. Br.	Nyctaginaceae	Accepted
Pistacia lentiscus L.	Anacardiaceae	Accepted
Pisum sativum L.	Leguminosae	Accepted
Pitosporum tobira (Thunb.) W.T.Aiton.	Pittosporaceae	Accepted
Pityrogramma calomelanos (L.) Link.	Pteridaceae	Accepted
Pixzee Peach		No results
Plagiobothrys nothofulvus (A. Gray) A. Gray	Boraginaceae	Accepted
Plantago afra L.	Plantaginaceae	Accepted
Plantago coronopus L.	Plantaginaceae	Accepted
Plantago erecta E. Morris.	Plantaginaceae	Accepted
Plantago gaudichaudii Barnéoud	Plantaginaceae	Accepted
Plantago lanceolata L.	Plantaginaceae	Accepted
Plantago major L.	Plantaginaceae	Accepted
Plantago marítima L.	Plantaginaceae	Accepted
Plantago maritima subsp serpentina	Plantaginaceae	Synonym of *Plantago maritima* L.
Plantago media L.	Plantaginaceae	Accepted
Platylobium obtusangulum Hook.	Leguminosae	Accepted

Plectranthus amboinicus (Lour.) Spreng.	Lamiaceae	Accepted
Plectranthus barbatus Andrews	Lamiaceae	Accepted
Plectranthus scutellarioides (L.) R.Br.	Lamiaceae	Accepted
Plectranthus socotranus A.R.Sm.	Lamiaceae	Accepted
Pleurochaete squarrosa (Brid.) Lindb.	Pottiaceae	Accepted
Poa alpina L.	Poaceae	Accepted
Poa annua L.	Poaceae	Accepted
Poa badensis Haenke ex Willd.	Poaceae	Accepted
Poa bulbosa L.	Poaceae	Accepted
Poa compressa L.	Poaceae	Accepted
Poa fendleriana (Steud.) Vasey.	Poaceae	Accepted
Poa glauca Vahl	Poaceae	Accepted
Poa hiemata Vickery.	Poaceae	Accepted
Poa lanuginosa Poir.	Poaceae	Accepted
Poa morrisii Vickery	Poaceae	Accepted
Poa nemoralis L.	Poaceae	Accepted
Poa pratensis L.	Poaceae	Accepted
Poa pratensis sp. angustifolia	Poaceae	Synonym of *Poa angustifolia* L.
Poa trivialis L.	Poaceae	Accepted
Podolepis jaceoides (Sims) Voss	Compositae	Accepted
Pogonatherum crinitum (Thunb.) Kunth.	Poaceae	Accepted
Pogostemon cablin (Blanco) Benth.	Lamiaceae	Accepted
Pohlia nutans (Hedw.) Lindb.	Bryaceae	Accepted
Polemonium reptans L.	Polemoniaceae	Accepted
Polycarpon tetraphyllum (L.) L.	Caryophyllaceae	Accepted
Polygala myrtifolia L.	Polygalaceae	Accepted
Polygala verticillata L.	Polygalaceae	Accepted
Polygala vulgaris L.	Polygalaceae	Accepted

Polygonum aviculare L.	Polygonaceae	Accepted
Polygonum persicaria L.	Polygonaceae	Synonym of Persicaria maculosa Gray
Polygonum tenue Michx.	Polygonaceae	Accepted
Polypodium vulgare L.	Polypodiaceae	Accepted
Polystichum munitum (Kaulf.) C. Presl.	Dryopteridaceae	Accepted
Polytrichum commune Hedw.	Polytrichaceae	Accepted
Polytrichum juniperinum Hedw.	Polytrichaceae	Accepted
Polytrichum piliferum Hedw.	Polytrichaceae	Accepted
Populus alba L.	Salicaceae	Accepted
Populus deltoides Marshall	Salicaceae	Accepted
Populus tremula L.	Salicaceae	Accepted
Portulaca 'Hana Misteria'	Portulacaceae	No results
Portulaca gilliesii Hook.	Portulacaceae	Accepted
Portulaca grandiflora Hook.	Portulacaceae	Accepted
Portulaca maculate	Portulacaceae	No results
Portulaca oleracea L.	Portulacaceae	Accepted
Portulaca pilosa L.	Portulacaceae	Accepted
Portulacaria afra f. variegata	Didiereaceae	Synonym of *Portulacaria afra* Jacq.
Portulacaria afra Jacq.	Didiereaceae	Accepted
Potentilla anserina L.	Rosaceae	Accepted
Potentilla argentea L.	Rosaceae	Accepted
Potentilla arguta Pursh.	Rosaceae	Accepted
Potentilla canadensis L.	Rosaceae	Accepted
Potentilla crantzii (Crantz) Beck ex Fritsch.	Rosaceae	Accepted
Potentilla erecta (L.) Raeusch.	Rosaceae	Accepted
Potentilla hyparctica Malte.	Rosaceae	Accepted
Potentilla nepalensis Hook.	Rosaceae	Accepted
Potentilla neumanniana Rchb.	Rosaceae	Accepted
Potentilla norvegica L.	Rosaceae	Accepted

Potentilla pusilla Host.	Rosaceae	Accepted
Potentilla reptans L.	Rosaceae	Accepted
Potentilla tabernaemontani Asch.	Rosaceae	Accepted
Potentilla verna	Rosaceae	Synonym of *Potentilla grandiflora* L.
Pouzolzia zeylanica (L.) Benn.	Urticaceae	Accepted
Prasium majus L.	Lamiaceae	Accepted
Prenanthes trifoliolata	Compositae	Synonym of *Nabalus trifoliolatus* Cass.
Primula acaulis (L.) Hill	Primulaceae	Synonym of *Primula vulgaris* Huds.
Primula veris L.	Primulaceae	Accepted
Primula vulgaris Huds.	Primulaceae	Accepted
Prostanthera aspalathoides A.Cunn. ex Benth.	Lamiaceae	Accepted
Prostanthera incisa R.Br.	Lamiaceae	Accepted
Prostanthera nivea A.Cunn. ex Benth.	Lamiaceae	Accepted
Prunella grandiflora (L.) Scholler.	Lamiaceae	Accepted
Prunella vulgaris L.	Lamiaceae	Accepted
Prunella vulgaris subsp. lanceolata (W.P.C.Barton) Piper & Beattie.	Lamiaceae	Accepted
Prunella vulgaris subsp. vulgaris	Lamiaceae	Synonym of *Prunella vulgaris* L.
Prunus mahaleb L.	Rosaceae	Accepted
Prunus salicina Lindl.	Rosaceae	Accepted
Prunus spinosa L.	Rosaceae	Accepted
Pseudolysimachion spicatum	Plantaginaceae	Synonym of *Veronica spicata* L.
Pteridium aquilinum var. pubescens	Dennstaedtiaceae	No results
Pteris vittata L.	Pteridaceae	Accepted
Pterocypsela indica	Compositae	Synonym of *Lactuca indica* L.

Ptilotus exaltatus	Amaranthaceae	Unresolved
Ptilotus macrocephalus (R. Br.) Poir.	Amaranthaceae	Accepted
Ptilotus spathulatus (R. Br.) F. Muell.	Amaranthaceae	Accepted
Ptychostomum capillare (Hedw.) D. T. Holyoak & N. Pedersen	Bryaceae	Accepted
Pulsatilla patens	Ranunculaceae	Synonym of *Anemone patens* L.
Pulsatilla vulgaris	Ranunculaceae	Synonym of *Anemone pulsatilla* L.
Punica granatum L.	Lythraceae	Accepted
Puschkinia libanotica	Asparagaceae	Synonym of *Puschkinia scilloides* Adams.
Putoria calabrica	Rubiaceae	Synonym of *Plocama calabrica* (L.f.) M.Backlund & Thulin.
Pycnanthemum tenuifolium Schrad.	Lamiaceae	Accepted
Pycnanthemum virginianum (L.) T.Durand & B.D.Jacks. ex B.L.Rob. & Fernald.	Lamiaceae	Accepted
Pycnosorus globosus F.L.Bauer ex Benth.	Compositae	Accepted
Pycreus flavidus (Retz.) T.Koyama.	Cyperaceae	Accepted
Pycreus polystachyos (Rottb.) P.Beauv.	Cyperaceae	Accepted
Pycreus pumilus (L.) Nees.	Cyperaceae	Accepted
Pyrus communis L.	Rosaceae	Accepted
Pyrus pyraster (L.) Burgsd.	Rosaceae	Accepted
Quercus petraea (Matt.) Liebl.	Fagaceae	Accepted
Quercus robur L.	Fagaceae	Accepted
Racomitrium canescens	Grimmiaceae	Synonym of *Niphotrichum canescens*

		(Hedw.) Bednarek-Ochyra & Ochyra.
Ranunculus acris L.	Ranunculaceae	Accepted
Ranunculus bulbosus L.	Ranunculaceae	Accepted
Ranunculus californicus Benth.	Ranunculaceae	Accepted
Ranunculus polyanthemus L.	Ranunculaceae	Accepted
Ranunculus repens L.	Ranunculaceae	Accepted
Ranunculus scandicinus	Ranunculaceae	Unresolved
Raphanus raphanistrum subsp. sativus (L.) Domin	Brassicaceae	Accepted
Raphanus sativus	Brassicaceae	Synonym of *Raphanus raphanistrum subsp. sativus* (L.) Domin.
Ratibida columnifera (Nutt.) Wooton & Standl.	Compositae	Accepted
Ratibida pinnata (Vent.) Barnhart.	Compositae	Accepted
Ravenea rivularis Jum. & H.Perrier	Arecaceae	Accepted
Reichardia picroides (L.) Roth	Compositae	Accepted
Reseda alba L.	Resedaceae	Accepted
Reseda lutea L.	Resedaceae	Accepted
Reseda odorata L.	Resedaceae	Accepted
Rhinanthus minor L.	Orobanchaceae	Accepted
Rhodanthe anthemoides (Sieber ex Spreng.) Paul G.Wilson.	Compositae	Accepted
Rhodiola pachyclados (Aitch. & Hemsl.) H. Ohba.	Crassulaceae	Accepted
Rhodiola rosea	Crassulaceae	Synonym of *Sedum roseum* (L.) Scop.
Rhodiola trollii	Crassulaceae	No results
Rhododendron simsii Planch.	Ericaceae	Accepted
Rhodomyrtus tomentosa (Aiton) Hassk.	Myrtaceae	Accepted

Rhoeo spathaceo	Commelinaceae	Synonym of *Tradescantia spathacea* Sw.
Rhoeo spathaceo cv. compacta	Commelinaceae	Synonym of *Tradescantia spathacea* Sw.
Rhynchospora nervosa (Vahl) Boeckeler	Cyperaceae	Accepted
Ribes speciosum Pursh.	Grossulariaceae	Accepted
Ricotia lunaria (L.) DC.	Brassicaceae	Accepted
Robinia pseudoacacia L.	Leguminosae	Accepted
Rorippa indica (L.) Hiern	Brassicaceae	Accepted
Rosa californica Cham. & Schltdl.	Rosaceae	Accepted
Rosa canina L.	Rosaceae	Accepted
Rosa carolina L.	Rosaceae	Accepted
Rosa luciae Franch. & Rochebr.	Rosaceae	Accepted
Rosmarinus officinalis L.	Lamiaceae	Accepted
Rubia peregrina L.	Rubiaceae	Accepted
Rubus canescens DC.	Rosaceae	Accepted
Rubus flagellaris Willd.	Rosaceae	Accepted
Rubus parvifolius L	Rosaceae	Accepted
Rubus plicatus Weihe & Nees	Rosaceae	Accepted
Rubus ursinus Cham. & Schltdl.	Rosaceae	Accepted
Rudbeckia fulgida Aiton.	Compositae	Accepted
Rudbeckia fulgida var. deamii (S.F.Blake) Perdue.	Compositae	Accepted
Rudbeckia hirta L.	Compositae	Accepted
Rudbeckia laciniata L.	Compositae	Accepted
Rudbeckia speciosa Schrad.	Compositae	Accepted
Rudbeckia subtomentosa Pursh.	Compositae	Accepted
Rudbeckia sullivantii 'Goldsturm'	Compositae	Synonym of *Rudbeckia fulgida var. sullivantii* (C.L.Boynton & Beadle) Cronquist.
Rudbeckia triloba L.	Compositae	Accepted

Ruellia brittoniana	Acanthaceae	Synonym of *Ruellia simplex* C.Wright.
Ruellia humilis Nutt.	Acanthaceae	Accepted
Rumex acetosella L.	Polygonaceae	Accepted
Rumex crispus L.	Polygonaceae	Accepted
Rumex dumosus A.Cunn. ex Meisn.	Polygonaceae	Accepted
Rumex obtusifolius L.	Polygonaceae	Accepted
Rumex scutatus L.	Polygonaceae	Accepted
Ruschia pulvinaris L. Bolus	Aizoaceae	Accepted
Rutidosis leptorhynchoides F.Muell.	Compositae	Accepted
Rytidosperma caespitosum (Gaudich.) Connor & Edgar	Poaceae	Accepted
Rytidosperma fulvum (Vickery) A.M.Humphreys & H.P.Linder	Poaceae	Unresolved
Rytidosperma racemosum (R.Br.) Connor & Edgar	Poaceae	Accepted
Rytidosperma setaceum (R.Br.) Connor & Edgar	Poaceae	Accepted
Sabatia angularis (L.) Pursh.	Gentianaceae	Accepted
Sacciolepis indica (L.) Chase	Poaceae	Accepted
Sagina procumbens L.	Caryophyllaceae	Accepted
Sagina subulata (Sw.) C.Presl	Caryophyllaceae	Accepted
Salsola inermis Forssk.	Amaranthaceae	Accepted
Salvia × sylvestris 'Blauhügel'	Lamiaceae	Variety of *Salvia × sylvestris* L.
Salvia azurea Michx. ex Vahl.	Lamiaceae	Accepted
Salvia cryptantha	Lamiaceae	Synonym of *Salvia absconditiflora* Greuter & Burdet.
Salvia elegans Vahl	Lamiaceae	Accepted
Salvia 'Eskihar'	Lamiaceae	No results
Salvia fruticosa Mill.	Lamiaceae	Accepted

Salvia guaranitica A.St.-Hil. ex Benth.	Lamiaceae	Synonym of *Salvia coerulea* Benth.
Salvia greggii A.Gray.	Lamiaceae	Accepted
Salvia mellifera Greene.	Lamiaceae	Accepted
Salvia nemorosa L.	Lamiaceae	Accepted
Salvia dorrii (Kellogg) Abrams	Lamiaceae	Accepted
Salvia officinalis 'Berggarten'	Lamiaceae	Variety of *Salvia officinalis* L.
Salvia officinalis L.	Lamiaceae	Accepted
Salvia pachyphylla Epling ex Munz.	Lamiaceae	Accepted
Salvia plebeia R.Br.	Lamiaceae	Accepted
Salvia pratensis L.	Lamiaceae	Accepted
Salvia rosmarinus Schleid.	Lamiaceae	Synonym of *Rosmarinus officinalis* L.
Salvia scutellarioides Kunth	Lamiaceae	Accepted
Salvia sonomensis Greene.	Lamiaceae	Accepted
Salvia spathacea Greene.	Lamiaceae	Accepted
Salvia verticillata L.	Lamiaceae	Accepted
Salvia xeropapillosa Fern.Alonso	Lamiaceae	Accepted
Sambucus nigra L.	Adoxaceae	Accepted
Sanchezia oblonga Ruiz & Pav.	Acanthaceae	Accepted
Sanguisorba minor Scop.	Rosaceae	Accepted
Sanguisorba minor ssp. minor	Rosaceae	Synonym of *Sanguisorba minor* Scop.
Sanguisorba tenuifolia 'Purpurea'	Rosaceae	Unresolved
Sansevieria 'Alva'	Asparagaceae	No results
Sansevieria cylindrica Bojer ex Hook.	Asparagaceae	Accepted
Sansevieria ehrenbergii Schweinf. ex Baker.	Asparagaceae	Accepted
Sansevieria hahnii hort.	Asparagaceae	Unresolved
Sansevieria trifasciata 'Laurentii'	Asparagaceae	Synonym of *Sansevieria trifasciata* Prain.

Sansevieria trifasciata cv. hahnii	Asparagaceae	Variety of *Sansevieria trifasciata* Prain.
Sansevieria trifasciata Prain.	Asparagaceae	Accepted
Santolina chamaecyparissus L.	Compositae	Accepted
Santolina rosmarinifolia L.	Compositae	Accepted
Saponaria ocymoides L.	Caryophyllaceae	Accepted
Sarcococca hookeriana var. humilis Rehder & E.H. Wilson	Buxaceae	Synonym of *Sarcococca hookeriana var. digyna* Franch.
Satureja montana L.	Lamiaceae	Accepted
Satureja repandens	Lamiaceae	No results
Satureja subspicata Bartl. ex Vis.	Lamiaceae	Accepted
Satureja thymbra L.	Lamiaceae	Accepted
Satureja vulgaris	Lamiaceae	Synonym of *Clinopodium vulgare* L.
Saxifraga arendsii	Saxifragaceae	No results
Saxifraga cespitosa L.	Saxifragaceae	Accepted
Saxifraga cotyledon L.	Saxifragaceae	Accepted
Saxifraga granulata L.	Saxifragaceae	Accepted
Saxifraga paniculata Mill.	Saxifragaceae	Accepted
Saxifraga tridactylites L.	Saxifragaceae	Accepted
Scabiosa columbaria L.	Caprifoliaceae	Accepted
Scabiosa prolifera	Caprifoliaceae	Unresolved
Scaevola aemula R.Br.	Goodeniaceae	Unresolved
Scaevola albida (Sm.) Druce	Goodeniaceae	Unresolved
Scaevola taccada (Gaertn.) Roxb.	Goodeniaceae	Accepted
Schefflera arboricola (Hayata) Merr.	Araliaceae	Accepted
Schefflera heptaphylla (L.) Frodin.	Araliaceae	Accepted
Schizachyrium condensatum (Kunth) Nees.	Poaceae	Accepted
Schizachyrium scoparium (Michx.) Nash.	Poaceae	Accepted
Schizocentron elegans	Melastomataceae	Unresolved

Schkuhria pinnata (Lam.) Kuntze ex Thell.	Compositae	Accepted
Scilla autumnalis	Asparagaceae	Synonym of *Prospero autumnale* (L.) Speta.
Scilla siberica Haw.	Asparagaceae	Accepted
Scirpus cespitosus	Cyperaceae	Synonym of *Trichophorum cespitosum* (L.) Hartm.
Scirpus cyperinus (L.) Kunth	Cyperaceae	Accepted
Scirpus grossus	Cyperaceae	Synonym of *Actinoscirpus grossus* (L.f.) Goetgh. & D.A.Simpson.
Scirpus juncoides	Cyperaceae	Synonym of *Schoenoplectiella juncoides* (Roxb.) Lye.
Scleranthus annuus subsp. polycarpos (L.) Bonnier & Layens.	Caryophyllaceae	Accepted
Scleranthus biflorus (J.R. Forst. & G. Forst.) Hook. f.	Caryophyllaceae	Synonym of *Mniarum biflorum* J.R. Forst. & G. Forst.
Scleria triglomerata Michx.	Cyperaceae	Accepted
Scoparia dulcis L.	Plantaginaceae	Accepted
Scoparia montevidensis (Spreng.) R.E. Fr.	Plantaginaceae	Accepted
Scrophularia californica Cham. & Schltdl.	Scrophulariaceae	Accepted
Scrophularia canina L.	Scrophulariaceae	Accepted
Scrophularia peregrina L.	Scrophulariaceae	Accepted
Scutellaria prostrata Jacquem. ex Benth.	Lamiaceae	Accepted
Scutellaria resinosa Torr.	Lamiaceae	Accepted

Scutellaria scordifolia Fisch. ex Schrank.	Lamiaceae	Accepted
Scutellaria serrata Andrews.	Lamiaceae	Accepted
Sedaveria 'Pats Pink'	Crassulaceae	Accepted
Sedum ‘Herbstfreude	Crassulaceae	No results
Sedum ‘Rockery Challenger’	Crassulaceae	No results
Sedum ‘Spiral Staircase’	Crassulaceae	No results
Sedum acre ‘Aureum’	Crassulaceae	Variety of *Sedum acre* L.
Sedum acre ‘Golden Carpet’	Crassulaceae	Variety of *Sedum acre* L.
Sedum acre ‘Golden Queen	Crassulaceae	Variety of *Sedum acre* L.
Sedum acre ‘Minor’	Crassulaceae	Variety of *Sedum acre* L.
Sedum acre ‘Oktoberfest’	Crassulaceae	Variety of *Sedum acre* L.
Sedum acre L.	Crassulaceae	Accepted
Sedum adolphii Raym.-Hamet.	Crassulaceae	Accepted
Sedum aizoon L.	Crassulaceae	Accepted
Sedum album ‘Bella d’Inverno’	Crassulaceae	Variety of *Sedum album* L.
Sedum album ‘Coral Carpet’	Crassulaceae	Variety of *Sedum album* L.
Sedum album ‘France’ white stonecrop	Crassulaceae	Variety of *Sedum album* L.
Sedum album ‘Red Ice’	Crassulaceae	Variety of *Sedum album* L.
Sedum album L.	Crassulaceae	Accepted
Sedum album subsp. album	Crassulaceae	Synonym of *Sedum album* L.
Sedum amplexicaule subsp. tenuifolium	Crassulaceae	Synonym of *Sedum amplexicaule* DC.
Sedum anglicum Huds.	Crassulaceae	Accepted
Sedum annuum L.	Crassulaceae	Accepted
Sedum blue	Crassulaceae	No results
Sedum boehmeri	Crassulaceae	Unresolved
Sedum caeruleum L.	Crassulaceae	Accepted

Sedum cauticola	Crassulaceae	Unresolved
Sedum cauticola 'Lidakense'	Crassulaceae	Unresolved
Sedum chatre	Crassulaceae	No results
Sedum clavatum R.T. Clausen.	Crassulaceae	Accepted
Sedum confusum Hemsl.	Crassulaceae	Accepted
Sedum cyaneum J. Rudolph.	Crassulaceae	Accepted
Sedum dasyphyllum 'Burnati'	Crassulaceae	Variety of *Sedum dasyphyllum* L.
Sedum dasyphyllum 'Lilac Mound'	Crassulaceae	Variety of *Sedum dasyphyllum* L.
Sedum dasyphyllum L.	Crassulaceae	Accepted
Sedum dasyphyllum var. dasyphyllum	Crassulaceae	No results
Sedum dasyphyllum var. glanduliferum	Crassulaceae	No results
Sedum diffusum S. Watson.	Crassulaceae	Accepted
Sedum divergens S. Watson.	Crassulaceae	Accepted
Sedum ellacombianum	Crassulaceae	Synonym of *Sedum kamtschaticum* Fisch.
Sedum ewersii Ledeb.	Crassulaceae	Accepted
Sedum fino	Crassulaceae	No results
Sedum floriferum	Crassulaceae	Synonym of *Sedum kamtschaticum* Fisch.
Sedum forsterianum Sm.	Crassulaceae	Accepted
Sedum forsterianum ssp. elegans 'Silver Stone'	Crassulaceae	Synonym of *Sedum forsterianum* Sm.
Sedum glaucophyllum R.T. Clausen.	Crassulaceae	Accepted
Sedum gypsicola Boiss. & Reut.	Crassulaceae	Accepted
Sedum hispanicum 'Silver Carpet'	Crassulaceae	Variety of *Sedum hispanicum* L.
Sedum hispanicum L.	Crassulaceae	Accepted
Sedum hispanicum var. hispanicum	Crassulaceae	No results

Sedum hybridum 'Czar's Gold'	Crassulaceae	Variety of *Sedum hybridum* L.
Sedum hybridum L.	Crassulaceae	Accepted
Sedum japonicum Siebold ex Miq.	Crassulaceae	Accepted
Sedum kamtschaticum 'ellacombianum'	Crassulaceae	Synonym of *Sedum kamtschaticum* Fisch.
Sedum kamtschaticum Fisch.	Crassulaceae	Accepted
Sedum kamtschaticum var. floriferum	Crassulaceae	No results
Sedum lanceolatum Torr.	Crassulaceae	Accepted
Sedum lineare 'Variegatum'	Crassulaceae	Variety of *Sedum lineare* Thunb.
Sedum lineare cv. 'robustum'	Crassulaceae	Variety of *Sedum lineare* Thunb.
Sedum lineare Thunb.	Crassulaceae	Accepted
Sedum litoreum Guss.	Crassulaceae	Accepted
Sedum lydium Boiss.	Crassulaceae	Accepted
Sedum makinoi Maxim.	Crassulaceae	Accepted
Sedum mexicanum Britton.	Crassulaceae	Accepted
Sedum middendorffianum	Crassulaceae	Synonym of *Phedimus middendorffianus* (Maxim.) 't Hart.
Sedum middendorffianum var. diffusum	Crassulaceae	Synonym of *Phedimus middendorffianus* (Maxim.) 't Hart.
Sedum montanum Songeon & E.P.Perrier.	Crassulaceae	Accepted
Sedum montanum ssp. orientale 't Hart.	Crassulaceae	Accepted
Sedum moranense Kunth.	Crassulaceae	Accepted
Sedum nevii 'glaucophyllum'	Crassulaceae	No results
Sedum nussbaumerianum Bitter.	Crassulaceae	Accepted
Sedum obtusifolium var. listoniae	Crassulaceae	No results

Sedum ochroleucum Chaix.	Crassulaceae	Accepted
Sedum oreganum Nutt.	Crassulaceae	Accepted
Sedum oregonense (S. Watson) M. Peck.	Crassulaceae	Accepted
Sedum oryzifolium	Crassulaceae	Unresolved
Sedum pachyclados	Crassulaceae	Synonym of *Rhodiola pachyclados* (Aitch. & Hemsl.) H. Ohba
Sedum pachyphyllum Rose.	Crassulaceae	Accepted
Sedum pallidum M.Bieb	Crassulaceae	Accepted
Sedum palmieri S. Watson.	Crassulaceae	Accepted
Sedum populifolium Pall.	Crassulaceae	Accepted
Sedum pulchellum Michx.	Crassulaceae	Accepted
Sedum reflexum	Crassulaceae	Synonym of *Sedum rupestre* L.
Sedum reflexum 'Blue Spruce'	Crassulaceae	Variety of *Sedum rupestre* L.
Sedum requieni	Crassulaceae	No results
Sedum roseum (L.) Scop.	Crassulaceae	Accepted
Sedum rubens L.	Crassulaceae	Accepted
Sedum rubens var. rubens	Crassulaceae	No results
Sedum rubrotinctum R.T. Clausen.	Crassulaceae	Accepted
Sedum rupestre 'Angelina'	Crassulaceae	Variety of *Sedum rupestre* L.
Sedum rupestre L.	Crassulaceae	Accepted
Sedum sarmentosum Bunge.	Crassulaceae	Accepted
Sedum sediforme (Jacq.) Pau.	Crassulaceae	Accepted
Sedum selskianum	Crassulaceae	Synonym of *Phedimus selskianus* (Regel & Maack) 't Hart.
Sedum sexangulare L.	Crassulaceae	Accepted
Sedum sieboldii Regel.	Crassulaceae	Accepted

Sedum spathulifolium ‘ var. purpureum	Crassulaceae	Variety of *Sedum spathulifolium* Hook.
Sedum spathulifolium Hook.	Crassulaceae	Accepted
Sedum spectabile Boreau.	Crassulaceae	Accepted
Sedum spurium (Dragon’s Blood)	Crassulaceae	Variety of *Sedum spurium* M.Bieb.
Sedum spurium ‘Coccineum’	Crassulaceae	Variety of *Sedum spurium* M.Bieb.
Sedum spurium ‘Fuldaglut’	Crassulaceae	Variety of *Sedum spurium* M.Bieb.
Sedum spurium ‘Green Mantle	Crassulaceae	Variety of *Sedum spurium* M.Bieb.
Sedum spurium ‘John Creech’	Crassulaceae	Variety of *Sedum spurium* M.Bieb.
Sedum spurium ‘Roseum’	Crassulaceae	Variety of *Sedum spurium* M.Bieb.
Sedum spurium ‘Schorbuser Blut’	Crassulaceae	Variety of *Sedum spurium* M.Bieb.
Sedum spurium ‘Splendens’	Crassulaceae	Variety of *Sedum spurium* M.Bieb.
Sedum spurium ‘Summer glory’	Crassulaceae	Variety of *Sedum spurium* M.Bieb.
Sedum spurium ‘Tricolor’	Crassulaceae	Variety of *Sedum spurium* M.Bieb.
Sedum spurium ‘Voodoo’	Crassulaceae	Variety of *Sedum spurium* M.Bieb.
Sedum spurium ‘White Form’	Crassulaceae	Variety of *Sedum spurium* M.Bieb.
Sedum spurium atropurpureum	Crassulaceae	Variety of *Sedum spurium* M.Bieb.
Sedum spurium M.Bieb.	Crassulaceae	Accepted
Sedum spurium purpureum	Crassulaceae	Variety of *Sedum spurium* M.Bieb.

Sedum stahlii Solms	Crassulaceae	Accepted
Sedum stefco Stef.	Crassulaceae	Accepted
Sedum stellatum L.	Crassulaceae	Accepted
Sedum stenopetalum Pursh.	Crassulaceae	Accepted
Sedum stoloniferum S.G.Gmel.	Crassulaceae	Accepted
Sedum surculosum var. luteum	Crassulaceae	Synonym of *Monanthes atlantica* J. Ball.
Sedum takesimense "Gold Carpet'	Crassulaceae	Unresolved
Sedum telephium 'Matrona'	Crassulaceae	Variety of *Sedum telephium* L.
Sedum telephium L.	Crassulaceae	Accepted
Sedum telephium subsp. fabaria (W.D.J.Koch) Kirschl.	Crassulaceae	Synonym of *Sedum telephium* L.
Sedum telephium var. maximum	Crassulaceae	Synonym of *Sedum maximum* (L.) Suter.
Sedum ternatum Michx.	Crassulaceae	Accepted
Sedum tetractinum 'Vera Jameson'	Crassulaceae	Variety of *Sedum tetractinum* Fröd.
Sedum tetractinum Fröd.	Crassulaceae	Accepted
Sedum tomentosum	Crassulaceae	No results
Sedum urvillei DC.	Crassulaceae	Accepted
Sedum variegatum	Crassulaceae	Synonym of *Dudleya variegata* (S.Watson) Moran.
Sedum x luteoviride	Crassulaceae	Unresolved
Sedum x rubrotinctum	Crassulaceae	No results
Selliera radicans Cav.	Goodeniaceae	Accepted
Sempervivum 'Reinhard'	Crassulaceae	No results
Sempervivum 'Royal Ruby	Crassulaceae	No results
Sempervivum arachnoideum L.	Crassulaceae	Accepted
Sempervivum calcareum Jord.	Crassulaceae	Accepted
Sempervivum globiferum L.	Crassulaceae	Accepted
Sempervivum montanum L.	Crassulaceae	Accepted

Sempervivum tectorum L.	Crassulaceae	Accepted
Senecio brasiliensis (Spreng.) Less.	Compositae	Accepted
Senecio ceratophylloides Griseb.	Compositae	Accepted
Senecio cineraria DC.	Compositae	Synonym of *Jacobaea maritima* (L.) Pelser & Meijden
Senecio crassissimus	Compositae	Unresolved
Senecio inaequidens DC.	Compositae	Accepted
Senecio integrifolius Hook.	Compositae	Accepted
Senecio jacobaea	Compositae	Synonym of *Jacobaea vulgaris* Gaertn.
Senecio mandraliscae (Tineo) H.Jacobsen	Compositae	Accepted
Senecio pauperculus	Compositae	Synonym of *Packera paupercula* (Michx.) Á.Löve & D.Löve.
Senecio viscosus L.	Compositae	Accepted
Senecio vulgaris L.	Compositae	Accepted
Senna artemisiodes ssp. X coriacea	Leguminosae	No results
Serissa foetida	Rubiaceae	Synonym of *Serissa japonica* (Thunb.) Thunb.
Serissa japonica (Thunb.) Thunb.	Rubiaceae	Accepted
Sesleria caerulea (L.) Ard.	Poaceae	Accepted
Sesuvium portulacastrum (L.) L.	Aizoaceae	Accepted
Sesuvium verrucosum Raf.	Aizoaceae	Accepted
Setaria faberi R.A.W.Herrm.	Poaceae	Accepted
Setaria glauca	Poaceae	Synonym of *Pennisetum glaucum* (L.) R.Br.
Setaria parviflora (Poir.) M.Kerguelen.	Poaceae	Accepted
*Setaria verticillata (*L.) P.Beauv.	Poaceae	Accepted
Setaria viridis (L.) P.Beauv.	Poaceae	Accepted

Setcreasea pallida	Commelinaceae	Synonym of *Tradescantia pallida* (Rose) D.R.Hunt.
Setcreasea purpurea	Commelinaceae	Synonym of *Tradescantia pallida* (Rose) D.R.Hunt.
Shepherdia canadensis (L.) Nutt.	Elaeagnaceae	Accepted
Sibbaldia tridentata (Sol.) Paule & Soj k	Rosaceae	Unresolved
Sibbaldiopsis tridentata (Sol.) Rydb.	Rosaceae	Accepted
Sida acuta Burm.f.	Malvaceae	Accepted
Sida dictyocarpa Griseb. ex K.Schum.	Malvaceae	Synonym of *Sida glabra* Mill.
Sida rhombifolia L.	Malvaceae	Accepted
Sida spinosa L.	Malvaceae	Accepted
Sidalcea malviflora (DC.) A. Gray.	Malvaceae	Accepted
Sideritis athoa	Lamiaceae	Synonym of *Sideritis perfoliata subsp. athoa* (Papan. & Kokkini) Baden.
Sideritis hyssopifolia L.	Lamiaceae	Accepted
Sideritis syriaca L.	Lamiaceae	Accepted
Silene aegyptiaca (L.) L.f.	Caryophyllaceae	Accepted
Silene alba	Caryophyllaceae	Synonym of *Silene latifolia subsp. alba* (Mill.) Greuter & Burdet.
Silene conica L.	Caryophyllaceae	Accepted
Silene dioica (L.) Clairv.	Caryophyllaceae	Accepted
Silene gallica L.	Caryophyllaceae	Accepted
Silene latifólia Poir.	Caryophyllaceae	Accepted
Silene otites (L.) Wibel.	Caryophyllaceae	Accepted
Silene schafta	Caryophyllaceae	Unresolved
Silene secundiflora Otth.	Caryophyllaceae	Accepted
Silene uniflora Roth.	Caryophyllaceae	Accepted
Silene viscaria (L.) Jess.	Caryophyllaceae	Accepted

Silene vulgaris (Moench) Garcke.	Caryophyllaceae	Accepted
Silphium integrifolium Michx.	Compositae	Accepted
Silphium laciniatum L.	Compositae	Accepted
Sinapis alba L.	Brassicaceae	Accepted
Sisyrinchium albidum Raf.	Iridaceae	Accepted
Sisyrinchium angustifolium Mill.	Iridaceae	Accepted
Sisyrinchium bellum S.Watson.	Iridaceae	Accepted
Sisyrinchium bogotense Kunth	Iridaceae	Synonym of *Sisyrinchium tinctorium* Kunth
Sisyrinchium californicum (Ker Gawl.) Dryand.	Iridaceae	Accepted
Sisyrinchium campestre E.P.Bicknell.	Iridaceae	Accepted
Sisyrinchium idahoense E.P.Bicknell.	Iridaceae	Accepted
Sisyrinchium mucronatum Michx.	Iridaceae	Accepted
Sisyrinchium striatum Sm.	Iridaceae	Accepted
Smallanthus uvedalia (L.) Mack. ex Mack.	Compositae	Accepted
Solanum americanum Mill.	Solanaceae	Accepted
Solanum lycopersicum L.	Solanaceae	Accepted
Solanum melongena L.	Solanaceae	Accepted
Solanum nigrum	Solanaceae	Synonym of *Solanum americanum* Mill.
Solanum ptychanthum Dunal	Solanaceae	Accepted
Solanum tuberosum L.	Solanaceae	Accepted
Solidago bicolor L.	Compositae	Accepted
Solidago canadensis L.	Compositae	Accepted
Solidago flexicaulis L.	Compositae	Accepted
Solidago gigantea Aiton	Compositae	Accepted
Solidago juncea Aiton	Compositae	Accepted
Solidago memoralis	Compositae	Unresolved
Solidago nemoralis Aiton.	Compositae	Accepted

Solidago odora Aiton.	Compositae	Accepted
Solidago praecox	Compositae	Unresolved
Solidago ptarmicoides (Torr. & A.Gray) B.Boivin.	Compositae	Accepted
Solidago puberula Nutt.	Compositae	Accepted
Solidago rigida L.	Compositae	Accepted
Solidago sempervirens Michx.	Compositae	Accepted
Solidago speciosa A.Gray.	Compositae	Accepted
Solidago velutina ssp. californica (Nutt.) Semple.	Compositae	Accepted
Soliva anthemifolia (Juss.) Sweet.	Compositae	Accepted
Sonchus arvensis L.	Compositae	Accepted
Sonchus asper (L.) Hill	Compositae	Accepted
Sonchus oleraceus (L.) L.	Compositae	Accepted
Sonchus tenerrimus L.	Compositae	Accepted
Sorghastrum nutans (L.) Nash.	Poaceae	Accepted
Sorghum halepense (L.) Pers.	Poaceae	Accepted
Sparaxis tricolor (Schneev.) Ker Gawl.	Iridaceae	Accepted
Spartium junceum L.	Leguminosae	Accepted
Spergularia diandra (Guss.) Heldr.	Caryophyllaceae	Accepted
Spergularia rubra (L.) J.Presl & C.Presl.	Caryophyllaceae	Accepted
Spermacoce stricta	Rubiaceae	Synonym of *Oldenlandia tenelliflora* (Blume) Kuntze.
Spermacoce verticillata L.	Rubiaceae	Accepted
Sphaeralcea australis Speg.	Malvaceae	Accepted
Sphagneticola trilobata (L.) Pruski.	Compositae	Accepted
Spigelia anthelmia L.	Loganiaceae	Accepted
Spinacia oleracea L.	Amaranthaceae	Accepted
Spiranthes hongkongensis S.Y.Hu & Barretto.	Orchidaceae	Accepted

Spiranthes vernalis Engelm. & A.Gray.	Orchidaceae	Accepted
Sporobolus airoides (Torr.) Torr.	Poaceae	Accepted
Sporobolus cryptandrus (Torr.) A.Gray.	Poaceae	Accepted
Sporobolus fertilis (Steud.) Clayton.	Poaceae	Accepted
Sporobolus heterolepis (Gray) A.Gray.	Poaceae	Accepted
Sporobolus neglectus Nash.	Poaceae	Accepted
Sporobolus pungens (Schreb.) Kunth.	Poaceae	Accepted
Sporobolus vaginiflorus (Gray) Alph.Wood	Poaceae	Accepted
Stachys bullata Benth.	Lamiaceae	Accepted
Stachys byzantina 'Silver Carpet'	Lamiaceae	Variety of *Stachys byzantina* K.Koch.
Stachys byzantina K.Koch.	Lamiaceae	Accepted
Stachytarpheta jamaicensis (L.) Vahl	Verbenaceae	Accepted
Stapelia grandiflora Masson	Apocynaceae	Accepted
Stellaria media (L.) Vill.	Caryophyllaceae	Accepted
Stellaria graminea L.	Caryophyllaceae	Accepted
Stemodia lanata Ruiz & Pav.	Plantaginaceae	Accepted
Stenotaphrum secundatum (Walter) Kuntze	Poaceae	Accepted
Sternbergia lutea (L.) Ker Gawl. ex Spreng.	Amaryllidaceae	Accepted
Stipa bromoides (L.) Dörfl.	Poaceae	Accepted
Stipa capensis Thunb.	Poaceae	Accepted
Stipa comata Trin. & Rupr.	Poaceae	Accepted
Stipa neesiana Trin. & Rupr.	Poaceae	Synonym of *Nassella neesiana* (Trin. & Rupr.) Barkworth

Stipa pulchra	Poaceae	Synonym of *Nassella pulchra* (Hitchc.) Barkworth.
Stipa purpurea Griseb.	Poaceae	Accepted
Stipa tenuissima	Poaceae	Synonym of *Nassella tenuissima* (Trin.) Barkworth.
Strelitzia reginae Banks	Strelitziaceae	Accepted
Streptanthus cordatus Nutt.	Brassicaceae	Accepted
Struchium sparganophorum (L.) Kuntze.	Compositae	Accepted
Stylidium graminifolium	Stylidiaceae	Unresolved
Stylosanthes biflora (L.) Britton & al.	Leguminosae	Accepted
Stypandra glauca R.Br.	Xanthorrhoeaceae	Accepted
Suaeda vera Forssk. ex J.F.Gmel.	Amaranthaceae	Accepted
Succisa pratensis Moench.	Caprifoliaceae	Accepted
Symphoricarpos albus (L.) S.F.Blake.	Caprifoliaceae	Accepted
Symphoricarpos albus var. laevigatus 'Tilden Park'	Caprifoliaceae	Accepted
Symphyotrichum albus	Compositae	No results
Symphyotrichum azureus	Compositae	No results
Symphyotrichum chilense (Nees) G.L.Nesom.	Compositae	Accepted
Symphyotrichum depauperatum (Fernald) G.L.Nesom.	Compositae	Accepted
Symphyotrichum ericoides (L.) G.L.Nesom.	Compositae	Accepted
Symphyotrichum laeve (L.) Á.Löve & D.Löve	Compositae	Accepted

Symphyotrichum lateriflorum (L.) Á.Löve & D.Löve.	Compositae	Accepted
Symphyotrichum macrophyllus	Compositae	No results
Symphyotrichum novae-angliae (L.) G.L.Nesom.	Compositae	Accepted
Symphyotrichum novi-belgii (L.) G.L.Nesom.	Compositae	Accepted
Symphyotrichum oblongifolium (Nutt.) G.L.Nesom.	Compositae	Accepted
Symphyotrichum oolentangiense (Riddell) G.L.Nesom.	Compositae	Accepted
Symphyotrichum pilosum (Willd.) G.L.Nesom.	Compositae	Accepted
Symphyotrichum sericeum (Vent.) G.L.Nesom.	Compositae	Accepted
Symphyotrichum subspicatum (Nees) G.L.Nesom.	Compositae	Accepted
Synelcosciadium carmeli Boiss.	Apiaceae	Accepted
Syringa vulgaris L.	Oleaceae	Accepted
Syzygium smithii (Poir.) Nied.	Myrtaceae	Accepted
Tagetes erecta L.	Compositae	Accepted
Tagetes lemmonii A.Gray.	Compositae	Accepted
Tagetes minuta L.	Compositae	Accepted
Talinum calycinum aureum	Talinaceae	No results
Talinum calycinum Engelm.	Talinaceae	Accepted
Talinum paniculatum (Jacq.) Gaertn.	Talinaceae	Accepted
Talinum parviflorum	Montiaceae	Synonym of *Phemeranthus parviflorus* (Nutt.) Kiger.
Talinum rugospermum	Montiaceae	Synonym of *Phemeranthus rugospermus* (Holz.) Kiger.

Tanacetum corymbosum (L.) Sch.Bip.	Compositae	Accepted
Tanacetum vulgare L.	Compositae	Accepted
Taraxacum buttleri Soest.	Compositae	Accepted
Taraxacum campylodes G.E.Haglund	Compositae	Accepted
Taraxacum officinale	Compositae	Synonym of *Taraxacum campylodes* G.E.Haglund.
Taraxacum section ruderalia	Compositae	No results
Tetragonia implexicoma (Miq.) Hook. f.	Aizoaceae	Accepted
Tetragonia tetragonioides (Pall.) Kuntze.	Aizoaceae	Accepted
Teucrium aroanium Orph. ex Boiss.	Lamiaceae	Accepted
Teucrium aureum Schreb.	Lamiaceae	Accepted
Teucrium brevifolium Schreb.	Lamiaceae	Accepted
Teucrium canadense L.	Lamiaceae	Accepted
Teucrium capitatum L.	Lamiaceae	Accepted
Teucrium chamaedrys L.	Lamiaceae	Accepted
Teucrium fruticans L.	Lamiaceae	Accepted
Teucrium graphaloides L'Hér.	Lamiaceae	Accepted
Teucrium montanum L.	Lamiaceae	Accepted
Teucrium scorodonia L.	Lamiaceae	Accepted
Thalia dealbata Fraser.	Marantaceae	Accepted
Thalictrum dioicum L.	Ranunculaceae	Accepted
Thalictrum kiusianum Nakai	Ranunculaceae	Unresolved
Thelesperma ambiguum	Compositae	Synonym of *Thelesperma megapotamicum var. ambiguum* (A.Gray) Shinners.
Thelesperma filifolium (Hook.) A.Gray.	Compositae	Accepted

Thelionema grande (C.T.White) R.J.F.Hend.	Xanthorrhoeaceae	Accepted
Themeda triandra Forssk.	Poaceae	Accepted
Thermopsis rhombifolia (Pursh) Richardson.	Leguminosae	Accepted
Thryptomene baeckeacea F.Muell.	Myrtaceae	Accepted
Thymbra capitata (L.) Cav.	Lamiaceae	Accepted
Thymelaea passerina (L.) Coss. & Germ.	Thymelaeaceae	Accepted
Thymus argenteus	Lamiaceae	No results
Thymus caespititius Brot.	Lamiaceae	Accepted
Thymus × citriodorus (Pers.) Schreb.	Lamiaceae	Accepted
Thymus mongolicus (Ronniger) Ronniger.	Lamiaceae	Accepted
Thymus neiceffi	Lamiaceae	Synonym of *Thymus leucotrichus subsp. neiceffii* (Degen & Urum.) Jalas.
Thymus longicaulis C.Presl	Lamiaceae	Accepted
Thymus praecox Opiz.	Lamiaceae	Accepted
Thymus pseudolanuginosus	Lamiaceae	Synonym of *Thymus praecox subsp. britannicus* (Ronniger) Holub.
Thymus pulegioides L.	Lamiaceae	Accepted
Thymus serphyllum	Lamiaceae	Synonym of *Thymus mongolicus* (Ronniger) Ronniger.
Thymus serpyllum L.	Lamiaceae	Accepted
Thymus vulgaris L.	Lamiaceae	Accepted
Tidestromia oblongifolia	Amaranthaceae	Synonym of *Tidestromia suffruticosa var. oblongifolia* (S. Watson)

		Sánchez-del Pino & Flores Olvera.
Tillandsia recurvifolia Hook.	Bromeliaceae	Accepted
Tillandsia usneoides (L.) L.	Bromeliaceae	Accepted
Tilia cordata Mill.	Malvaceae	Accepted
Tordylium carmeli	Apiaceae	Unresolved
Torenia fournieri Linden ex E. Fourn.	Linderniaceae	Accepted
Torilis japônica (Houtt.) DC.	Apiaceae	Accepted
Torilis nodosa (L.) Gaertn.	Apiaceae	Accepted
Townsendia eximia A.Gray.	Compositae	Accepted
Trachelospermum jasminoides (Lindl.) Lem.	Apocynaceae	Accepted
Tradescantia bracteata Small ex Britton.	Commelinaceae	Accepted
Tradescantia occidentalis (Britton) Smyth	Commelinaceae	Accepted
Tradescantia ohiensis Raf.	Commelinaceae	Accepted
Tradescantia pallida (Rose) D.R.Hunt.	Commelinaceae	Accepted
Tradescantia tharpii E.S.Anderson & Woodson.	Commelinaceae	Accepted
Tradescantia zebrina Bosse.	Commelinaceae	Accepted
Tragopogon pratensis L.	Compositae	Accepted
Trichloris crinita (Lag.) Parodi	Poaceae	Accepted
Trichostema brachiatum L.	Lamiaceae	Accepted
Tricoryne elatior R.Br.	Xanthorrhoeaceae	Accepted
Tridax procumbens (L.) L.	Compositae	Accepted
Tridens flavus (L.) Hitchc.	Poaceae	Accepted
Trifolium alpinum L.	Leguminosae	Accepted
Trifolium angustifolium L.	Leguminosae	Accepted
Trifolium arvense L.	Leguminosae	Accepted

Trifolium campestre Schreb.	Leguminosae	Accepted
Trifolium dubium Sibth.	Leguminosae	Accepted
Trifolium fragiferum L.	Leguminosae	Accepted
Trifolium hybridum L.	Leguminosae	Accepted
Trifolium incarnatum L.	Leguminosae	Accepted
Trifolium medium L.	Leguminosae	Accepted
Trifolium palaestinum Boiss.	Leguminosae	Accepted
Trifolium pratense L.	Leguminosae	Accepted
Trifolium purpureum Loisel.	Leguminosae	Accepted
Trifolium repens L.	Leguminosae	Accepted
Trifolium stellatum L.	Leguminosae	Accepted
Trinia glauca (L.) Dumort.	Apiaceae	Accepted
Triodanis perfoliata (L.) Nieuwl.	Campanulaceae	Accepted
Tripleurospermum inodorum (L.) Sch.Bip.	Compositae	Accepted
Tripleurospermum maritimum (L.) W.D.J.Koch.	Compositae	Accepted
Trisetum flavescens (L.) P.Beauv.	Poaceae	Accepted
Tristellateia australasiae A. Rich.	Malpighiaceae	Accepted
Triticum aestivum L.	Poaceae	Accepted
Triticum dicoccoides (Körn. ex Asch. & Graebn.) Schweinf.	Poaceae	Accepted
Tuberaria guttata (L.) Fourr.	Cistaceae	Accepted
Tulbaghia violacea Harv.	Amaryllidaceae	Accepted
Tulipa bakeri	Liliaceae	Synonym of *Tulipa saxatilis subsp. bakeri* (A.D.Hall) Zonn.
Tulipa clusiana var. chrysantha	Liliaceae	Synonym of *Tulipa clusiana* DC.
Tulipa hageri 'Splendens'	Liliaceae	Variety of *Tulipa hageri* Heldr.
Tulipa humilis Herb.	Liliaceae	Accepted
Tulipa kolpakowskiana Regel.	Liliaceae	Accepted

Tulipa linifolia Regel.	Liliaceae	Accepted
Tulipa praestans 'Fusilier'	Liliaceae	Variety of *Tulipa praestans* H.B.May
Tulipa saxatilis Sieber ex Spreng.	Liliaceae	Accepted
Tulipa tarda Stapf.	Liliaceae	Accepted
Tulipa turkestanica (Regel) Regel.	Liliaceae	Accepted
Tulipa urumiensis	Liliaceae	Synonym of *Tulipa tarda* Stapf
Tussilago farfara L.	Compositae	Accepted
Typha angustifolia L.	Typhaceae	Accepted
Ulmus americana L.	Ulmaceae	Accepted
Ulmus glabra Huds.	Ulmaceae	Accepted
Ulmus minor Mill.	Ulmaceae	Accepted
Ulmus parvifolia Jacq.	Ulmaceae	Accepted
Ulmus pumila L.	Ulmaceae	Accepted
Uncinia rubra Colenso ex Boott	Cyperaceae	Accepted
Urtica dioica L.	Urticaceae	Accepted
Vaccinium angustifolium Aiton.	Ericaceae	Accepted
Vaccinium macrocarpon Aiton.	Ericaceae	Accepted
Vaccinium vitis-idaea L.	Ericaceae	Accepted
Valerianella locusta (L.) Laterr.	Caprifoliaceae	Accepted
Vella spinosa Boiss.	Brassicaceae	Accepted
Velleia paradoxa R.Br.	Goodeniaceae	Unresolved
Vemonia glauca (L.) Willd.	Compositae	Accepted
Verbascum blattaria L.	Scrophulariaceae	Accepted
Verbascum lychnitis L.	Scrophulariaceae	Accepted
Verbascum nigrum L.	Scrophulariaceae	Accepted
Verbascum phoeniceum L.	Scrophulariaceae	Accepted
Verbascum thapsus L.	Scrophulariaceae	Accepted
Verbena × hybrida	Verbenaceae	Unresolved
Verbena canadensis	Verbenaceae	Synonym of *Glandularia canadensis* (L.) Small.

Verbena hastata L.	Verbenaceae	Accepted
Verbena hybrida Groenl. & Rumpler	Verbenaceae	Accepted
Verbena simplex Lehm.	Verbenaceae	Accepted
Verbena stricta Vent.	Verbenaceae	Accepted
Vernonia cinerea var. cinerea	Compositae	Unresolved
Veronica arvensis L.	Plantaginaceae	Accepted
Veronica chamaedrys L.	Plantaginaceae	Accepted
Veronica gracilis R.Br.	Plantaginaceae	Unresolved
Veronica liwanensis	Plantaginaceae	Unresolved
Veronica longifolia L.	Plantaginaceae	Accepted
Veronica officinalis L.	Plantaginaceae	Accepted
Veronica pectinata L.	Plantaginaceae	Accepted
Veronica perfoliata R.Br.	Plantaginaceae	Accepted
Veronica persica Poir.	Plantaginaceae	Accepted
Veronica prostrata L.	Plantaginaceae	Accepted
Veronica spicata L.	Plantaginaceae	Accepted
Veronica thymoides	Plantaginaceae	Unresolved
Veronica whitleyi	Plantaginaceae	No results
Veronicastrum virginicum (L.) Farw.	Plantaginaceae	Accepted
Verticordia monadelpha Turcz.	Myrtaceae	Accepted
Vicia cracca L.	Leguminosae	Accepted
Vicia faba L.	Leguminosae	Accepted
Vicia sativa L.	Leguminosae	Accepted
Vicia sepium L.	Leguminosae	Accepted
Vicia hirsuta (L.) Gray	Leguminosae	Accepted
Vinca major L.	Apocynaceae	Accepted
Vinca minor L.	Apocynaceae	Accepted
Viola arvensis Murray.	Violaceae	Accepted
Viola canina L.	Violaceae	Accepted
Viola egglestonii	Violaceae	Synonym of *Viola septemloba subsp. egglestonii* (Brainerd) L.E. McKinney.

Viola inconspicua Blume.	Violaceae	Accepted
Viola odorata L.	Violaceae	Accepted
Viola pedata L.	Violaceae	Accepted
Viola pedatifida G. Don.	Violaceae	Accepted
Viola praemorsa Douglas	Violaceae	Accepted
Viola reichenbachiana Jord. ex Boreau.	Violaceae	Accepted
Viola sagittata Aiton.	Violaceae	Accepted
Viola tricolor L.	Violaceae	Accepted
Viola × wittrockiana Gams	Violaceae	Unresolved
Vitis vinifera L.	Vitaceae	Accepted
Vittadinia cuneata DC.	Compositae	Accepted
Vittadinia muelleri N.T.Burb.	Compositae	Accepted
Vulpia ciliata Dumort.	Poaceae	Accepted
Vulpia geniculata (L.) Link	Poaceae	Accepted
Vulpia myuros (L.) C.C.Gmel.	Poaceae	Accepted
Wahlenbergia communis Carolin	Campanulaceae	Synonym of *Wahlenbergia capillaris* (Lodd.) G.Don
Wahlenbergia gracilis (G.Forst.) A.DC.	Campanulaceae	Accepted
Wahlenbergia luteola P.J.Sm.	Campanulaceae	Accepted
Wahlenbergia stricta (R.Br.) Sweet.	Campanulaceae	Accepted
Wedelia trilobata	Compositae	Synonym of *Sphagneticola trilobata* (L.) Pruski.
Wisteria sinensis (Sims) Sweet.	Leguminosae	Accepted
Woodwardia fimbriata Sm.	Blechnaceae	Accepted
Xanthorrhoea minor R.Br.	Xanthorrhoeaceae	Accepted
Xerochrysum bracteatum (Vent.) Tzvelev.	Compositae	Accepted

Xerochrysum viscosum (Sieber ex DC.) R.J.Bayer	Compositae	Accepted
Youngia japonica (L.) DC.	Compositae	Accepted
Yucca glauca Nutt.	Asparagaceae	Accepted
Zea mays L.	Poaceae	Accepted
Zebrina pendula	Commelinaceae	Synonym of *Tradescantia zebrina var. zebrina*
Zenith zoysia grass	Poaceae	No results
Zephyranthes candida (Lindl.) Herb.	Amaryllidaceae	Accepted
Zeuxine strateumatica (L.) Schltr.	Orchidaceae	Accepted
Zingiber officinale Roscoe	Zingiberaceae	Accepted
Zinnia elegans L.	Compositae	Accepted
Zinnia peruviana (L.) L.	Compositae	Accepted
Zizia aurea Koch.	Apiaceae	Accepted
Ziziphora bungeana	Lamiaceae	Synoym of *Ziziphora clinopodioides subsp. bungeana* (Juz.) Rech.f.
Zoysia japonica Steud.	Poaceae	Accepted
Zoysia matrella (L.) Merr.	Poaceae	Accepted
Zoysia matrella ‘Zeon’	Poaceae	Variety of *Zoysia matrella* (L.) Merr.
Zoysia tenuifolia Thiele.	Poaceae	Accepted
Zygophyllum billardierei	Zygophyllaceae	Unresolved
