# Supplementary material for: Life at the Top: Extensive Green Roof Plant Species and Their Traits for Urban Use
Source: Plants (Basel). 2025 Feb 27;14(5):735. doi: 10.3390/plants14050735 (PMC11902263; doi:10.3390/plants14050735)
Supplement: Supplementary file 1 [file plants-14-00735-s001.zip › S2 - full reference list.pdf]

Supplementary Information SI2 – Full reference list

1. Agra H, Klein T, Vasl A, Kadas G, Blaustein L (2017a) Measuring the effect of plant-community composition on carbon fixation on green roofs. Urban Forestry and Urban Greening http://dx.doi.org/10.1016/j.ufug.2017.03.003

2. Agra H, Klein T, Vasl A, Shalom H, Kadas G, Blaustein L (2017b) Sedum-dominated green-roofs in a semi-arid region increase CO_2 concentrations during the dry season. Science of the Total Environment 584–585, 1147–1151. https://doi.org/10.1016/j.scitotenv.2017.01.176

3. Agra H, Solodar A, Bawaba O, Levya S, Kadas G, Blaustein L, Greenbaum N (2018) Comparing grey water versus tap water and coal ash versus perlite on growth of two plant species on green roofs. Science of the Total Environment 633, 1272–1279. https://doi.org/10.1016/j.scitotenv.2018.03.291

4. Aguiar AC, Robinson SA, French K (2019) Friends with benefits: The effects of vegetative shading on plant survival in a green roof environment. PLOS ONE | https://doi.org/10.1371/journal.pone.0225078

5. Ahmed S, Buckley S, Stratton AE, Asefaha F, Butler C, Reynolds M, Orians C (2017) Sedum Groundcover Variably Enhances Performance and Phenolic Concentrations of Perennial Culinary Herbs in an Urban Edible Green Roof. Agroecology and Sustainable Food Systems, DOI: 10.1080/21683565.2017.1279703

6. Aitkenhead-Peterson JA, Dvorak BD, Volder A, Stanley NC (2011) Chemistry of growth medium and leachate from green roof systems in south-central Texas. Urban Ecosyst 14:17–33. DOI:10.1007/s11252-010-0137-4

7. Aloisio JM, Tuininga AR, Lewis JD (2016) Crop species selection effects on stormwater runoff and ediblebiomass in an agricultural green roof microcosm. Ecological Engineering 88, 20–27. DOI:10.1016/j.ecoleng.2015.12.022

8. Aloisio JM, Palmer MI, Tuininga AR, Lewis JD (2019) Plant Colonization of Green Roofs Is Affected by Composition of Established Native Plant Communities. Frontiers in Ecology and Evolution, Volume 6, Article 9. DOI:10.3389/fevo.2018.00238

9. Aloisio JM, Palmer MI, Tuininga AR, Lewis JD (2020) Introduced and native plant species composition of vacant unmanaged green roofs in New York City. Urban Ecosystems 23:1227–1238. DOI:10.1007/s11252-020-00992-6

10. Alomía-Lucero J, Castro-Bedriñana J, Chirinos-Peinado D (2020) Rooftop Urban Agriculture Model with Two Tomato Varieties (Lycopersicum esculentum Mill) and Toppings in the High Jungle – Peru. Advances in Science, Technology and Engineering Systems Journal Vol. 5, No. 1, 446-450. DOI: 10.25046/aj050157

11. Amir M, Katoha Y, Katsurayama H, Koganei M, Mizunuma M (2018) Effects of convection heat transfer on Sunagoke moss green roof: A laboratory study. Energy and Buildings 158, 1417–1428. DOI:10.1016/j.enbuild.2017.11.043

12. Anderson M, Lambrinos J, Schroll E (2010) The potential value of mosses for stormwater management in urban environments. Urban Ecosyst 13: 319–332. DOI:10.1007/s11252-010-0121-z

13. Andrés-Doménech I, Perales-Momparler S, Morales-Torres A, Escuder-Bueno I (2018) Hydrological Performance of Green Roofs at Building and City Scales under Mediterranean Conditions. Sustainability 10, 3105. doi:10.3390/su10093105

14. Aprile S, Tuttolomondo T, Gennaro MC, Leto C, La Bella S, Licata M (2020) Effects of plant density and cutting-type on rooting and growth of an extensive green roof of Sedum sediforme (Jacq.) Pau in a Mediterranean environment. Scientia Horticulturae 262, 109091. DOI : 10.1016/j.scienta.2019.109091

15. Arbid Y, Richard C, Sleiman M (2021) Towards an experimental approach for measuring the removal of urban air pollutants by green roofs. Building and Environment 205, 108286. DOI: 10.1016/j.buildenv.2021.108286 . hal-03329684f

16. Arbid Y, Sleiman M, Richard C (2022) Photochemical interactions between pesticides and plant volatiles. Science of the Total Envronment 807, 150716. https://doi.org/10.1016/j.scitotenv.2021.150716

17. Arboit NKS, Tassi R, Liberalesso T, Ceconi DE, Piccili DGA (2021) Green roof evapotranspiration rates and stormwater control under subtropical climate: a case study in Brazil. Revista Brasileira de Recursos Hídricos Brazilian Journal of Water Resources Versão On-line ISSN 2318-0331 RBRH, Porto Alegre, v. 26, e32, Scientific/Technical Article. https://doi.org/10.1590/2318-0331.262120210089

18. A'saf TS, Al-Ajlouni MG, Ayad JY, Othman YA, Hilaire RS (2020) Performance of six different soilless green roof substrates for the Mediterranean region. Science of the Total Environment 730, 139182. DOI: 10.1016/j.scitotenv.2020.139182

19. Askari, S.H.; De-Ville, S.; Hathway, E.A.; Stovin, V. Estimating Evapotranspiration from Commonly Occurring Urban Plant Species Using Porometry and Canopy Stomatal Conductance. Water 2021, 13, 2262. https://doi.org/10.3390/w13162262

20. Azeñas V, Cuxart J, Picos R, Medrano H, Simó G, López-Grifol A, Gulías J (2018a) Thermal regulation capacity of a green roof system in the Mediterranean region: The effects of vegetation and irrigation level. Energy & Buildings 164, 226–238. DOI:10.1016/j.enbuild.2018.01.010

21. Azeñas V, Janner I, Medrano H, Gulías J (2018b) Performance evaluation of five Mediterranean species to optimize ecosystem services of green roofs under water-

limited conditions. Journal of Environmental Management 212, 236-247. DOI: 10.1016/j.jenvman.2018.02.021

22. Azeñas V, Janner I, Medrano H, Gulías J (2019) Evaluating the establishment performance of six native perennial Mediterranean species for use in extensive green roofs under water-limiting conditions. Urban Forestry & Urban Greening 41, 158–169. DOI:10.1016/j.ufug.2019.04.002

23. Baraldi R, Neri L, Costa F, Facini O, Rapparini F, Carriero G (2019) Ecophysiological and micromorphological characterization of green roof vegetation for urban mitigation. Urban Forestry & Urban Greening, Volume 37, Pages 24-32. DOI:10.1016/j.ufug.2018.03.00

24. Barbaro LA, Sisaro D, Stancanelli S, Soto MS (2021) Polvo de ladrillo como sustrato para techos verdes extensivos. Chilean J. Agric. Anim. Sci., ex Agro-Ciencia, 37(1):81-91. DOI:10.29393/CHJAAS37-9PLLB40009

25. Barker KJ & Lubell JD (2012) Effects of Species Proportions and Fertility on Sedum Green Roof Modules. HortTechnology, 2, 196-200. https://doi.org/10.21273/HORTTECH.22.2.196

26. Barozzi B, Bellazzi A, Pollastro MC (2016) The Energy Impact in Buildings of Vegetative Solutions for Extensive Green Roofs in Temperate Climates. Buildings 6, 33; doi:10.3390/buildings6030033

27. Barr CM; Gallagher PM, Wadzuk BM, Welker AL (2017) Water Quality Impacts of Green Roofs Compared with Other Vegetated Sites. American Society of Civil Engineers. Journal of Sustainable Water Built Environment 1. DOI: 10.1061/JSWBAY.0000825

28. Baryła A, Gnatowski T, Karczmarczyk A, Szatyłowicz J (2019) Changes in Temperature and Moisture Content of an Extensive-Type Green Roof. Sustainability 11, 2498; doi:10.3390/su11092498

29. Bates AJ, Sadler JP, Mackay R (2013) Vegetation development over four years on two green roofs in the UK. Urban Forestry & Urban Greening 12, 98– 108. DOI:10.1016/j.ufug.2012.12.003

30. Bates AJ, Sadler JP, Greswell RB, Mackay R (2015) Effects of varying organic matter content on the development of green roof vegetation: A six year experimente. Ecological Engineering 82, 301–310. DOI:10.1016/j.ecoleng.2015.04.102

31. Beecham S & Razzaghmanesh M (2015) Water quality and quantity investigation of green roofs in a dry climate. Water research 70, 370-384. DOI:10.1016/j.watres.2014.12.015

32. Benvenuti S & Bacci D (2010) Initial agronomic performances of Mediterranean xerophytes in simulated dry green roofs. Urban Ecosyst 13:349–363. DOI:10.1007/s11252-010-0124-9

33. Benvenuti S (2014) Wildflower green roofs for urban landscaping, ecological sustainability and biodiversity. Landscape and Urban Planning, Volume 124, 151-161. DOI:10.1016/j.landurbplan.2014.01.004

34. Berthon K, Thomas F, Baumann J, White R, Bekessy S, Encinas-Viso F (2023) Floral resources encourage colonisation and use of green roofs by invertebrates. Urban Ecosystems. https://doi.org/10.1007/s11252-023-01392-2

35. Bevilacqua P, Coma J, Perez G, Chocarro C, Juarez A, Sole C, Simone MD, Cabeza LF (2015) Plant cover and floristic composition effect on thermal behaviour of extensive green roofs. Building and Environment 92, 305-316. DOI:10.1016/j.buildenv.2015.04.026

36. BlanusaT, Monteiro MMV, Fantozzi F, Vysini E, Li Y, Cameron RWF (2013) Alternatives to Sedum on green roofs: Can broad leaf perennial plants offer better 'cooling service'? Building and Environment 59, 99-106. doi: https://doi.org/10.1016/j.buildenv.2012.08.011

37. Bortolini L, Bettella F, Zanin G (2021) Hydrological Behaviour of Extensive Green Roofs with Native Plants in the Humid Subtropical Climate Context. Water, 13, 44. https://doi.org/10.3390/w13010044

38. Bousselot JM, Klett JE, Koski RD (2010) Extensive Green Roof Species Evaluations Using Digital Image Analysis. Hortscience 45(8):1288–1292. DOI:10.21273/HORTSCI.45.8.1288

39. Bousselot JM, Klett JE, Koski RD (2011) Moisture Content of Extensive Green Roof Substrate and Growth Response of 15 Temperate Plant Species during Dry Down. Hortscience 46(3):518–522. https://doi.org/10.21273/HORTSCI.46.3.518

40. Brandão C, Cameira MR, Valente F, Carvalho RC, Paço TA (2017) Wet season hydrological performance of green roofs using native species under Mediterranean climate. Ecological Engineering 102, 596–611. https://doi.org/10.1016/j.ecoleng.2017.02.025

41. Brown C, Lundholm J (2015) Microclimate and substrate depth influence green roof plant community dynamics. Landscape and Urban Planning 143, 134–142. https://doi.org/10.1016/j.landurbplan.2015.07.009

42. Brunetti G, Papagrigoriou IA, Simunek J, Stumpp C (2021) Green Roofs for domestic wastewater treatment: Experimental and numerical analysis of nitrogen turnover. Journal of Hydrology 603, 127132. https://doi.org/10.1016/j.jhydrol.2021.127132

43. Buccola N & Spolek G (2011) A Pilot-Scale Evaluation of Greenroof Runoff Retention, Detention, and Quality. Water Air Soil Pollut 216: 83–92. DOI:10.1007/s11270-010-0516-8

44. Buckland-Nicks M, Heim A, Lundholm J (2016) Spatial environmental heterogeneity affects plant growth and thermal performance on a green roof. Science of the Total Environment 553, 20–31. DOI:10.1016/j.scitotenv.2016.02.063

45. Butcher CL, Rubin BY, Anderson SL, Lewis JD (2020) Pollen dispersal patterns differ among sites for a wind-pollinated species and an insect-pollinated species. American Journal of Botany 107(11): 1504–1517, 2020; http://www.wileyonlinelibrary.com/journal/AJB

46. Butler C & Orians CM (2011) Sedum cools soil and can improve neighboring plant performance during water deficit on a green roof. Ecological Engineering 37, 1796– 1803. DOI:10.1016/j.ecoleng.2011.06.025

47. Cáceres N, Imhof L, Suárez M, Hick EC, Galetto L (2018) Assessing native germplasm for extensive green roof systems of semiarid regions. Ornamental Horticulture V 24 N4, 466-476. https://doi.org/10.14295/oh.v24i4.1225

48. Cakar H, Saracoglu OA, Akat H, Kilic CC, Adanacioglu H (2023) the potential for using different substrates in green roofs. Journal of Environmental Engineering and Landscape Management Volume 31 Issue 1: 44–51 https://doi.org/10.3846/jeelm.2023.18487

49. California Academy of Science (2014) Native Plant Species on the Living Roof. https://www.calacademy.org/sites/default/files/assets/docs/pdf/roof_plant_list_sept2014.pdf

50. Calviño AA, Tavella J, Beccacece HM, Estallo EL, Fabián D, Moreno ML, Salvo A, Fenoglio MS (2023) The native - exotic plant choice in green roof design: Using a multicriteria decision framework to select plant tolerant species that foster beneficial arthropods. Ecological Engineering 187, 106871. https://doi.org/10.1016/j.ecoleng.2022.106871

51. Cao J, Hu S, Dong Q, Liu L, Wang Z (2019) Green roof cooling contributed by plant species with different photosynthetic strategies. Energy & Buildings 195, 45–50. DOI:10.1016/j.enbuild.2019.04.046

52. Carmo-Silva AE, Francisco A, Powers SJ, Keys AJ, Ascensão L, Parry MAJ, Arrabaça MC (2009) Grasses Of Different C4 Subtypes Reveal Leaf Traits Related To Drought Tolerance In Their Natural Habitats: Changes In Structure, Water Potential, And Amino Acid Content. American Journal of Botany 96(7): 1222–1235. DOI: 10.3732/ajb.0800224

53. Carreño-Ortega A, do Paço TA, Díaz-Pérez M, Gómez-Galán M (2021) Lettuce Production under Mini-PV Modules Arranged in Patterned Designs. Agronomy, 11, 2554. https://doi.org/10.3390/ agronomy11122554

54. Cascone S, Gagliano A, Poli T, Sciuto G (2019) Thermal performance assessment of extensive green roofs investigating realistic vegetation-substrate configurations. Building Simulation volume 12, 379–393. DOI:10.1007/s12273-018-0488-y

55. Caser M, Demasi S, Mozzanini E, Chiavazza PM, Scariot V (2022) Germination Performances of 14 Wildflowers Screened for Shaping Urban Landscapes in Mountain Areas. Sustainability, 14, 2641. https://doi.org/10.3390/ su14052641

56. Catalano C, Marcenò C, Laudicina VA, Guarino R (2016) Thirty years unmanaged green roofs: Ecological research and design implications. Landscape and Urban Planning 149, 11–19. DOI:10.1016/j.landurbplan.2016.01.003

57. Chagolla-Aranda MA, Simá E, Xamán J, Álvarez G, Hernández-Pérez I, Téllez-Velázquez E (2017) Effect of irrigation on the experimental thermal performance of agreen roof in a semi-warm climate in Mexico. Energy and Buildings 154, 232–243. DOI:10.1016/j.enbuild.2017.08.082

58. Chai H, Tang Y, Su X, Wang W, Lu H, Shao Z, He Q (2018) Annual variation patterns of the effluent water quality from a green roof and the overall impacts of its structure. Environmental Science and Pollution Research https://doi.org/10.1007/s11356-018-3039-2

59. Charalambous K, Bruggeman A, Eliades M, Camera C, Vassiliou L (2019) Stormwater Retention and Reuse at the Residential Plot Level—Green Roof Experiment and Water Balance Computations for Long-Term Use in Cyprus. Water, 11, 1055; doi:10.3390/w11051055

60. Chemisana D & Lamnatou C (2014) Photovoltaic-green roofs: An experimental evaluation of system performance. Applied Energy 119, 246–256. DOI: 10.1016/j.apenergy.2013.12.027

61. Chen CF & Kang SF (2016) Effects of Substrates and Plant Species on Water Quality of Extensive Green Roofs. Applied Ecology and Environmental Research 14(2):77-91. DOI: http://dx.doi.org/10.15666/aeer/1402_077091

62. Chen CF, Kang SF, Lin JH (2018b) Effects of recycled glass and different substrate materials on the leachate quality and plant growth of green roofs. Ecological Engineering 112, 10–20. DOI:10.1016/j.ecoleng.2017.12.013

63. Cheng D, Zhang Q, Huang S (2020) Effects of capillary wicking irrigation on soil moisture, plant growth and surface temperature of green roof with rain storage. Water Supply I Press. doi: 10.2166/ws.2020.071

64. Chen H, Ma J, Wei J, Gong X, Yu X, Guo H, Zhao Y (2018a) Biochar increases plant growth and alters microbial communities via regulating the moisture and temperature of green roof substrates. Science of the Total Environment 635, 333–342. DOI: 10.1016/j.scitotenv.2018.04.127

65. Chen X, Jeong S, Zheng Y, Park H, Park CE, Joo J, Choi W, Chen X, Zheng C (2020) Evaluation of different roof materials for the mitigation of urban warming in a subtropical monsoon climate. American Geophysical Union. doi:10.1029/2019JD031972

66. Chen X, Liu R, Liu D, Xin X (2023) Analysis of Preferential Flow in Artificial Substrates with Sedum Roots for Green Roofs: Experiments and Modeling. Water 15, 914. https://doi.org/10.3390/w15050914

67. Chow MF, Abu Bakar MF, Sidek LM, Basri H (2018) The Effect of Green Roof Configurations on Runoff Retention Performance. Journal of Engineering and Applied Science 13 (3), 3183-3187. DOI:10.3923/jeasci.2018.3183.3187

68. Chow MF, Bakar MFA, Wong JK, Ling L (2021) Evapotranspiration Measurement and Estimation of Crop Coefficient for Native Plant Species of Green Roof in the Tropics. Water, 13, 1669. https://doi.org/10.3390/ w13121669

69. Chowdhury RK & Abaya JS (2018) An Experimental Study of Greywater Irrigated Green Roof Systems in an Arid Climate. Journal of Water Management Modeling 26:C437. https://doi.org/10.14796/JWMM.C437

70. Chu HH & Farrell C (2022) Fast plants have water-use and drought strategies that balance rainfall retention and drought survival on green roofs. Ecological Applications 32(1): e02486. 10.1002/eap. 2486

71. Cirkel DG, Voortman BR, van Veen T, Bartholomeus RP (2018) Evaporation from (Blue-)Green Roofs: Assessing the Benefits of a Storage and Capillary Irrigation System Based on Measurements and Modeling. Water 10, 1253; doi:10.3390/w10091253

72. Clark MJ & Zheng Y (2012) Evaluating Fertilizer Influence on Overwintering Survival and Growth of Sedum Species in a Fall-installed Green Roof. Hortscience 47(12):1775–1781. DOI:10.21273/HORTSCI.47.12.1775

73. Clark MJ & Zheng Y (2013) Plant Nutrition Requirements for an Installed Sedum-Vegetated Green Roof Module System: Effects of Fertilizer Rate and Type on Plant Growth and Leachate Nutrient Content. Hortscience 48(9):1173–1180. DOI:10.21273/HORTSCI.48.9.1173

74. Connelly M & Hodgson M (2013) Experimental investigation of the sound transmission of vegetated roofs. Applied Acoustics 74, 1136–1143. DOI:10.1016/j.apacoust.2013.04.003

75. Connelly M & Hodgson M (2015) Experimental investigation of the sound absorption characteristics of vegetated roofs, Building and Environment, doi: 10.1016/ j.buildenv.2015.04.023.

76. Contreras-Bejarano O & Villegas-González PA (2019) Green roofs for comprehensive water management: Case study in Chapinero, Colombia. Tecnología y ciencias del agua, ISSN 2007-2422, 10(5), 282-318. DOI: 10.24850/j-tyca-2019-05-11

77. Cotoz AP, Dan VS, Gocan TM, Andreica I, Rózsa S, Cantor M (2023) Sedum Growth Patterns under Different Pedoclimatic Conditions. Plants 12, 2739. https://doi.org/10.3390/plants12142739

78. Coutts AM, Daly E, Beringer J, Tapper NJ (2013) Assessing practical measures to reduce urban heat: Green and cool roofs. Building and Environment 70, 266-276. DOI:10.1016/j.buildenv.2013.08.021

79. Da Silva M, Najjar MK, Hammad AWA, Haddad A, Vazquez E (2020) Assessing the Retention Capacity of an Experimental Green Roof Prototype. Water 12, 90; doi:10.3390/w12010090

80. Dela Cruz J, Caya MV, Fajardo AD, Manzanilla MJ, Sabuco MR, Chummac A, Fke NBZ (2020) Greenroof Irrigation Using Evapotranspiration for Alfafa and Basil. International Conference on Humanoid, Nanotechnology, Information Technology, Communication and Control, Environment, and Management. Doi: 978-0-7381-1177-3/20/$31.00

81. Deng H & Jim CY (2016) Spontaneous plant colonization and bird visits of tropical extensive green roof. Urban Ecosystems volume 20, 337–352. DOI:10.1007/s11252-016-0596-3

82. Deska I, Mrowiec M, Ociepa E, Lewandowska A (2020) Influence of the Hydrogel Amendment on the Water Retention Capacity of Extensive Green Roof Models. Journal of Ecological Engineering Volume 21, Issue 1, pages 195–204 https://doi.org/10.12911/22998993/112763

83. Di Bonito R, Biagiotti D, Giagnacovo G, Canditelli M, Campiotti CA (2016) Use of compost as amendment for soilless substrates of plants in green roof installations. Acta Hortic. 1146. ISHS DOI 10.17660/ActaHortic.2016.1146.19

84. Dirks I, Raviv B, Shelef O, Hill A, Eppel A, Aidoo MK, Hoefgen B, Rapaport T, Gil H, Geta E, Kochavi A, Cohen I, Rachmilevitch S (2016) Green roofs: what can we learn from desert plants? Israel Journal of Ecology & Evolution, Vol. 62, Nos. 1-2, 58-67, http://dx.doi.org/10.1080/15659801.2016.1140619

85. Dromgold JR, Threlfall CG, Norton BA, Williams NSG (2020) Green roof and ground-level invertebrate communities are similar and are driven by building height and landscape context. Journal of Urban Ecology, 1–9. https://doi.org/10.1093/jue/juz024

86. Du P, Arndt SK, Farrell C (2018) Relationships between plant drought response, traits, and climate of origin for green roof plant selection. Ecological Applications, 28(7), 1752–1761. DOI: 10.1002/eap.1782

87. Du P, Arndt SK, Farrell C (2019a) Is plant survival on green roofs related to their drought response, water use or climate of origin? Science of the Total Environment 667, 25–32. https://doi.org/10.1016/j.scitotenv.2019.02.349

88. Du P, Arndt SK, Farrell C (2019b) Can the turgor loss point be used to assess drought response to select plants for green roofs in hot and dry climates? Plant Soil, 441:399–408. DOI:10.1007/s11104-019-04133-7

89. Durà VB, Meseguer E, Crespo CH, Monerris MM, Doménech IA, Santamalia MER (2023) Contribution of green roofs to urban arthropod biodiversity in a Mediterranean climate: A case study in València, Spain. Building and Environment 228, 109865. https://doi.org/10.1016/j.buildenv.2022.109865

90. Dusza Y, Barot S, Kraepiel Y, Lata JC, Abbadie L, Raynaud X (2016) Multifunctionality is affected by interactions between green roof plant species, substrate depth, and substrate type. Ecology and Evolution Volume 7, Issue 7, 2357-2369. https://doi.org/10.1002/ece3.2691

91. Dusza Y, Kraepiel Y, Abbadie L, Barot S, Carmignac D, Dajoz I, Gendreau E, Lata JC, Meriguet J, Motard E, Raynaud X (2020) Plant-pollinator interactions on green roofs are mediated by substrate characteristics and plant community composition. https://www.elsevier.com/open-access/userlicense/1.0/

92. Dvorak BD & Volder A (2013a) Plant establishment on unirrigated green roof modules in a subtropical climate. AoB PLANTS 5: pls049; doi:10.1093/aobpla/pls049

93. Dvorak BD & Volder A (2013b) Rooftop temperature reduction from unirrigated modular green roofs in south-central Texas. Urban Forestry & Urban Greening 12, 28– 35. DOI:10.1016/j.ufug.2012.05.004

94. Eksi M & Rowe DB (2016) Green roof substrates: Effect of recycled crushed porcelain andfoamed glass on plant growth and water retention. Urban Forestry & Urban Greening 20, 81–88. DOI:10.1016/j.ufug.2016.08.008

95. Eksi M, Rowe DB, Fernández-Canero R, Cregg BM (2015) Effect of substrate compost percentage on green roof vegetable production. Urban Forestry & Urban Greening 14, 315–322. DOI:10.1016/j.ufug.2015.03.006

96. Eksi M, Rowe DB, Wichman IS, Andresen JA (2017) Effect of substrate depth, vegetation type, and season on green roofthermal properties. Energy and Buildings 145, 174–187. DOI:10.1016/j.enbuild.2017.04.017

97. Eksi M, Sevgi O, Akburak S, Yurtseven H, Esin I (2020) Assessment of recycled or locally available materials as green roof substrates. Ecological Engineering 156 (2020) 105966. DOI:10.1016/j.ecoleng.2020.105966

98. Elansary HO (2017) Green roof *Petunia, Ageratum*, and *Mentha* responses to water stress, seaweeds, and trinexapac-ethyl treatments. Acta Physiol Plant, 39:145 DOI 10.1007/s11738-017-2444-3

99. Erwin J & Hensley J (2019) Plants with Horticultural and Ecological Attributes for Green Roofs in a Cool, Dry Climate. Hortscience 54(10):1703–1711. DOI:10.21273/HORTSCI13893-19

100.Escalona A, Salas MC, Coutinho CS, Guzmán M (2013) How does salinity affect mineral ion relations and growth of Lobelia erinus for use in urban landscaping? Journal of Food, Agriculture & Environment Vol.11 (2): 854-858. https://www.researchgate.net/publication/257416170_How_does_salinity_affect_mineral_ion_relations_and_growth_of_Lobelia_erinus_for_use_in_urban_landscaping

101.Escalona A, Salas-Sanjuán MC, Dos Santos C, Guzmán M (2014) Efecto de aguas salinas sobre el crecimiento, concentración y relaciones de iones en Zinnia elegans y Tagetes erecta para su uso en jardinería urbana. ITEA, Vol. 110 (4), 325-334. DOI:10.12706/itea.2014.020

102.Esfahani RE, Paço TA, Martins D, Arsenio P (2022) Increasing the resistance of Mediterranean extensive green roofs by using native plants from old roofs and walls. Ecological Engineering 178, 106576. https://doi.org/10.1016/j.ecoleng.2022.106576

103.Fabian D, González E, Dominguez MVS, Salvo A, Fenoglio MS (2021) Towards the design of biodiverse green roofs in Argentina: Assessing key elements for different functional groups of arthropods. Urban Forestry & Urban Greening 61, 127107. https://doi.org/10.1016/j.ufug.2021.127107

104.Fai CM, Abu Bakar MF, bin Roslan MAA, Fadzailah FAB, bin Idrus MFZ, Ismail NFB, Sidek LM, Basri H (2015) Hydrological Performance Of Native Plant Species Within Extensive Green Roof System In Malaysia. ARPN Journal of Engineering and Applied Sciences, Vol 10, N 15.

https://www.researchgate.net/publication/282939081_Hydrological_performance_of_native_plant_species_within_extensive_green_roof_system_in_Malaysia

105.Fang CF (2010) Rainwater retention capacity of green roofs in subtropical monsoonal climatic regions: a case study of Taiwan. WIT Transactions on Ecology and the Environment, Vol 138. doi:10.2495/DN100211

106.Farrell C, Mitchell RE, Szota C, Rayner JP, Williams NSG (2012) Green roofs for hot and dry climates: Interacting effects of plant water use, succulence and substrate. Ecological Engineering 49, 270– 276. https://doi.org/10.1016/j.ecoleng.2012.08.036

107.Farrell C, Szota C, Williams NSG, Arndt SK (2013a) High water users can be drought tolerant: using physiological traits for green roof plant selection. Plant Soil DOI 10.1007/s11104-013-1725-x

108.Farrell C, Ang XQ, Rayner JP (2013b) Water-retention additives increase plant available water in green roof substrates. Ecological Engineering 52, 112– 118. DOI:10.1016/j.ecoleng.2012.12.098

109.Fenoglio MS, Gonzalez E, Tavella J, Beccacece H, Moreno ML, Fabian D, Salvo A, Estallo EL, Calvino A (2023) Native plants on experimental urban green roofs support higher community-level insect abundance than exotics. Urban Forestry & Urban Greening 86, 128039. https://doi.org/10.1016/j.ufug.2023.128039

110.Ferrans P, Rey CV, Pérez G, Rodríguez JP, Díaz-Granados M (2018) Effect of Green Roof Configuration and Hydrological Variables on Runoff Water Quantity and Quality. Water 10, 960. doi:10.3390/w10070960

111.Ferrante P, Gennusa ML, Peri G, Rizzo G, Scaccianoce G (2016) Vegetation growth parameters and leaf temperature: Experimental results from a six plots

green roofs' system. Energy, Volume 115, Part 3, 1723-1732. DOI: 10.1016/j.energy.2016.07.085

112.Fezzioui N & Benaichata M (2021) Green roofs under hot and dry climate in south-west of Algeria: Study of the implementation conditions. ITU AZ, Vol 18 (2), 319-330. doi: 10.5505/itujfa.2021.04657

113.Franco BM, Andres CM, Konrad J, Tassia R, Liberalesso T (2019) Avaliação de escoamento pluvial em módulos de telhados verdes com diferentes substratos. Acta Brasiliensis 3(2): 69-73. http://dx.doi.org/10.22571/10.22571/2526-4338184

114.Franzaring J, Steffan L, Ansel W, Walker R, Fangmeier A (2016) Water retention, wash-out, substrate and surface temperatures ofextensive green roof mesocosms—Results from a two year study in SW-Germany. Ecological Engineering 94, 503–515. https://doi.org/10.1016/j.ecoleng.2016.06.021

115.Gabrych M, Kotze DJ, Lehvävirta S (2016) Substrate depth and roof age strongly affect plant abundanceson sedum-moss and meadow green roofs in Helsinki, Finland. Ecological Engineering 86, 95–104. DOI:10.1016/j.ecoleng.2015.10.022

116.Galarza-Molina S, Torres A, Rengifo P, Puentes A, Carcamo-Hernandez E, Mendez-Fajardo S, Devia C (2016) The benefits of an eco-productive green roof in Bogota, Colombia. Indoor and Built Environment 0(0), 1–9. https://doi.org/10.1177/1420326X16665896

117.Gallardo NP, Rogério A, Neves GF, Vecchia FA, Roriz VF (2018) Thermal response to cold in buildings with green covers for tropical climate. Green facades and green roofs. Revista Ingeniería de Construcción RIC Vol 33 Nº1. DOI: 10.4067/S0718-50732018000100015

118.Garg A, Hazra B, Zhu H, Wend Y (2019) A simplified probabilistic analysis of water content and wilting in soil vegetated with non-crop species. Catena 175, 123–131. DOI:10.1016/j.catena.2018.12.016

119.Getter KL & Rowe DB (2009) Substrate Depth Influences Sedum Plant Community on a Green Roof. Hortscience 44(2):401–407. DOI:10.21273/HORTSCI.44.2.401

120.Getter KL, Rowe DB, Cregg BM (2009a) Solar radiation intensity influences extensive green roof plant communities. Urban Forestry & Urban Greening 8 269–281. DOI:10.1016/j.ufug.2009.06.005

121.Getter KL, Rowe DB, Robertson GF, Cregg BM, Andresen JA (2009b) Carbon Sequestration Potential of Extensive Green Roofs. Environmental Science Technology, 43, 7564–7570. doi: 10.1021/es901539x

122.Gerzhova N, Blanchet P, Dagenais C, Ménard S, Côté J (2020) Flammability Characteristics of Green Roofs. Buildings 10, 126. doi:10.3390/buildings10070126

123.Gioannini R, Al-Ajlouni M, Kile R, VanLeeuwen D, St. Hilaire R (2018) Plant Communities Suitable for Green Roofs in Arid Regions. Sustainability, 10, 1755. doi:10.3390/su10061755

124.Gomes MG, Silva CM, Valadas AS, Silva M (2019) Impact of Vegetation, Substrate, and Irrigation on the Energy Performance of Green Roofs in a Mediterranean Climate. Water 11, 2016. doi:10.3390/w11102016

125.Gong Y, Yin D, Fang X, Zhai D, Li J (2018a) Rainwater retention effect of extensive green roofs monitored under natural rainfall events – a case study in Beijing. Hydrology Research | in press. DOI:10.2166/nh.2018.144

126.Gong Y, Yin D, Fang X, Li J (2018b) Factors Affecting Runoff Retention Performance of Extensive Green Roofs. Water, 10, 1217; doi:10.3390/w10091217

127.Gong Y, Yin D, Li J, Zhang X, Wang W, Fang X, Shi H, Wang Q (2019) Performance assessment of extensive green roof runoff flow and quality control capacity based on pilot experiments. Science of the Total Environment 687, 505–515. https://doi.org/10.1016/j.scitotenv.2019.06.100

128.Gong Y, Zhang X, Li H, Zhang X, He S, Miao Y (2021) A comparison of the growth status, rainfall retention and purification effects of four green roof plant species. Journal of Environmental Management 278, 111451. https://doi.org/10.1016/j.jenvman.2020.111451

129.Griffin WN, Cohan SM, Lea-Cox JD, Ristvey AG (2017) Green Roof Substrate Composition Affects Phedimus kamtschaticus Growth and Substrate Water Content under Controlled Environmental Conditions. Hortscience 52(2):320–325. DOI:10.21273/HORTSCI11202-16

130.Grullon – Penkova IF, Zimmerman JK, Gonzalez G (2020) Green roofs in the tropics: design considerations and vegetation dynamics. Heliyon 6 (2020) e04712. DOI: 10.1016/j.heliyon.2020.e04712

131.Guo J, Zhang Y, Che S (2017) Performance analysis and experimental study on rainfall water purification with an extensive green roof matrix layer in Shanghai, China. Water Science & Technology | in press. DOI:10.2166/wst.2017.582

132.Guo W, Xu Y, Wu W, Ye Y, Chen P (2019) The complete chloroplast genome sequence of Belosynapsis ciliata (Blume) R. S. Rao (Commelinaceae). Mitochondrial Dna Part B, Vol. 4, No. 2, 2373–2374. doi: 10.1080/23802359.2019.1567288

133.Guo B, Arndt S, Miller R, Lu N, Farrell C (2021) Are succulence or trait combinations related to plant survival on hot and dry green roofs? Urban Forestry & Urban Greening 64, 127248. https://doi.org/10.1016/j.ufug.2021.127248

134.Halim MA, Vantellingen J, Gorgolewski AS, Rose WK, Drake JA, Margolis L, Thomas SC (2022) Greenhouse gases and green roofs: carbon dioxide and methane fluxes in relation to substrate characteristics. Urban Ecosystems (2022) 25:487–498. https://doi.org/10.1007/s11252-021-01166-8

135.He Y, Lin ES, Tan CL, Tan PY, Wong NH (2021a) Quantitative evaluation of plant evapotranspiration effect for green roof in tropical area: A case study in Singapore. Energy & Buildings 241, 110973. https://doi.org/10.1016/j.enbuild.2021.110973

136.He Y, Lin ES, Yu Z, Tan CL, Tan PY, Wong NH (2021b) The effect of dynamic albedos of plant canopy on thermal performance of rooftop greenery: A case study in Singapore. Building and Environment 205, 108247. https://doi.org/10.1016/j.buildenv.2021.108247

137.Heim A & Lundholm J (2014a) The effects of substrate depth heterogeneity on plant species coexistence on an extensive green roof. Ecological Engineering 68, 184–188. DOI:10.1016/j.ecoleng.2014.03.023

138.Heim A & Lundholm J (2014b) Species interactions in green roof vegetation suggest complementary planting mixtures. Landscape and Urban Planning 130, 125–133. DOI:10.1016/j.landurbplan.2014.07.007

139.Heim A, Lundholm J, Philip L (2014) The impact of mosses on the growth of neighbouring vascular plants, substrate temperature and evapotranspiration on an extensive green roof. Urban Ecosyst. DOI 10.1007/s11252-014-0367-y

140.Heim A & Lundholm J (2016) Phenological complementarity in plant growth and reproduction in a green roof ecosystem. Ecological Engineering 94, 82–87. DOI:10.1016/j.ecoleng.2016.05.018

141.Heim A & Lundholm J (2022) Changes in plant community composition and functional plant traits over a four-year period on an extensive green roof. Journal of Environmental Management 304, 114154. https://doi.org/10.1016/j.jenvman.2021.114154

142.Hoch JM, Rhodes ME, Shek K, Dinwiddie D, Hiebert TC, Gill A, Estrada AES, Griffin KL, Palmer MI, McGuire KL (2019) Soil microbial assemblages are linked to plant community composition and contribute to ecosystem services on urban green roofs. Frontier in Ecology and Evolution 7:198. doi:10.3389/fevo.2019.00198

143.Hooks T & Niu G (2019) Relative Salt Tolerance of Four Herbaceous Perennial Ornamentals. Horticulturae 5, 36; doi:10.3390/horticulturae5020036

144.Hu S, Liu L, Cao J, Chen N, Wang Z (2019) Water Resilience by Centipedegrass Green Roof: A Case Study. Buildings, 9, 141; doi:10.3390/buildings9060141

145.Huang YY, Chen CT, Tsai YC (2016) Reduction of temperatures and temperature fluctuations by hydroponic green roofs in a subtropical urban climate. Energy and Buildings http://dx.doi.org/10.1016/j.enbuild.2016.07.023

146.Huang YY, Chen CT, Liu WT (2018) Thermal performance of extensive green roofs in a subtropical metropolitan área. Energy and Buildings https://doi.org/10.1016/j.enbuild.2017.10.039

147.Huang YY, Ma TJ, Wang YS, Wang CK (2020) The application of non-Crassulacean acid metabolism edible plant and lightweight expanded clay aggregate to achieve joint benefits of thermal insulation mitigation and passive cooling strengthening

of extensive green roofs in subtropical regions. Solar Energy 201 (2020) 944–964. DOI:10.1016/j.solener.2020.03.029

148.Irfandi AM &Muslimsyah KH (2021) The Effect of Plants on Extensive Green Roofs in Urban Heat Island Mitigation Efforts in Humid Tropical Cities. The 5th International Conference on Rebuilding Place IOP Conf. Series: Earth and Environmental Science 881, 012043 IOP Publishing doi:10.1088/1755-1315/881/1/012043

149.Jacobs J, Berg M, Beenaerts N, Artois T (2022) Biodiversity of collembola on green roofs: A case study of three cities in Belgium. Ecological Engineering 177, 106572. https://doi.org/10.1016/j.ecoleng.2022.106572

150.Jacobs J, Beenaerts N, Artois T (2023) Green roofs and pollinators, useful green spots for some wild bee species (Hymenoptera: Anthophila), but not so much for hoverfies (Diptera: Syrphidae). Scientifc Reports | 13:1449 | https://doi.org/10.1038/s41598-023-28698-7

151.Jauni M, Kuoppamäki K, Hagner M, Prass M, Suonio T, Franssone A, Lehvävirt S (2020) Alkaline habitat for vegetated roofs? Ecosystem dynamics in a vegetated roof with crushed concrete-based substrate. Ecological Engineering 157 (2020) 105970. DOI:10.1016/j.ecoleng.2020.105970

152.Ji P, Sæbø A, Stovin V, Hanslin HM (2018) Sedum root foraging in layered green roof substrates. Plant Soil, 430:263–276. https://doi.org/10.1007/s11104-018-3729-z

153.Jim CY (2012) Effect of vegetation biomass structure on thermal performance of tropical green roof. Landscape Ecol Eng, 8:173–187. DOI 10.1007/s11355-011-0161-4

154.Jim CY (2014a) Air-conditioning energy consumption due to green roofs with different building thermal insulation. Applied Energy 128, 49–59. DOI: 10.1016/j.apenergy.2014.04.055

155.Jim CY (2014b) Heat-sink effect and indoor warming imposed by tropical extensive green roof. Ecological Engineering 62, 1– 12. DOI:10.1016/j.ecoleng.2013.10.022

156.Jim CY (2015) Assessing climate-adaptation effect of extensive tropical green roofs in cities. Landscape and Urban Planning 138, 54–70. http://dx.doi.org/10.1016/j.landurbplan.2015.02.014

157.John J, Lundholm J, Kernaghan G (2014) Colonization of green roof plants by mycorrhizal and rootendophytic fungi. Ecological Engineering 71, 651–659. DOI:10.1016/j.ecoleng.2014.08.012

158.Johnson C, Schweinhart S, Buffam I (2016) Plant species richness enhances nitrogen retention in green roof plots. Ecological Applications 26(7) DOI:10.1890/15-1850.1

159.Joimel S, Grard B, Chenu C, Cheval P, Mondy S, Lelievre M, Auclerc A, Gonod LV (2022) One green roof type, one Technosol, one ecological community. Ecological Engeneering 175, 106475. https://doi.org/10.1016/j.ecoleng.2021.106475

160.Ju JH, Xu H, Yeum KJ, Yoon YH (2020) Effects Of Hydrophilic Polymer On The Survival, Gr O W Th, And Flowering Characteristics Of Pineapple Sage Salvia Elegans In Unirrigated Green Roofs. Applied Ecology And Environmental Research 18(3):3887-3896. DOI: http://dx.doi.org/10.15666/aeer/1803_38873896

161.Ju JH, Cho SY, Song HY, Ju S, Yoon YH, Yeum KJ (2021a) Growth and Carotenoid Contents of Intercropped Vegetables in Building-Integrated Urban

Agriculture. Journal of Food Quality Volume 2021, Article ID 1159567, 9 pages https://doi.org/10.1155/2021/1159567

162.Ju JH, Yoon YH, Ju SY (2021b) Influence of Substrates and Hydrogels on Spearmint (Mentha spicata) Growth and Flowering in a Rooftop Garden. Hortscience 56(6):629–633. https://doi.org/10.21273/HORTSCI15540-20

163.Jusselme MD, Pruvost C, Motard E, Giusti-Miller S, Frechault S, Alphonse V, Balland-Bolou-Bi C, Dajoz I, Mora P (2019) Increasing the ability of a green roof to provide ecosystem services by adding organic matter and earthworms. Applied Soil Ecology 143, 61–69. DOI: 10.1016/j.apsoil.2019.05.028 . hal-02166091

164.Kanechi M, Fujiwara S, Shintani N, Suzuki T, Uno Y (2014) Performance of herbaceous Evolvulus pilosus on urban green roof in relation to substrate and irrigation. Urban Forestry & Urban Greening 13, 184–191. DOI:10.1016/j.ufug.2013.08.003

165. Kargas G, Ntoulas N, Tsapatsouli A (2023) Use of a Dielectric Sensor for Salinity Determination on an Extensive Green Roof Substrate. Sensors, 23, 5802. https://doi.org/10.3390/s23135802

166.Karimian Z, Hozhabralsadat MS, Heidari A (2023) Evaluating the ability of green roof plants in capturing air pollutants using biogas-digestate: Exploring physiological, biochemical, and anatomical characteristics. Environmental Pollution 333, 122071. https://doi.org/10.1016/j.envpol.2023.122071

167.Karyono TH, Vale R, Vale B (2017) Sustainable Building and Built Environments to Mitigate Climate Change in the Tropics Conceptual and Practical Approaches. Springer International Publishing AG. DOI 10.1007/978-3-319-49601-6

168.Kemp S, Hadley P, Blanuša T (2019) The influence of plant type on green roof rainfall retention. Urban Ecosystems, 22:355–366 https://doi.org/10.1007/s11252-018-0822-2

169.Klein PM & Coffman R (2015) Establishment and performance of an experimental green roof under extreme climatic conditions. Science of the Total Environment 512–513, 82–93. DOI: 10.1016/j.scitotenv.2015.01.020

170.Kohler M & Kaiser D (2021) Green Roof Enhancement on Buildings of the University of Applied Sciences in Neubrandenburg (Germany) in Times of Climate Change. Atmosphere, 12, 382. https://doi.org/10.3390/atmos12030382

171.Kohler M & Ksiazek-Mikenas K (2018) Green Roofs as Habitats for Biodiversity. Nature Based Strategies for Urban and Building Sustainability. DOI: https://doi.org/10.1016/B978-0-12-812150-4.00022-7

172.Kokkinou I, Ntoulas N, Nektarios PA, Varela D (2016) Response of Native Aromatic and Medicinal Plant Species to Water Stress on Adaptive Green Roof Systems. Hortscience 51(5):608–614. https://doi.org/10.21273/HORTSCI.51.5.608

173.Kolk HJ, Berg P, Korthals G, Bezemer TM (2020) Shading enhances plant species richness and diversity on an extensive green roof. Urban Ecosystems (2020) 23:935–943. DOI:10.1007/s11252-020-00980-w

174.Konopka J, Heusinger J, Weber S (2021) Extensive Urban Green Roof Shows Consistent Annual Net Uptake of Carbon as Documented by 5 Years of Eddy-Covariance Flux Measurem. Journal of Geophysical Research: Biogeosciences. DOI: 10.1029/2020JG005879

175.Koźmińska A, Al Hassan M, Wiszniewska A, Hanus-Fajerska E, Boscaiu M, Vicente O (2019) Responses of succulents to drought: Comparative analysis of four Sedum (Crassulaceae) species. Scientia Horticulturae 243, 235–242. https://doi.org/10.1016/j.scienta.2018.08.028

176.Krawczyk A, Domagała-Świątkiewicz I, Lis-Krzyścin A (2017) The effect of substrate on growth and nutritional status of native xerothermic species grown in

extensive green roof technology. Ecological Engineering 108, 194–202. DOI:10.1016/j.ecoleng.2017.08.022

177.Krawczyk A, Swiatkiewicz ID, Krzyscin AL (2021) Time-Dependent Changes in the Physico-Chemical Parameters and Growth Responses of Sedum acre (L.) to Waste-Based Growing Substrates in Simulation Extensive Green Roof Experiment. Agronomy, 11, 298. https://doi.org/10.3390/agronomy11020298

178.Krzyzak J, Rusinowski S, ˙ Sitko K, Szada-Borzyszkowska A, Borgulat J, Stec R, Hanslin HM, Pogrzeba M (2023) The Effect of Combined Drought and Temperature Stress on the Physiological Status of Calcareous Grassland Species as Potential Candidates for Urban Green Infrastructure. Plants 12, 2003. https://doi.org/10.3390/ plants12102003

179.Ksiazek K, Fant J, Skogen K (2012) An assessment of pollen limitation on Chicago green roofs. Landscape and Urban Planning 107, 401– 408. DOI:10.1016/j.landurbplan.2012.07.008

180.Ksiazek-Mikenas K, Chaudhary VB, Larkin DJ, Skogen KA (2021) A habitat analog approach establishes native plant communities on green roofs. Exosphere, vol 12 (9), e03754. DOI:10.1002/ecs2.3754

181.Kuronuma T & Watanabe H (2016) Physiological and Morphological Traits and Competence for Carbon Sequestration of Several Green Roof Plants under a Controlled Environmental System. J. AMER. SOC. HORT. SCI. 141(6):583–590. https://doi.org/10.21273/JASHS03909-16

182.Kuronuma T & Watanabe H (2017) Photosynthetic and Transpiration Rates of Three Sedum Species Used for Green Roofs. Environ. Control Biol., 55 (3), 137-141. DOI:10.2525/ecb.55.137

183.Kuronuma T, Watanabe H, Ishihara T, Kou D, Toushima K, Ando M, Shindo S (2018) CO_2 Payoff of Extensive Green Roofs with Different Vegetation Species. Sustainability, 10, 2256; doi:10.3390/su10072256.

184.Lati CA, Nurdiani N, Katarina W (2021) The study of green roof application in apartments in Indonesia. IOP Conf. Series: Earth and Environmental Science 794 012165 IOP Publishing doi:10.1088/1755-1315/794/1/012165

185.Lee S, Ryu Y, Jiang C (2015) Urban heat mitigation by roof surface materials during the East Asian summer monsoon. Environ. Res. Lett. 10 (2015) 124012 doi:10.1088/1748-9326/10/12/124012

186.Leso L, Morshed W, Conti L, Barbari M (2017) Evaluating thermal performance of experimental building solutions designed for livestock housing: the effect of greenery systems. Agronomy Research 15(1), 239–248. https://www.cabidigitallibrary.org/doi/pdf/10.5555/20173199338

187.Li J, Wai OWH, Li YS, Zhan J, Ho YA, Li J, Lam E (2010) Effect of green roof on ambient CO_2 concentration. Building and Environment 45, 2644-2651. DOI: 10.1016/j.buildenv.2010.05.025

188.Li JH, Hsieh JC, Lou CW, Hsieh CT, Pan YJ, Hsing WH, Lin JH (2016) Using Nonwoven Fabrics as Culture Mediums for Extensive Green Roofs: Physical Properties and Cooling Effect. Fibers and Polymers, Vol.17, No.7, 1111-1114. DOI:10.1007/s12221-016-6445-4

189.Li X, Cao J, Xu PX, Fei L, Dong Q, Wang ZL (2018) Green Roofs: Effects of Plant Species Used On Runoff. Land Degradation & Development volume 29, issue 10, 3628-3638. DOI:10.1002/ldr.3102

190.Liao W, Drake J, Thomas SC (2022) Biochar granulation enhances plant performance on a green roof substrate. Science of the Total Environment 813, 152638. http://dx.doi.org/10.1016/j.scitotenv.2021.152638

191.Liberalesso T, Tassi R, Ceconi DE, Allasia DG (2021) Effect of rice husk addition on the physicochemical and hydrological properties on green roof substrates under subtropical climate conditions. Journal of Cleaner Production 315, 128133. https://doi.org/10.1016/j.jclepro.2021.128133

192.Licht J & Smith NG (2021) Pyrogenic carbon improves the physiological performance of a C3 species planted on a green roof. Israel Journal of Ecology & Evolution. http://dx.doi.org/10.1163/22244662-bja10004

193.Lin YJ & Lin HT (2011) Thermal performance of different planting substrates and irrigation frequencies in extensive tropical rooftop greeneries. Building and Environment 46, 345-355. DOI:10.1016/j.buildenv.2010.07.027

194.Lin YJ, Su AT, Lin BS (2017) Cooling Performances on Rainless Days of Extensive Green Roofs Planted with Different Ornamental Species. Hortscience 52(3):467–474. doi: 10.21273/HORTSCI11507-16

195.Liu J, Shrestha P, Skabelund LR, Todd T, Decker A, Kirkhamd MB (2019c) Growth of prairie plants and sedums in different substrates on an experimental green roof in Mid-Continental USA. Science of the Total Environment 697, 134089. https://doi.org/10.1016/j.scitotenv.2019.134089

196.Liu L, Cao J, Ali M, Zhang J, Wang Z (2021) Impact of green roof plant species on domestic wastewater treatment. Environmental Advances 4, 100059. http://doi.org/10.1016/j.envadv.2021.100059

197.Liu R, Stanford RL, Deng Y, Liu D, Liu Y, Yu SL (2019a) The influence of extensive green roofs on rainwater runoff quality: a field-scale study in southwest

China. Environmental Science and Pollution Research https://doi.org/10.1007/s11356-019-06151-5

198.Liu TC, Shyu GC, Fang WT, Liu SY, Cheng BY (2012) Drought tolerance and thermal effect measurements for plants suitable for extensive green roof planting in humid subtropical climates. Energy and Buildings 47, 180–188. DOI:10.1016/j.enbuild.2011.11.043

199.Liu W, Wei W, Chen W, Deo RC, Si J, Xi H, Li B, Feng Q (2019b) The impacts of substrate and vegetation on stormwater runoff quality from extensive green roofs. Journal of Hydrology 576, 575–582. DOI:10.1016/j.jhydrol.2019.06.061

200.Liu W, Feng Q, Chen W, Wei W, Deo RC (2019d) The influence of structural factors on stormwater runoff retention of extensive green roofs: new evidence from scale-based models and real experiments. Journal of Hydrology 569, 230-238. https://doi.org/10.1016/j.jhydrol.2018.11.066

201.Liu W, Feng Q, Chen W, Wei W, Si J, Xi H (2019e) Runoff retention assessment for extensive green roofs and prioritization of structural factors at runoff plot scale using the Taguchi method. Ecological Engineering 138, 281-288. https://doi.org/10.1016/j.ecoleng.2019.07.033

202.Liu H, Yin H, Kong F, Middel A, Zheng X, Huang J, Sun T, Wang D, Lensky IM (2022) Change of nutrients, microorganisms, and physical properties of exposed extensive green roof substrate. Science of the Total Environment, 805, 150344. https://doi.org/10.1016/j.scitotenv.2021.150344

203.Llorach-Massana P, Cirrincione L, Sierra-Perez J, Scaccianoce G, Gennusa ML, Peña J, Rieradevall J (2023) Environmental assessment of a new building envelope material derived from urban agriculture wastes: the case of the tomato plants

stems. The International Journal of Life Cycle Assessment, 28:813–827 https://doi.org/10.1007/s11367-023-02152-2

204.Lonnqvist J, Blecken GT, Viklander M (2021a) Vegetation cover and plant diversity on cold climate green roofs. Journal of Urban Ecology, 1-3. doi: 10.1093/jue/juaa035

205.Lonnqvist J, Hanslin HM, Johannessen BG, Muthanna TM, Viklander M, Blecken G (2021b) Temperatures and precipitation affect vegetation dynamics on Scandinavian extensive green roofs. International Journal of Biometeorology 65:837–849. https://doi.org/10.1007/s00484-020-02060-2

206.Lönnqvist J, Farrell C, Schrieke D, Viklander M, Blecken GT (2023) Plant water use related to leaf traits and CSR strategies of 10 common European green roof species. Science of the Total Environment 890, 164044. http://dx.doi.org/10.1016/j.scitotenv.2023.164044

207.Lu J, Yuan J, Yang J, Yang Z (2014) Responses of morphology and drought tolerance of *Sedum lineare* to watering regime in green roof system: A root perspective. Urban Forestry & Urban Greening 13: 682–688. DOI:10.1016/j.ufug.2014.08.003

208.Lu J, Yuan J, Yang J, Chen A, Yang Z (2015) Effect of substrate depth on initial growth and drought tolerance of *Sedum lineare* in extensive green roof system. Ecological Engineering 74, 408–414. DOI:10.1016/j.ecoleng.2014.11.018

209.Lundholm JT (2015) Green roof plant species diversity improves ecosystem multifunctionality. Journal of Applied Ecology, 52, 726–734. DOI:10.1111/1365-2664.12425

210.Lundholm J, MacIvor JS, MacDougall Z, Ranalli M (2010) Plant Species and Functional Group Combinations Affect Green Roof Ecosystem Functions. PlosONE, vol 5, issue 3. https://doi.org/10.1371/journal.pone.0009677

211.Lundholm J, Heim A, Tran S, Smith T (2014) Leaf and Life History Traits Predict Plant Growth in a Green Roof Ecosystem. PlosONE, vol 9, issue 6. https://doi.org/10.1371/journal.pone.0101395

212.Luo H, Liu X, Anderson BC, Zhang K, Li X, Huang B, Li M, Mo Y, Fan L, Shen Q, Chen F, Jiang M (2015) Carbon sequestration potential of green roofs using mixed-sewage- sludge substrate in Chengdu World Modern Garden City. Ecological Indicators 49, 247–259. DOI 10.1016/j.ecolind.2014.10.016

213.Maceda-Veiga A, MacNally R, Rodriguez S, Szabo S, Peeters ETHM, Ruff T, Salvado H (2022) Effects of two submerged macrophyte species on microbes and metazoans in rooftop water-storage ponds with different labile carbon loadings. Water Research 211, 117999. https://doi.org/10.1016/j.watres.2021.117999

214.MacIvor JS & Lundholm J (2011) Performance evaluation of native plants suited to extensive green roof conditions in a maritime climate. Ecological Engineering 37 (2011) 407–417. https://doi.org/10.1016/j.ecoleng.2010.10.004

215.MacIvor JS, Ranalli MA, Lundholm JT (2011) Performance of dryland and wetland plant species on extensive green roofs. Annals of Botany 107: 671–679, doi:10.1093/aob/mcr007

216.MacIvor JS, Margolis L, Puncher CL, Matthews BJC (2013) Decoupling factors affecting plant diversity and cover on extensive green roofs. Journal of Environmental Management 130, 297-305. DOI: 10.1016/j.jenvman.2013.09.014

217.MacIvor JS, Sookhan N, Arnillas CA, Bhatt A, Das S, Yasui SLE, Xie G, Cadotte MW (2018) Manipulating plant phylogenetic diversity for green roof ecosystem

service delivery. Evolutionary Applications, 11: 2014–2024. https://doi.org/10.1111/eva.12703

218.Madre F, Vergnes A, Machon N, Clergeau P (2013) A comparison of 3 types of green roof as habitats for arthropods. Ecological Engineering 57, 109– 117. DOI:10.1016/j.ecoleng.2013.04.029

219.Madre F, Vergnes A, Machon N, Clergeau P (2014) Green roofs as habitats for wild plant species in urban landscapes: First insights from a large-scale sampling. Landscape and Urban Planning 122, 100– 107. DOI:10.1016/j.landurbplan.2013.11.012

220.Mandel L, McCoy E, Liss T (2016) Reference Community: Adapting Native Plants to North American Green Roofs. Journal of Green Building, vol 11, num 4. DOI:10.3992/jgb.11.4.15.1

221.Manso M, Castro-Gomes JP, Marchacz M, Górski M, Dulak L, Zuchowski R (2017) Acoustic Evaluation Of A New Modular System For Green Roofs And Green Walls. Architecture Civil Engineering Environment 2. DOI:10.21307/acee-2017-023

222.Marouli C, Savvidou P, Koutsokali M, Papadopoulou P, Misseyanni A, Tsiliki G and Georgas D (2022) Plant growth on a Mediterranean green roof: a pilot study on influence of substrate depth, substrate composition, and type of green roof. Front. Sustain. Cities 3:796441. doi: 10.3389/frsc.2021.796441

223. Martini AN, Papafotioua M, Evangelopoulos K (2017) Effect of substrate type and depth on the establishment of the edible and medicinal native species Crithmum maritimum on an extensive urban Mediterranean green roof. Acta Hortic. 1189. DOI 10.17660/ActaHortic.2017.1189.89

224.Martini AN, Papafotiou M, Massas I, Chorianopoulou N (2022) Using the Halophyte Crithmum maritimum in Green Roofs for Sustainable Urban Horticulture:

Effect of Substrate and Nutrient Content Analysis including Potentially Toxic Elements. Sustainability, 14, 4713. https://doi.org/10.3390/su14084713

225.Martini AN, Papafotiou M, Massas I, Chorianopoulou N (2023) Growing of the Cretan Therapeutic Herb Origanum Dictamnus in The Urban Fabric: The Effect of Substrate and Cultivation Site on Plant Growth and Potential Toxic Element Accumulation. Plants, 12, 336. https://doi.org/10.3390/ plants12020336

226.Matlock JM & Rowe DB (2017) Does Compost Selection Impact Green Roof Substrate Performance? Measuring Physical Properties, Plant Development, and Runoff Water Quality. Compost Science & Utilization Vol. 0, No. 0, 1–11 https://doi.org/10.1080/1065657X.2017.1295887

227.Matsuoka T, Tsuchiya K, Yamada S, Lundholm J, Okuro T (2019) Value of Sedum species as companion plants for nectar-producing plants depends on leaf characteristics of the Sedum. Urban Forestry & Urban Greening 39, 35–44. DOI:10.1016/j.ufug.2019.02.003

228.Matsuoka T, Tsuchiya K, Yamada S, Lundholm J, Okuro T (2020) The effects of growth form on the impact of companion planting of nectar-producing plant species with Sedum album for extensive green roofs. Urban Forestry & Urban Greening 56 (2020) 126875. DOI:10.1016/j.ufug.2020.126875

229.Mazzeo D, Matera N, Peri G, Scaccianoce G (2023) Forecasting green roofs’ potential in improving building thermal performance and mitigating urban heat island in the Mediterranean area: An artificial intelligence-based approach. Applied Thermal Engineering 222, 119879. https://doi.org/10.1016/j.applthermaleng.2022.119879

230.Meetam M, Sripintusorn N, Songnuan W, Siriwattanakul U, Pichakum A (2020) Assessment of physiological parameters to determine drought tolerance of plants

for extensive green roof architecture in tropical areas, Urban Forestry and amp; Urban Greening, doi: https://doi.org/10.1016/j.ufug.2020.126874

231.Meng R, Zhang Q, Li D, Wang H (2021) Influence of Substrate Layer Thickness and Biochar on the Green Roof Capacity to Intercept Rainfall and Reduce Pollution in Runoff. Pol. J. Environ. Stud. Vol. 30, No. 5, 4085-4103. DOI, 10.15244/pjoes/132810

232.Minixhofer, P.; Scharf, B.; Hafner, S.; Weiss, O.; Henöckl, C.; Greiner, M.; Room, T.; Stangl, R. Towards the Circular Soil Concept: Optimization of Engineered Soils for Green Infrastructure Application. Sustainability 2022, 14, 905. https:// doi.org/10.3390/su14020905

233.Mitchell ME, Emilsson T, Buffam I (2021) Carbon, nitrogen, and phosphorus variation along a green roof chronosequence: Implications for green roof ecosystem development. Ecological Engineering 164, 106211. https://doi.org/10.1016/j.ecoleng.2021.106211

234.Molineux CJ, Gange AC, Newport DJ (2016) Using soil microbial inoculations to enhance substrate performance on extensive green roofs. Science of the Total Environment 550: 846–856. DOI: 10.1016/j.scitotenv.2016.12.031

235.Momose T & Lundholm J (2021) Use of a thermo-module as a soil heat flux sensor: Applications in the evaluation of extensive green roof thermal performance. Energy & Buildings, Article in press. https://doi.org/10.1016/j.enbuild.2020.110562

236.Monteiro CM, Calheiros CSC, Pimentel-Rodrigues C, Silva-Afonso A, Castro PML (2016) Contributions to the design of rainwater harvesting systems in buildings with green roofs in a Mediterranean climate. Water Science & Technology, 73.8. DOI:10.2166/wst.2016.034

237.Monteiro CM, Calheiros CSC, Palha P, Castro PML (2017a) Growing substrates for aromatic plant species in green roofs and water runoff quality: pilot experiments in a Mediterranean climate. Water Science & Technology | in press. DOI: 10.2166/wst.2017.276

238.Monteiro CM, Calheiros CSC, Martins JP, Costa FM, Palha P, Freitas S, Ramos NMM, Castro PML (2017b) Substrate influence on aromatic plant growth in extensive green roofs in a Mediterranean climate. Urban Ecosyst. DOI 10.1007/s11252-017-0687-9.

239.Moritani S, Yamamoto T, Andry H, Inoue M, Kato K, Saito H (2013) Effect of combined water and salinity stress factors on evapotranspiration of Sedum kamtschaticum Fischer in relation to green roof irrigation. Urban Forestry & Urban Greening 12 (2013) 338–343. DOI:10.1016/j.ufug.2013.04.005

240.Moritani S, Yamamoto T, Andry H, Saito H (2017) Evapotranspiration and Mineral Content of Sedum kamtschaticum Fischer Under Saline Irrigation. Communications In Soil Science And Plant Analysis https://doi.org/10.1080/00103624.2017.1358743

241.Nagase A (2020) Novel application and reused materials for extensive green roof substrates and drainage layers in Japan – Plant growth and moisture uptake implementation. Ecological Engineering 153, 105898. DOI:10.1016/j.ecoleng.2020.105898

242.Nagase A & Dunnett N (2010) Drought tolerance in different vegetation types for extensive green roofs: Effects of watering and diversity. Landscape and Urban Planning 97, 318–327. DOI:10.1016/j.landurbplan.2010.07.005

243.Nagase A & Dunnett N (2011) The relationship between percentage of organic matter in substrate and plant growth in extensive green roofs. Landscape and Urban Planning 103, 230– 236. DOI:10.1016/j.landurbplan.2011.07.012

244.Nagase A & Dunnett N (2012) Amount of water runoff from different vegetation types on extensive green roofs: Effects of plant species, diversity and plant structure. Landscape and Urban Planning 104, 356– 363. https://doi.org/10.1016/j.landurbplan.2011.11.001

245.Nagase A & Dunnett N (2013a) Performance of geophytes on extensive green roofs in the United Kingdom. Urban Forestry & Urban Greening 12 509–521. DOI:10.1016/j.ufug.2013.06.005

246.Nagase A & Dunnett N (2013b) Establishment of an annual meadow on extensive green roofs in the UK. Landscape and Urban Planning 112, 50– 62. DOI:10.1016/j.landurbplan.2012.12.007

247.Nagase A, Dunnett N, Choi M (2013) Investigation of weed phenology in an establishing semi-extensive green roof. Ecological Engineering 58, 156– 164. DOI:10.1016/j.ecoleng.2013.06.007

248.Nagase A, Dunnett N, Choi M (2017) Investigation of plant growth and flower performance on a semi-extensive green roof. Urban Forestry and Urban Greening http://dx.doi.org/10.1016/j.ufug.2017.01.013

249.Nagase A & Tashiro-Ishii Y (2018) Habitat template approach for green roofs using a native rocky sea coast plant community in Japan. Journal of Environmental Management 206, 255-265. DOI: 10.1016/j.jenvman.2017.10.001

250.Nardini A, Andri S, Crasso M (2012) Influence of substrate depth and vegetation type on temperature and water runoff mitigation by extensive green roofs:

shrubs versus herbaceous plants. Urban Ecosyst, 15:697–708 DOI 10.1007/s11252-011-0220-5

251.Nascimento CML, Mary W, Silva LP (2018) Hydrological performance of modular-tray green roof systems for increasing the resilience of mega-cities to climate change. Journal of Hydrology, doi: https://doi.org/10.1016/j.jhydrol.2018.01.004

252.Nektarios PA, Amountzias I, Kokkinou I, Ntoulas N (2011) Green Roof Substrate Type and Depth Affect the Growth of the Native Species Dianthus fruticosus Under Reduced Irrigation Regimens. Hortscience 46(8):1208–1216. DOI:10.21273/HORTSCI.46.8.1208

253.Nektarios PA, Kokkinou I, Ntoulas N (2020) The effects of substrate depth and irrigation regime, on seeded Sedum species grown on urban extensive green roof systems under semi-arid Mediterranean climatic conditions. Journal of Environmental Management 279: 111607. DOI: 10.1016/j.jenvman.2020.111607

254.Nektarios PA, Ntoulas N, Nydrioti E, Kokkinou I, Bali EM, Amountzias I (2015) Drought stress response of Sedum sediforme grown in extensive greenroof systems with different substrate types and depths. Scientia Horticulturae 181, 52–61. DOI:10.1016/j.scienta.2014.10.047

255.Nektarios PA, Nydrioti E, Kapsali T, Ntoulas N (2016a) Crithmum maritimum growth in extensive green roof systems with different substrate type, depth and irrigation regime. Acta Hortic. 1108. DOI 10.17660/ActaHortic.2016.1108.40

256.Nektarios PA, Nydrioti E, Kapsali T, Ntoulas N (2016b) Substrate type, depth and irrigation regime effects on *Ebenus cretica* growth in extensive green roof. Acta Hortic. 1108. DOI 10.17660/ActaHortic.2016.1108.39

257.Noya MG, Cuquel FL, Schafer G, Armindo RA (2017) Substrates for cultivating herbaceous perennial plants in extensive green roofs. Ecological Engineering 102, 662–669. DOI:10.1016/j.ecoleng.2017.02.042

258.Ntoulas N & Nektarios PA (2015) Paspalum vaginatum drought tolerance and recovery in adaptive extensive green roof systems. Ecological Engineering 82, 189–200. DOI:10.1016/j.ecoleng.2015.04.091

259.Ntoulas N, Nektarios PA, Nydrioti E (2013a) Performance of Zoysia matrella 'Zeon' in Shallow Green Roof Substrates under Moisture Deficit Conditions. Hortscience 48(7):929–937. DOI:10.21273/HORTSCI.48.7.929

260.Ntoulas N, Nektarios PA, Charalambous E, Psaroulis A (2013b) Zoysia matrella cover rate and drought tolerance in adaptive extensive green roof systems. Urban Forestry & Urban Greening 12, 522–531. DOI:10.1016/j.ufug.2013.07.006

261.Ntoulas N, Nektarios PA, Kotopoulis G, Ilia P, Ttooulou T (2017) Quality assessment of three warm-season turfgrasses growing in different substrate depths on shallow green roof systems. Urban Forestry & Urban Greening 26: 163-168. DOI:10.1016/j.ufug.2017.03.005

262.O'Carroll DM, Si ME, Staniec M, Voogt JA, Lundholm JT, Smart CC, Robinson CE (2023) Influence of vegetation type and climatological conditions on evapotranspiration from extensive green roofs. Journal of Hydrology 617, 128951. https://doi.org/10.1016/j.jhydrol.2022.128951

263.Oliveira CC (2021) Substratos para uso em telhados verdes: Avaliação da retenção hídrica e qualidade da água de escoamento. Dissertação. Programa de Pós-Graduação em Engenharia Ambiental da Universidade do Estado do Rio de Janeiro. http://www.bdtd.uerj.br/handle/1/10981

264.Olly LM, Bates AJ, Sadler JP, Mackay R (2011) An initial experimental assessment of the influence of substrate depth on floral assemblage for extensive green roofs. Urban Forestry & Urban Greening 10: 311– 316. DOI:10.1016/j.ufug.2011.07.005

265.Olszewski MW & Eisenman SW (2017) Influence of Biochar Amendment on Herb Growth in a Green Roof Substrate. Hortic. Environ. Biotechnol. 58(4):406-413. DOI:10.1007/978-3-031-34593-7_71

266.Olszewski MW, Holmes MH, Young CA (2010) Assessment of Physical Properties and Stonecrop Growth in Green Roof Substrates Amended with Compost and Hydrogel. HortTechnology 20 (2). DOI:10.21273/HORTTECH.20.2.438

267.Ondoño S, Martínez-Sánchez JJ, Moreno JL (2015) Evaluating the growth of several Mediterranean endemic species in artificial substrates: Are these species suitable for their future use in green roofs? Ecological Engineering 81: 405–417. DOI:10.1016/j.ecoleng.2015.04.079

268.Ondoño S, Martínez-Sánchez JJ, Moreno JL (2016a) The composition and depth of green roof substrates affect the growth of Silene vulgaris and Lagurus ovatus species and the C and N sequestration under two irrigation conditions. Journal of Environmental Management 166, 330-340. DOI: 10.1016/j.jenvman.2015.08.045

269.Ondoño S, Martínez-Sánchez JJ, Moreno JL (2016b) The inorganic component of green roof substrates impacts the growth of Mediterranean plant species as well as the C and N sequestration potential. Ecological Indicators 61, 739–752. DOI:10.1016/j.ecolind.2015.10.025

270.Ondoño S, Martínez-Sánchez JJ, Moreno JL (2018) Carbon and Nitrogen Sequestration Potential of Mediterranean Green Roofs Prototypes. Soil Management and Climate Change. https://doi.org/10.1016/B978-0-12-812128-3.00007-0

271. Ouldboukhitine SE, Spolek G, Belarbi R (2014) Impact of plants transpiration, grey and clean water irrigation on thethermal resistance of green roofs. Ecological Engineering 67, 60–66. DOI:10.1016/j.ecoleng.2014.03.052

272. Oviedo N & Torres A (2014) Attenuation and the hydrologic benefits of implementing eco-productive green roofs in marginal urban area. Ingeniería y Universidad. vol. 18 (2): 291-308. http://dx.doi.org/10.11144/Javeriana.IYU18-2.hahb

273. Oviedo N, Torres A, Devia C, Puentes A (2016) Hydric Attenuation of a Green Roof Experimental Assembly in Bogotá. Ambiente y Desarrollo, Bogotá (Colombia) Vol. XX (38) 53-68. DOI:10.11144/Javeriana.ayd20-38.hagr

274. Papafotiou M, Pergialioti N, Tassoula L, Massas I, Kargas G (2013a) Growth of native aromatic xerophytes in an extensive Mediterranean green roof as affected by substrate type and depth and irrigation frequency. Hortscience 48(10):1327–1333. DOI:10.21273/HORTSCI.48.10.1327

275. Papafotiou M, Pergialioti N, Massas I, Kargas G (2013b) Effect of substrate type and depth and the irrigation frequency on growth of semiwoody Mediterranean species in green roofs. Proc. IInd IS on Woody Ornamentals of the Moderate Zone. Acta. Hort. 990. https://doi.org/10.17660/ActaHortic.2013.990.62

276. Papafotiou M, Tassoula L, Liakopoulos G, Kargas G (2016) Effect of substrate type and irrigation frequency on growth of Mediterranean xerophytes on green roofs. Acta Hortic. 1108. DOI 10.17660/ActaHortic.2016.1108.41

277. Parada F, Gabarrell X, Rufi-Salis M, Arcas-Pilz V, Munoz P, Villalba G (2021) Optimizing irrigation in urban agriculture for tomato crops in rooftop greenhouses. Science of the Total Environment 794, 148689. https://doi.org/10.1016/j.scitotenv.2021.148689

278.Paraskevopoulou AT, Zafeiriou S, Londra PA (2021) Plant growth of Atriplex portulacoides affected by irrigation amount and substrate type in an extensive green roof system. Ecological Engineering 165, 106223. https://doi.org/10.1016/j.ecoleng.2021.106223

279.Paraskevopoulou AT, Ntoulas N, Bourtsoukli D, Bertsouklis K (2023) Effect of seawater irrigation on arthrocnemum macrostachyum growing in extensive green roof systems under semi-arid Mediterranean climatic conditions. Agronomy, 13, 1198. https:// doi.org/10.3390/agronomy13051198

280.Paraskevopoulou AT, Tsarouchas P, Londra PA, Kamoutsis AP (2020) The Effect of Irrigation Treatment on the Growth of Lavender Species in an Extensive Green Roof System. Water, 12, 863; doi:10.3390/w12030863

281.Pardela L, Kowalczyk T, Bogacz A, Kasowska D (2020) Sustainable Green Roof Ecosystems: 100 Years of Functioning on Fortifications—A Case Study. Sustainability, 12, 4721; doi:10.3390/su12114721

282.Peng LLH, Yang X, He Y, Hu Z, Xu T, Jiang Z, Yao L (2019) Thermal and energy performance of two distinct green roofs: Temporal pattern and underlying factors in a subtropical climate. Energy & Buildings 185, 247–258. DOI:10.1016/j.enbuild.2018.12.040

283.Pérez G, Vila A, Solé C, Coma J, Castell A, Cabeza LF (2015) The thermal behaviour of extensive green roofs under low plant coverage conditions. Energy Efficiency DOI 10.1007/s12053-015-9329-3

284.Pérez G, Chocarro C, Juárez A, Coma J (2020) Evaluation of the development of five Sedum species on extensive green roofs in a continental Mediterranean climate. Urban Forestry & Urban Greening 48: 126566. DOI:10.1016/j.ufug.2019.126566

285.Perillo VL, Brendel AS, Ferrelli F, Gutierrez A, Vitale AJ, Marinangeli P, Piccolo MC (2023) CO2 flux dynamics of exotic and native species in an extensive green roof simulator with hydric déficit. Urban Climate 49, 101567. https://doi.org/10.1016/j.uclim.2023.101567

286.Pessoa VG, Guiselini C, Montenegro AAA, Pandorfi H, Barbosa Filho JAD, Vicente TFS (2022) Carbon sequestration by plant species used in green roofs across different periods. Revista Brasileira de Engenharia Agrícola e Ambiental 26, 6, 407-411. DOI: http://dx.doi.org/10.1590/1807-1929/agriambi.v26n6p407-411

287.Petra SA, Georgescu MI, Manescu CR, Toma F, Badea ML, Dobrescu E, Popa VI (2020) Leaves anatomical and physiological adaptations of *Vinca major* 'Variegata' and *Hedera helix* L. to specific roof garden conditions. Notulae Botanicae Horti Agrobotanici Cluj-Napoca 47(4):318-328 DOI:10.15835/nbha48111784.

288.Petry C, Scalon FM, Ventura ME, Dall'Agnese L, Tedesco CD, Garcia NBU (2016) The propagation of Persicaria capitata in different substrates for green roof use. Acta Hortic. 1108. DOI 10.17660/ActaHortic.2016.1108.42

289.Pianella A, Aye L, Chen Z, Williams NSG (2020) Effects of substrate depth and native plants on green roof thermal performance in South-East Australia. Earth and Environmental Science 588, 022057. DOI:10.1088/1755-1315/588/2/022057

290.Pichakum N & Pichakum A (2021) Evaluating the Drought Endurance of Landscaping Ground Cover Plants in a Roof Top Model. Horticulturae, 7, 31. https://doi.org/10.3390/ horticulturae7020031

291.Piro P, Carbone M, De Simone M, Maiolo M, Bevilacqua P, Arcuri N (2018) Energy and hydraulic performance of a vegetated roof in sub-Mediterranean climate. Sustainability 10, 3473; doi:10.3390/su10103473

292.Pisello AL, Piselli C, Cotana F (2015) Thermal-physics and energy performance of an innovative green roof system: The Cool-Green Roof. Solar Energy 116, 337–356. DOI:10.1016/j.solener.2015.03.049

293.Price JG, Watts SA, Wright AN, Peters RW, Kirby JT (2011) Irrigation lowers substrate temperature and enhances survival of plants on green roofs in the southeastern United States. HortTechnology (21) 5. DOI:10.21273/HORTTECH.21.5.586

294.Procaccini G & Monticelli C (2021) A Green Roof Case Study in the Urban Context of Milan: Integrating the Residential and Cultivation Functions for Sustainable Development. Water, 13, 137. https://doi.org/10.3390/w13020137

295.Rabbani M & Kazemi F (2022) Water need and water use efficiency of two plant species in soil-containing and soilless substrates under green roof conditions. Journal of Environmental Management 302, 113950. https://doi.org/10.1016/j.jenvman.2021.113950

296.Radhakrishnan M, Kenzhegulova I, Eloffy MG, Ibrahim WA, Zevenbergen C, Pathirana A (2019) Development of context specific sustainability criteria for selection of plant species for green urban infrastructure: The case of Singapore. Sustainable Production and Consumption 20, 16–325. DOI:10.1016/j.spc.2019.08.004

297.Raimondo F, Trifilò P, Gullo MAL, Andri S, Savi T, Nardini A (2015) Plant performance on Mediterranean green roofs: interaction of species-specific hydraulic strategies and substrate water relations. Annals of Botany http://aobpla.oxfordjournals.org/ DOI:10.1093/aobpla/plv007

298.Rayner JP, Farrell C, Raynor KJ, Murphy SM, Williams NSG (2016) Plant establishment on a green roof under extreme hot and dryconditions: The importance of

leaf succulence in plant selection. Urban Forestry & Urban Greening 15, 6–14. DOI:10.1016/j.ufug.2015.11.004

299.Razzaghmanesh M, Beecham S, Kazemi F (2014a) The growth and survival of plants in urban green roofs in a dry climate. Science of the Total Environment 476–477, 288–297. DOI: 10.1016/j.scitotenv.2014.01.014

300.Razzaghmanesh M, Beecham S, Brien CJ (2014b) Developing resilient green roofs in a dry climate. Science of the Total Environment 490, 579–589. DOI:10.1016/j.scitotenv.2014.05.040

301.Rey CV, Franco N, Peyre G , Rodríguez JP (2020) Green Roof Design with Engineered Extensive Substrates and Native Species to Evaluate Stormwater Runoff and Plant Establishment in a Neotropical Mountain Climate. Sustainability 12, 6534; doi:10.3390/su12166534

302.Richardson ML & Arlotta CG (2021) Differential yield and nutrients of Hibiscus sabdariffa L. genotypes when grown in urban production systems. Scientia Horticulturae 288, 110349. https://doi.org/10.1016/j.scienta.2021.110349

303.Ridzuan NH, Farouk SA, Razak SA, Avicor SW, Taib N, Hamzah SN (2022) Insect biodiversity of urban green spaces in Penang Island, Malaysia. International Journal of Tropical Insect Science, 42:275–284. https://doi.org/10.1007/s42690-021-00543-2

304.Rivière L, Sellier A, Dutoit T, Vidaller C, Buisson E, Mahy G (2023) The contribution of seedbank to the green roof plant community dynamics analogous to semi-natural grasslands. Front. Ecol. Evol. 11:1152319. doi: 10.3389/fevo.2023.1152319

305.Robbiati FO, Cáceres N, Hick EC, Suarez M, Soto S, Barea G, Matoff E, Galetto L, Imhof L (2022) Vegetative and thermal performance of an extensive vegetated

roof located in the urban heat island of a semiarid region. Building and Environment 212, 108791. https://doi.org/10.1016/j.buildenv.2022.108791

306.Rocha B, Paço TA, Luz AC, Palha P, Milliken S, Kotzen B, Branquinho C, Pinho P, de Carvalho RC (2021) Are biocrusts and xerophytic vegetation a viable green roof typology in a Mediterranean climate? a comparison between differently vegetated green roofs in water runoff and water quality. Water 13, 94. https://doi.org/10.3390/w13010094

307.Rodriguez J & Vilela K (2022) Influence of the types of grass of green roofs for the design of thermal comfort in buildings. Journal of Ecological Engineering 23, 3, 223-229, https://doi.org/10.12911/22998993/146191

308.Romali NS, Ardzu FAB, Suzany MN (2023) The potential of coconut waste as green roof materials to improve stormwater runoff. Water Science & Technology Vol 87 No 6, 1515 doi: 10.2166/wst.2023.060

309.Rowe DB, Getter KL, Durhman AK (2012) Effect of green roof media depth on Crassulacean plant succession over seven years. Landscape and Urban Planning 104, 310– 319. DOI:10.1016/j.landurbplan.2011.11.010

310.Rowe DB, Kolp MR, Greer SE, Getter KL (2014) Comparison of irrigation efficiency and plant health of overhead, drip, and sub-irrigation for extensive green roofs. Ecological Engineering 64, 306–313. DOI:10.1016/j.ecoleng.2013.12.052

311.Rufí-Salís M, Petit-Boix A, Villalba G, Sanjuan-Delmas D, Parada F, Ercilla-Montserrat M, Arcas-Pilz V, Munoz-Liesa J, Rieradevall J, Gabarrell X (2020a) Recirculating water and nutrients in urban agriculture: An opportunity towards environmental sustainability and water use efficiency? Journal of Cleaner Production Volume 261, 121213. DOI: 10.1016/j.jclepro.2020.121213

312.Rufí-Salís M, Petit-Boix A, Villalba G, Ercilla-Montserrat M, Sanjuan-Delmás D, Parada F, Arcas V, Muñoz-Liesa J, Gabarrell X (2020b) Identifying eco-efficient year-round crop combinations for rooftop greenhouse agriculture. The International Journal of Life Cycle Assessment 25:564–576. DOI:10.1007/s11367-019-01724-5

313.Rumble H & Gange AC (2017) Microbial inoculants as a soil remediation tool for extensive greenroofs. Ecological Engineering 102, 188–198. DOI:10.1016/j.ecoleng.2017.01.025

314.Rusyn IB & Hamkalo KR (2019) Use of *Carex hirta* in electro-biotechnological systems on green roofs. Regulatory Mechanisms in Biosystems 10 (1), 39-44. DOI:10.15421/021906

315.Salman INA & Blaustein L (2018) Vegetation cover drives arthropod communities in Mediterranean/subtropical green roof habitats. Sustainability 10, 4209; doi:10.3390/su10114209

316.Sangkakool T & Techato T (2017) Environmental benefits of air plant green roofs in hot and humid climate. Journal of Engineering and Applied Science 12 (5), 6939-6946. ISSN 1816-949X

317.Santos PLF & Castilho RMM (2018) Floriferous herbaceous and substrates for use on extensive green roofs. Ornamental Horticulture 24 (3) DOI: http://dx.doi.org/10.14295/oh.v24i3.1251

318.Savi T, Andri S, Nardini A (2013) Impact of different green roof layering on plant water status and drought survival. Ecological Engineering 57, 188– 196. DOI:10.1016/j.ecoleng.2013.04.048

319.Savi T, Marin M, Boldrin D, Incerti G, Andri S, Nardini A (2014) Green roofs for a drier world: Effects of hydrogel amendment on substrate and plant water status. Science of the Total Environment 490, 467–476. DOI: 10.1016/j.scitotenv.2014.05.020

320.Savi T, Boldrin D, Marin M, Love VL, Andri S, Tretiach M, Nardini A (2015) Does shallow substrate improve water status of plants growing ongreen roofs? Testing the paradox in two sub-Mediterranean shrubs. Ecological Engineering 84, 292–300. 10.1016/j.ecoleng.2015.09.036

321.Savi T, Borgo AD, Love VL, Andri S, Tretiach M, Nardini A (2016) Drought versus heat: What's the major constraint on Mediterranean green roof plants? Science of the Total Environment 566–567, 753–760. DOI: 10.1016/j.scitotenv.2016.05.100

322.Schiappacasse F, Rodríguez E, Nektarios PA, Gaete KM, Maturana LD (2017) Growth of the Chilean plants Haplopappus macrocephalus and Selliera radicans on an extensive modular green roof system under three irrigation regimes. IDESIA (Chile) Volumen 35, Nº 3. http://dx.doi.org/10.4067/S0718-34292017005000203.

323.Schindler BY, Blaustein L, Lotan R, Shalom H, Kadas GJ, Seifan M (2018) Green roof and photovoltaic panel integration: Effects on plant and arthropod diversity and electricity production. Journal of Environmental Management 225, 288–299. DOI: 10.1016/j.jenvman.2018.08.017

324.Schindler BY, Blaustein L, Vasl A, Kadas GJ, Seifan M (2019) Cooling effect of *Sedum sediforme* and annual plants on green roofs in a Mediterranean climate. Urban Forestry & Urban Greening 38, 392–396. DOI:10.1016/j.ufug.2019.01.020

325.Schneider A, Fusco M, Bousselot J (2014) Observations on the survival of 112 plant taxa on a green roof in a semi-arid climate. Journal of Living Architecture. 1(5): 10-30. http://greenroofs.org/resources/JOLA2014Volume1(Issue5)Schneider(etal).pdf

326.Schrieke D, Williams NSG, Farrell C (2023a) Response of spontaneous plant communities to *Sedum mexicanum* cover and water availability in green roof microcosms. Land, 12, 1239. https://doi.org/10.3390/ land12061239

327.Schröder R, Mohri M, Kiehl K (2019) AMF inoculation of green roof substrate improves plant performance but reduces drought resistance of native dry grassland species. Ecological Engineering 139 105583. DOI:10.1016/j.ecoleng.2019.105583

328.Schrieke D, Szota C, Williams NSG, Farrell C (2023b) Evaluating the effectiveness of spontaneous vegetation for stormwater mitigation on green roofs. Science of the Total Environment 898, 165643. https://doi.org/10.1016/j.scitotenv.2023.165643

329.Schröder R & Kiehl K (2020) Extensive roof greening with native sandy dry grassland species: Effects of different greening methods on vegetation development over four Years. Ecological Engineering 145, 105728. DOI:10.1016/j.ecoleng.2020.105728

330.Schroder R & Kiehl K (2021) Testing standard growth substrates for establishing native dry sandy grassland species on extensive green roofs in Northern Germany. Basic and Applied Ecology 56, 181-191. https://doi.org/10.1016/j.baae.2021.07.010

331.Schroll E, Lambrinos JG, Sandrock D (2011) An evaluation of plant selections and irrigation requirements for extensive green roofs in the Pacific northwestern United States. HortTechnology 21 (3). DOI:10.21273/HORTTECH.21.3.314

332.Schultz I, Sailor DJ, Starry O (2018) Effects of substrate depth and precipitation characteristics on stormwater retention by two green roofs in Portland OR.

Journal of Hydrology: Regional Studies 18, 110-118. https://doi.org/10.1016/j.ejrh.2018.06.008

333.Schweitzer O & Erell E (2014) Evaluation of the energy performance and irrigation requirements ofextensive green roofs in a water-scarce Mediterranean climate. Energy and Buildings 68, 25–32.

334.Sendo T, Kanechi M, Uno Y, Inagaki N (2010) Evaluation of growth and green coverage of ten ornamental species for planting as urban rooftop greening. J. Japan. Soc. Hort. Sci. 79 (1): 69–76. DOI:10.2503/jjshs1.79.69

335.Seyedabadi MR, Eicker U, Karimi S (2021) Plant selection for green roofs and their impact on carbon sequestration and the building carbon footprint. Environmental Challenges 4, 100119. https://doi.org/10.1016/j.envc.2021.100119

336.Seyedabadi MR, Karrabi M, Nabati J (2022) Investigating green roofs' CO2 sequestration with coldand drought-tolerant plants (a short- and long-term carbon footprint view). Environmental Science and Pollution Research, 29:14121–14130. https://doi.org/10.1007/s11356-021-16750-w

337.Shade J, Lidelow S, Lonnqvist J (2021) The thermal performance of a green roof on a highly insulated building in a sub-arctic climate. Energy & Buildings 241, 110961. https://doi.org/10.1016/j.enbuild.2021.110961

338.Sherk JT, Fu W, Neal JC (2020) Site conditions, maintenance costs, and plant performance of 10 extensive green roofs in the Research Triangle Area of Central North Carolina. HortTechnology https://doi.org/10.21273/HORTTECH04565-20

339.Shetty NH, Elliott RM, Wang M, Palmer MI, Culligan PJ (2022) Comparing the hydrological performance of an irrigated native vegetation green roof with a conventional Sedum spp. green roof in New York City. PLoS ONE 17(4): e0266593. https://doi.org/10.1371/journal.pone.0266593

340.Shi Y, Wu R, Chen M, Jin H, Bao Z, Yan H, Yang F (2017) Understanding Perceptions of Plant Landscaping in LID: Seeking a Sustainable Design and Management Strategy. J. Sustainable Water Built Environ., 3(4): 05017003. doi: 10.1061/JSWBAY.0000834.

341.Silva B, Mantovani A, Mantuano DG, Rola SM, Barbosa MC (2018) Evaluating plant species suitability for a substrate-free tropical green roof. OnLine Journal of Biological Sciences, 18 (4): 401.423 DOI: 10.3844/ojbsci.2018.401.423

342.Sims AW, Robinson CE, Smart CC, Voogt JA, Hay GF, Lundholm JT, Powers B, O'Carroll DM (2016) Retention performance of green roofs in three different climate regions. Journal of Hydrology 542, 115–124. http://dx.doi.org/10.1016/j.jhydrol.2016.08.055

343.Sims AW, Robinson CE, Smart CC, O'Carroll DM (2019) Mechanisms controlling green roof peak flow rate attenuation. Journal of Hydrology 577, 123972. https://doi.org/10.1016/j.jhydrol.2019.123972

344.Simoes N, Almeida R, Tadeu A, Brett M, Almeida J (2020) Comparison between cork-based and conventional green roof solutions. Building and Environment 175, 106812. DOI:10.1016/j.buildenv.2020.106812

345.Sitawati, Baskara M, Wattimena CF (2022) Effect of vermicompost and NPK application techniques on Marigold growth and flowering on vertical pipes. Asian J. Plant Sci., 21: 257-263. DOI: 10.3923/ajps.2022.257.263

346.Skala V, Dohnal M, Votrubová J, Jelínková V (2019) The use of simple hydrological models to assess outflow of two green roofs systems. Soil and Water Research, 14 (2): 94–103. DOI: 10.17221/138/2018-SWR

347.Skala V, Dohnal M, Votrubova J, Vogel T, Dusek J, Sacha J, Jelinkov V (2020) Hydrological and thermal regime of a thin green roof system evaluated by

physically-based model. Urban Forestry & Urban Greening 48, 126582. DOI:10.1016/j.ufug.2020.126582

348.Snehota M, Hanzlikova J, Sobotkova M, Moravcik P (2021) Water and thermal regime of extensive green roof test beds planted with sedum cuttings and sedum carpets. Journal of Soils and Sediments 21, 2089-2101. https://doi.org/10.1007/s11368-020-02778-x

349.Song X, Hu X, Kong L, Zhang Z, Li X, Chen Q, Li1 G, Bi H (2023) Growth and physiological responses of six species of Crassulaceae in green roof to consecutive water deficit conditions. Pol. J. Environ. Stud. Vol. 32, No. 3, 2833-2843. DOI: 10.15244/pjoes/161869

350.Soni L, Szota C, Fletcher TD, Farrell C (2023) Influence of green roof plant density and redirecting rainfall via runoff zones on rainfall retention and plant drought stress. Science of the Total Environment 889, 164043. http://dx.doi.org/10.1016/j.scitotenv.2023.164043

351.Soulis KX, Ntoulas N, Nektarios PA, Kargas G (2017) Runoff reduction from extensive green roofs having different substrate depth and plant cover. Ecological Engineering 102, 80–89. DOI:10.1016/j.ecoleng.2017.01.031

352.Speak AF, Rothwell JJ, Lindley SJ, Smith CL (2012) Urban particulate pollution reduction by four species of green roof vegetation in a UK city. Atmospheric Environment 61, 283-293. https://doi.org/10.1016/j.atmosenv.2012.07.043

353.Starry O, Lea-Cox JD, Kim J, van Iersel MV (2014) Photosynthesis and water use by two Sedum species in green roof substrate. Environmental and Experimental Botany, http://dx.doi.org/10.1016/j.envexpbot.2014.05.014

354.Suárez-Cáceres GP, Mejía-Sampedro DK, Fernández-Cañero R, Loges V, Pérez-Urrestarazu L (2023) Establishment and development of ornamental grasses on

green roofs and living walls. Landscape and Ecological Engineering, 19:123–136. https://doi.org/10.1007/s11355-022-00527-5

355.Suszanowicz D & Kolasa-Wiecek A (2019) The impact of green roofs on the parameters of the environment in urban areas—review. Atmosphere 10, 792; doi:10.3390/atmos10120792

356.Sutton RK, Harrington JA, Skabelund L, MacDonaghd P, Coffmane RR, Koch G (2011) Prairie-based green roofs: literature, templates, and analogs. Journal of Green Building (7) 1. DOI:10.3992/jgb.7.1.143

357.Szota C, Farrell C, Williams NSG, Arndt SK, Fletcher TD (2017) Drought-avoiding plants with low water use can achieve high rainfall retention without jeopardising survival on green roofs, Science of the Total Environment 603–604, 340–351. DOI: 10.1016/j.scitotenv.2017.06.061

358.Tan CL, Tan PY, Wong NH, Takasuna H, Kudo T, Takemasa Y, Lim CVJ, Chua HXV (2017) Impact of soil and water retention characteristics on green roof thermal performance, Energy and Buildings http://dx.doi.org/10.1016/j.enbuild.2017.01.011

359.Tan JKN, Belcher RN, Tan HTW, Menz S, Schroepfer T (2021) The urban heat island mitigation potential of vegetation depends on local surface type and shade. Urban Forestry & Urban Greening, 62 127128. https://doi.org/10.1016/j.ufug.2021.127128

360.Tang M & Zheng X (2019) Experimental study of the thermal performance of an extensive green roof on sunny summer days. Applied Energy 242, 1010–1021. DOI: 10.1016/j.apenergy.2019.03.153

361.Tapia NF, Rojas C, Bonilla CA, Vargas IT (2017a) Evaluation of Sedum as driver for plant microbial fuel cells in a semi-arid green roof ecosystem. Ecological Engineering 108, 203–210. DOI:10.1016/j.ecoleng.2017.08.017

362.Tapia NF, Rojas C, Bonilla CA, Vargas IT (2018b) A new method for sensing soil water content in green roofs using plant microbial fuel cells. Sensors 18, 71; doi:10.3390/s18010071

363.Tassoula L, Papafotiou M, Liakopoulos G, Karga G (2015) Growth of the native xerophyte *Convolvulus cneorum* L. on an extensive Mediterranean green roof under different substrate types and irrigation regimens. HORTSCIENCE 50(7):1118–1124. DOI:10.21273/HORTSCI.50.7.1118

364.Tassoula L, Papafotiou M, Liakopoulos G, Kargas G (2021) Water use efficiency, growth and anatomic-physiological parameters of Mediterranean xerophytes as affected by substrate and irrigation on a green roof. Notulae Botanicae Horti Agrobotanici Cluj-Napoca, 49, 2, 12283. DOI:10.15835/nbha49212283

365.Teemusk A, Kull A, Kanal A, Mander U (2019) Environmental factors affecting greenhouse gas fluxes of green roofs in temperate zone. Science of the Total Environment 694 133699. DOI: 10.1016/j.scitotenv.2019.133699

366.Thomaidi V, Petousi I, Kotsia D, Kalogerakis N, Fountoulakis MS (2022) Use of green roofs for greywater treatment: Role of substrate, depth, plants, and recirculation. Science of the Total Environment 807, 151004. https://doi.org/10.1016/j.scitotenv.2021.151004

367.Thuring CE & Dunnett NP (2019) Persistence, loss, and gain: Characterising mature green roof vegetation by functional composition. Landscape and Urban Planning 185, 228–236. https://doi.org/10.1016/j.landurbplan.2018.10.026

368.Toland DC, West CP, Boyer ME (2012) Media Composition Influences Green Roof Plant Viability In The Ozark Highlands. Journal of Green Building vol 7 (4). DOI:10.3992/jgb.7.4.73

369.Toland DC, Boyer ME, McDonald GV, West CP, Haggard BE (2014) Plants influenced by growing media and compost addition on mock green roofs within the Ozark Highlands. Journal of Green Building vol 9 (1). DOI:10.3992/1943-4618-9.1.130

370.Tran S, Lundholm JT, Staniec MA, Robinson CE, Smart CC, Voogt JA, O'Carroll DM (2018) Plant survival and growth on extensive green roofs: A distributed experiment in three climate regions. Ecological Engineering vol 127: 494-503. DOI:10.1016/j.ecoleng.2018.09.027

371.Tuttolomondo T, Fascella G, Licata M, Schicchi R, Gennaro MC, La Bella S, Leto C, Aprile S (2018) Studies on Sedum taxa found in Sicily (Italy) for Mediterranean extensive green roofs. Italian Journal of Agronomy, vol 13:1077. https://doi.org/10.4081/ija.2018.1077

372.Vahdati N, Tehranifar A, Kazemi F (2017) Assessing chilling and drought tolerance of different plant genera on extensive green roofs in an arid climate region in Iran. Journal of Environmental Management 192, 215-223. DOI:10.1016/j.jenvman.2017.01.027

373.Van PTH, Tin NT, Hien VTD, Quan TM, Thanh BX, Hang VT, Tuc DQ, Dan NP, Khoa LV, Phu VL, Son NT, Luong ND, Kwon E, Park C, Jung J, Yoon I, Lee S (2015) Nutrient removal by different plants in wetland roof systems treating domestic wastewater, Desalination and Water Treatment, 54:4-5, 1344-1352, DOI: 10.1080/19443994.2014.915767

374.Vandegrift DA, Rowe DB, Cregg BM, Liang D (2019) Effect of substrate depth on plant community development on a Michigan green roof. Ecological Engineering 138, 264–273. DOI:10.1016/j.ecoleng.2019.07.032

375.Van Mechelen C, Van Meerbeek K, Dutoit T, Hermy M (2015a) Functional diversity as a framework for novel ecosystem design: The example of extensive green roofs. Landscape and Urban Planning 136, 165–173. DOI:10.1016/j.landurbplan.2014.11.022

376.Van Mechelen C, Dutoit T, Hermy M (2015b) Vegetation development on different extensive green roof types in a Mediterranean and temperate maritime climate. Ecological Engineering 82, 571–582. DOI:10.1016/j.ecoleng.2015.05.011

377.Van Mechelen C, Dutoit T, Kattge J, Hermy M (2014a) Plant trait analysis delivers an extensive list of potential green roofspecies for Mediterranean France. Ecological Engineering 67 (2014) 48–59. DOI:10.1016/j.ecoleng.2014.03.043

378.Van Mechelen C, Dutoit T, Hermy M (2014b) Mediterranean open habitat vegetation offers great potential for extensive green roof design. Landscape and Urban Planning 121, 81– 91. DOI:10.1016/j.landurbplan.2013.09.010

379.Vannucchi F, Pini R, Scatena M, Benelli G, Canale A, Bretzel F (2018) Deinking sludge in the substrate reduces the fertility and enhances the plant species richness of extensive green roofs. Ecological Engineering 116, 87–96. DOI:10.1016/j.ecoleng.2018.02.027

380.Vannucchi F, Buoncristiano A, Scatena M, Caudai C, Bretzel F (2022) Low productivity substrate leads to functional diversification of green roof plant assemblage. Ecological Engineering 176, 106547. https://doi.org/10.1016/j.ecoleng.2022.106547

381. Vanstockem J, Somers B, Hermy M (2019) Weeds and gaps on extensive green roofs: ecological insights and recommendations for design and maintenance. Urban Forestry and amp; Urban Greening, doi: https://doi.org/10.1016/j.ufug.2019.126484

382. Varela A, Sandoval-Albán A, Munoz M, Gómez AG, Bogoya JM, Combariza G (2021) Evaluation of green roof structures and substrates for Lactuca sativa L. in tropical conditions. Urban Forestry & Urban Greening, 60, 127063. https://doi.org/10.1016/j.ufug.2021.127063

383. Varela-Stasinopoulou DS, Nektarios PA, Ntoulas N, Trigas P, Roukounakis GI (2023) Sustainable growth of medicinal and aromatic Mediterranean plants growing as communities in shallow substrate urban green roof systems. Sustainability, 15, 5940. https:// doi.org/10.3390/su15075940

384. Varela-Stasinopoulou DS, Nektarios PA, Tsanakas GF, Ntoulas N, Roukounakis GI, Economou AS (2023) Impact of substrate depth and irrigation regime on growth, flowering, and physiological indices of Greek sage (*Salvia fruticosa* Mill.) grown on urban extensive green roof systems. Ecological Engineering 186, 106816. https://doi.org/10.1016/j.ecoleng.2022.106816

385. Vasiliu-Oromulu L, Bărbuceanu D, Vierbergen G (2012) Thysanoptera in a green roof microecosystem in Bucharest, Romania. Acta Phytopathologica et Entomologica Hungarica 47 (1): 161–166. DOI: https://doi.org/10.1556/aphyt.47.2012.1.20

386. Vasl A, Shalom H, Kadas GJ, Blaustein L (2017) Sedum—Annual plant interactions on green roofs: Facilitation, competition, and exclusion. Ecological Engineering 108: 318–329. DOI:10.1016/j.ecoleng.2017.07.034

387. Vasl A, Schindler BY, Kadas GJ, Blaustein L (2019) Fine-scale substrate heterogeneity in green roof plant communities: The constraint of size. Ecology and Evolution, 9: 11557–11568. doi: 10.1002/ece3.5517

388. Vaz Monteiro M, Blanusa T, Verhoef A, Richardson M, Hadley P, Cameron RWF (2017) Functional green roofs: Importance of plant choice in

maximisingsummertime environmental cooling and substrate insulationpotential. Energy and Buildings 141, 56–68. DOI:10.1016/j.enbuild.2017.02.011

389.Vera S, Viecco M, Jorquera H (2021) Effects of biodiversity in green roofs and walls on the capture of fine particulate matter. Urban Forestry & Urban Greening, 63, 127229. https://doi.org/10.1016/j.ufug.2021.127229

390.Vestrella A, Biel C, Savè R, Bartoli F (2018) Mediterranean Green Roof Simulation in Caldes de Montbui (Barcelona): Thermal and Hydrological Performance Test of Frankenia laevis L., Dymondia margaretae Compton and Iris lutescens Lam. Appl. Sci. 8, 2497; doi:10.3390/app8122497

391.Vestrella A, Savé R, Biel C (2015) Performance of Two Simulated Green Roofs in the Mediterranean Area. Journal of Agricultural Science; Vol. 7, No. 11. DOI:10.5539/jas.v7n11p59

392.Viecco M, Vera S, Jorquera H, Bustamante W, Gironás J, Dobbs C, Leiva E (2018) Potential of Particle Matter Dry Deposition on Green Roofs and LivingWalls Vegetation for Mitigating Urban Atmospheric Pollution in Semiarid Climates. Sustainability 10, 2431; doi:10.3390/su10072431

393.Vijayaraghavan K & Joshi UM (2014) Can green roof act as a sink for contaminants? A methodological study to evaluate runoff quality from green roofs. Environmental Pollution 194, 121-129. DOI: 10.1016/j.envpol.2014.07.021

394.Vijayaraghavan K & Raja FD (2014) Design and development of green roof substrate to improve runoff water quality: Plant growth experiments and adsorption. Water Research 63, 94-101. DOI: 10.1016/j.watres.2014.06.012

395.Vijayaraghavan K, Arockiaraj J, Kamala-Kannan S (2017) Portulaca grandiflora as green roof vegetation: Plant growth and phytoremediation experiments. Int J Phytoremediation, 19 (6): 537-544. doi: 10.1080/15226514.2016.1267699.

396.Villalba GA, Rosatto HG, Bienvenido F, Flores-Parra IM, Botta GF, Laureda DA, Perez DA (2017) Techos verdes. Contribución de *Carpobrotus acinaciformis* al manejo integrado de escurrimientos superficiales urbanos. Revista de la Facultad de Ciencias Agrarias 49 (2): 169-181. https://revistas.uncu.edu.ar/ojs3/index.php/RFCA/article/view/3073/2223

397.Vinson KL & Zheng Y (2013) Green Roof Plant Suitability Analysis for Northern Climates. HortTechnology 23 (5). DOI:10.21273/HORTTECH.23.5.563

398.Vo TD, Bui XT, Nguyen DD, Nguyen VT, Ngo HH, Guo W, Nguyen PD, Nguyen CN, Lin C (2017) Wastewater treatment and biomass growth of eight plants for shallow bed wetland roofs, Bioresource Technology, doi: https://doi.org/10.1016/j.biortech.2017.09.194

399.Volder A & Dvorak B (2014) Event size, substrate water content and vegetation affect storm water retentionefficiency of an un-irrigated extensive green roof system in Central Texas. Sustainable Cities and Society 10, 59–64. DOI:10.1016/j.scs.2013.05.005

400.Voyde E, Fassman E, Simcock R, Wells J (2010) Quantifying Evapotranspiration Rates for New Zealand Green Roofs. Journal Of Hydrologic Engineering 15, 395-403. DOI:10.1061/(ASCE)HE.1943-5584.0000141

401.Walker EA & Lundholm JT (2017) Designed habitat heterogeneity on green roofs increases seedling survival but not plant species diversity. doi: 10.1111/1365-2664.12970

402.Wang X, Tian Y, Zhao X (2017) The influence of dual-substrate-layer extensive green roofs on rainwater runoff quantity and quality. Science of the Total Environment 592, 465–476. http://dx.doi.org/10.1016/j.scitotenv.2017.03.124

403.Wang H, Zhang K, Gan L, Liu J, Mei G (2020) Expansive soil-biochar-root-water bacteria interaction: Investigation on crack development, water management and plant growth in green infrastructure. International Journal of Damage Mechanics 0(0) 1–23. DOI:10.1177/1056789520974416

404.Wang J, Mei G, Garg A, Chen D, Liu N (2023) A simplified model for analyzing rainwater retention performance and irrigation management of green roofs with an inclusion of water storage layer. Journal of Environmental Management 326, 116740. https://doi.org/10.1016/j.jenvman.2022.116740

405.Weiss O, Minixhofer P, Scharf B, Pitha U (2021) Equation for Calculating Evapotranspiration of Technical Soils for Urban Planting. Land, 10, 622. https://doi.org/10.3390/land10060622

406.Whittinghill LJ & Rowe DB (2011) Salt tolerance of common green roof and green wall plants. Urban Ecosyst 14:783–794 DOI 10.1007/s11252-011-0169-4

407.Whittinghill LJ, Rowe DB, Cregg BM (2013) Evaluation of Vegetable Production on Extensive Green Roofs, Agroecology and Sustainable Food Systems, 37:4, 465-484, DOI: 10.1080/21683565.2012.756847

408.Whittinghill LJ, Rowe DB, Schutzki R, Cregg BM (2014a) Quantifying carbon sequestration of various green roof and ornamental landscape systems. Landscape and Urban Planning 123, 41– 48. DOI:10.1016/j.landurbplan.2013.11.015

409.Whittinghill LJ, Rowe DB, Andresen JA, Cregg BM (2041b) Comparison of stormwater runoff from sedum, native prairie, and vegetable producing green roofs. Urban Ecosyst, 18:13–29 DOI 10.1007/s11252-014-0386-8

410.Williams NSG, Hughes RE, Jones NM, Bradbury DA, Rayner JP (2010) The Performance of Native and Exotic Species for Extensive Green Roofs in Melbourne,

Australia. Proc. IInd Int’l Conf. on Landscape and Urban Hort. Eds.: G. Prosdocimi Gianquinto and F. Orsini Acta Hort. 881. DOI:10.17660/ActaHortic.2010.881.113

411.Williams NSG, Rayner JP, Lee KE, Fletcher TD, Chen D, Szota C, Farrell C (2016) Developing Australian green roofs: overview of a 5-year research program. Acta Hortic. 1108. DOI 10.17660/ActaHortic.2016.1108.46

412. Wu T (2019) Abundance and diversity of pollinators on green roofs are affected by environmental factors. Earth and Environmental Science 358. doi:10.1088/1755-1315/358/2/022053

413.Xie L, Lehva¨virta S, Timonen S, Kasurinen J, Niemikapee J, Valkonen JPT (2018) Speciesspecific synergistic effects of two plant growth— Promoting microbes on green roof plant biomass and photosynthetic efficiency. PLoS ONE 13(12): e0209432. https://doi.org/10.1371/journal. pone.0209432

414.Xie L, Lehvävirta S, Valkonen JPT (2020) Case study: Planting methods and beneficial substrate microbes effect on the growth of vegetated roof plants in Finland. Urban Forestry & Urban Greening 53, 126722 DOI:10.1016/j.ufug.2020.126722

415.Xu H, Yeum K, Yoon YH, Ju JH (2018) Effect of hydrophilic polymer in three green roof substrates on growth, flower development, and overwintering of *Agastache rugosa* (korean mint) without irrigation. Applied Ecology and Environmental Research 16(5):5503-5516. DOI: http://dx.doi.org/10.15666/aeer/1605_55035516

416.Xue1 M & Farrell C (2020) Use of organic wastes to create lightweight green roof substrates with increased plant-available water. Urban Forestry & Urban Greening 48, 126569. DOI:10.1016/j.ufug.2019.126569

417.Yacob MNM, Kasmin H, Hashim MIH (2021) Estimating Carbon Sequestration of Green Roof Plants in Tropical Climate. International Journal Of

Integrated Engineering Vol. 13 (3), 200-206. DOI: https://doi.org/10.30880/ijie.2021.13.03.024

418.Yalcinalp E, Ozveren S, Meral A, Pulatkan M, Akbulut S (2017) Habitat Effect on Urban Roof Vegetation. Sustainability 9, 1985; doi:10.3390/su9111985

419.Yalçinalp E, Şivil M, Meral A, Demir Y (2019) Green roof plant responses to greywater irrigation. Applied Ecology and Environmental Research 17(2):3667-3680. DOI:10.15666/aeer/1702_36673680

420.Yang M, Dong W, Cheng R, Wang H, Zhao Z, Wang F, Wang Y (2022) Effect of highly efficient substrate modifier, super-absorbent polymer, on the performance of the green roof. Science of the Total Environment 806, 150638. https://doi.org/10.1016/j.scitotenv.2021.150638

421.Ye J, Liu C, Zhao Z, Li Y, Yu S (2013) Heavy metals in plants and substrate from simulated extensive green roofs. Ecological Engineering 55, 29– 34. DOI:10.1016/j.ecoleng.2013.02.012

422.Yee EG, Callahan HS, Griffin KL, Palmer MI, Lee S (2022) Seasonal patterns of native plant cover and leaf trait variation on New York City green roofs. Urban Ecosystems, 25, 229-240. https://doi.org/10.1007/s11252-021-01134-2

423.Yıldırım S, Özburak Ç, Özden Ö (2023) Green roofs, vegetation types, impact on the thermal effectiveness: an experimental study in Cyprus. Sustainability, 15, 2807. https://doi.org/10.3390/ su15032807

424.Yin H, Kong F, Dronova I (2019) Hydrological performance of extensive green roofs in response to diferent rain events in a subtropical monsoon climate. Landscape and Ecological Engineering, 15:297–313. https://doi.org/10.1007/s11355-019-00380-z

425.Young T, Cameron DD, Sorrill J, Edwards T, Phoenix GK (2014) Importance of different components of green roof substrate on plantgrowth and physiological performance. Urban Forestry & Urban Greening 13 (3): 507-516. DOI:10.1016/j.ufug.2014.04.007

426.Young T, Cameron DD, Phoenix GK (2015b) Using AMF inoculum to improve the nutritional status of Prunellavulgaris plants in green roof substrate during establishment. Urban Forestry & Urban Greening 14, 959–967. DOI:10.1016/j.ufug.2015.08.012

427.Young TM, Cameron DD, Phoenix GK (2017) Increasing green roof plant drought tolerance through substrate modification and the use of water retention gels, Urban Water Journal, 14:6, 551-560, DOI: 10.1080/1573062X.2015.1036761

428.Yuan J, Dunnett N, Stovin V (2017) The influence of vegetation on rain garden hydrological performance, Urban Water Journal, 14:10, 1083-1089, DOI: 10.1080/1573062X.2017.1363251

429.Zanin G & Bortolini L (2020) Performance of three different native plant mixtures for extensive green roofs in a humid subtropical climate context. Water, 12, 3484; doi:10.3390/w12123484

430.Zhang H, Lu S, Wu J, Jiang Y, Lu Y, Zhao H (2014) Effect of substrate depth on 18 non-succulent herbaceous perennials for extensive green roofs in a region with a dry spring. Ecological Engineering 71, 490–500. DOI:10.1016/j.ecoleng.2014.07.033

431.Zhang Z, Szota C, Fletcher TD, Williams NSG, Werdin J, Farrell C (2018) Influence of plant composition and water use strategies on green roof stormwater retention. Science of the Total Environment 625, 775–781. DOI: 10.1016/j.scitotenv.2017.12.231

432.Zhang Z, Szota C, Fletcher TD, Williams NSG, Farrell C (2019) Green roof storage capacity can be more important than evapotranspiration for retention performance. Journal of Environmental Management 232, 404–412. DOI: 10.1016/j.jenvman.2018.11.070

433.Zhang G, He BJ, Dewancker BJ (2020) The maintenance of prefabricated green roofs for preserving cooling performance: A field measurement in the subtropical city of Hangzhou, China. Sustainable Cities and Society, doi: https://doi.org/10.1016/j.scs.2020.102314

434.Zhang H, Fan X, Ren L, Jiang Y, Wu J, Zhao H (2021a) Crassulacean plant succession over eight years on an unirrigated green roof in Beijing, Urban Forestry and amp; Urban Greening, doi: https://doi.org/10.1016/j.ufug.2021.127189

435.Zhang H, Lu S, Fan X, Wu J, Jiang Y, Ren L, Wu J, Zhao (2021b) Is sustainable extensive green roof realizable without irrigation in a temperate monsoonal climate? A case study in Beijing. Science of the Total Environment 753, 142067. https://doi.org/10.1016/j.scitotenv.2020.142067

436.Zhang K, Garg A, Mei G, Jiang M, Wang H, Huang S, Gan L (2022) Thermal performance and energy consumption analysis of eight types of extensive green roofs in subtropical monsoon climate. Building and Environmet 216, 108982. https://doi.org/10.1016/j.buildenv.2022.108982

437.Zhao M, Tabares-Velasco PC, Srebric J, Komarneni S, Berghage R (2014) Effects of plant and substrate selection on thermal performance of green roofs during the summer. Building and Environment 78: 199-211. DOI:10.1016/j.buildenv.2014.02.011

438.Zhen M, Zou W, Ding W (2022) Cooling efect of roof greening with water misting in a cold region during the summer. Environment, Development and Sustainability, 24, 7093-7114. https://doi.org/10.1007/s10668-021-01740-2

439.Zheng X, Yang Z, Yang J, Tang M, Feng C (2022) An experimental study on the thermal and energy performance of self-sustaining green roofs under severe drought conditions in summer. Energy & Buildings 261, 111953. https://doi.org/10.1016/j.enbuild.2022.111953
