# Supplementary material for: Life at the Top: Extensive Green Roof Plant Species and Their Traits for Urban Use
Source: Plants (Basel). 2025 Feb 27;14(5):735. doi: 10.3390/plants14050735 (PMC11902263; doi:10.3390/plants14050735)
Supplement: Supplementary file 1 [file plants-14-00735-s001.zip › S4 - species per climate list - R1.pdf]

Supplementary Material S4

Table S4.1. Selected species most frequently found in publications from continental climate as being adequate for green roof use. For those species recognized as being of full-scale consolidated use, corresponding references in Supplementary Material 2 are indicated in the last column. All plant names were checked in IPNI.

**Species name**	**Frequency**	**Family**	**Full-scale use ***
Sedum spurium M.Bieb.	45	Crassulaceae	13, 204, 253
Sedum kamtschaticum Fisch.	30	Crassulaceae	110, 204
Sedum acre L.	26	Crassulaceae	50, 204, 380
Sedum album L.	24	Crassulaceae	50, 110, 204
Sedum rupestre L.	21	Crassulaceae	110, 253, 304
Sedum sexangulare L.	15	Crassulaceae	110, 204, 253
Allium cernuum Roth	9	Amaryllidaceae	356
Solidago bicolor L.	8	Asteraceae	141
Sedum lineare Thunb.	7	Crassulaceae	196, 402, 420
Achillea millefolium L.	7	Asteraceae	49, 70, 204
Schizachyrium scoparium (Michx.) Nash	6	Poaceae	356, 422
Campanula rotundifolia L.	6	Campanulaceae	204, 206, 356
Danthonia spicata (L.) Roem. & Schult.	6	Poaceae	-
Monarda fistulora L.	6	Lamiaceae	-
Sedum roseum (L.) Scop.	6	Crassulaceae	-
Aquilegia canadenses L.	6	Ranunculaceae	-
Allium schoenoprasum L.	5	Amaryllidaceae	174
Trifolium repens L.	5	Fabaceae	50, 204, 326
Sedum hybridum L.	5	Crassulaceae	253
Sedum hispanicum L.	5	Crassulaceae	253
Sedum telephium L.	5	Crassulaceae	-
Echinacea purpurea (L.) Moench	5	Asteraceae	-
Sedum aizoon L.	5	Crassulaceae	-
Sporonolus heterolepis (Gray) A.Gray	5	Poaceae	-
Festuca rubra L.	4	Poaceae	49, 141, 356
Erigeron canadensis L.	4	Asteraceae	-

Taraxacum campylodes G.E.Haglund	4	Asteraceae	-
Rudbeckia hirta L.	4	Asteraceae	-
Symphyotrichum novae-angliae (L.) G.L.Nesom	4	Asteraceae	-
Medicago lupulina L.	4	Fabaceae	-

* = SM2 Reference number

Table S4.2. Selected species most frequently found in publications from dry climate as being adequate for green roof use. For those species recognized as being of full-scale consolidated use, corresponding references in Supplementary Material 2 are indicated in the last column. All names were checked in IPNI.

**Species name**	**Frequency**	**Family**	**Full-scale use ***
Sedum rupestre L.	12	Crassulaceae	110, 253, 304
Sedum acre L.	10	Crassulaceae	50, 204, 380
Sedum album L.	7	Crassulaceae	50, 110, 204
Sedum spurium M.Bieb.	6	Crassulaceae	13, 204, 253
Phyla nodiflora (L.) Greene	5	Verbenaceae	-
Delosperma cooperi (Hook.f.) L.Bolus	4	Aizoaceae	-
Sedum lineare Thunb.	3	Crassulaceae	196, 402, 420
Portulaca grandiflora Hook.	3	Portulacaceae	50, 109
Ophiopogon japonicus (Thunb.) Ker Gawl.	3	Asparagaceae	-
Mesembryanthemum cordifolium L.f.	3	Aizoaceae	-
Chondrosum gracile Kunth.	3	Poaceae	-
Iris tectorum Maxim.	3	Iridaceae	-
Hylotelephium erythrostictum (Miq.) H. Ohba	3	Crassulaceae	-
Delosperma nubigenum (Schltr.) L.Bolus	3	Aizoaceae	-
Eschscholzia californica Cham.	3	Papaveraceae	-
Sporonolus heterolepis (Gray) A.Gray	2	Poaceae	-
Sedum kamtschaticum Fisch.	2	Crassulaceae	110, 204
Festuca rubra L.	2	Poaceae	49, 141, 356
Talinum calycinum Engelm. in Wisl.	2	Talinaceae	356
Sedum hybridum L.	2	Crassulaceae	253

Allium cernuum Roth	2	Amaryllidaceae	356
Lavandula angustifolia Mill.	2	Lamiaceae	222, 389
Symphyotrichum laeve (L.) Á.Löve & D.Löve	2	Asteraceae	-
Lactuca sativa L.	2	Asteraceae	-
Sedum spathulifolium Hook.	2	Crassulaceae	-
Buchloe dactyloides (Nutt.) Engelm.	2	Poaceae	-
Chrysocephalum apiculatum (Labill.) Steetz	2	Asteraceae	-
Aquilegia canadensis L.	2	Ranunculaceae	-
Lampranthus spectabilis (Haw.) N.E. Br.	2	Aizoaceae	-
Nussella tenuissima (Trin.) Barkworth	2	Poaceae	-

* = SM2 Reference number

Table S4.3. Selected species most frequently found in publications from temperate climate as being adequate for green roof use. For those species recognized as being of full-scale consolidated use, corresponding references in Supplementary Material 2 are indicated in the last column. All names were checked in IPNI.

**Species name**	**Frequency**	**Family**	**Full-scale use ***
Sedum album L.	58	Crassulaceae	50, 110, 204
Sedum acre L.	57	Crassulaceae	50, 204, 380
Sedum spurium M.Bieb.	51	Crassulaceae	13, 204, 253
Sedum rupestre L.	40	Crassulaceae	110, 253, 304
Sedum sexangulare L.	31	Crassulaceae	110, 204, 253
Sedum kamtschaticum Fisch.	29	Crassulaceae	110, 204
Sedum lineare Thunb.	22	Crassulaceae	196, 402, 420
Allium schoenoprasum L.	14	Amaryllidaceae	174
Festuca ovina L.	14	Poaceae	204, 205, 367
Festuca rubra L.	14	Poaceae	49, 141, 356
Achillea millefolium L.	13	Asteraceae	49, 70, 204
Sedum mexicanum Britton	13	Crassulaceae	50, 109
Dianthus carthusianorum L.	13	Caryophyllaceae	90, 163, 367
Trifolium arvense L.	12	Fabaceae	367
Trifolium repens L.	11	Fabaceae	50, 204, 326
Cynodon dactylon (L.) Pers.	10	Poaceae	196
Solidago bicolor L.	10	Asteraceae	141

Talinum calycinum Engelm. in Wisl.	9	Talinaceae	356
Poa pratensis L.	9	Poaceae	196, 422
Lavandula angustifolia Mill.	9	Lamiaceae	222, 389
Taraxacum campylodes G.E.Haglund	9	Asteraceae	-
Rosmarinus officinalis L.	9	Lamiaceae	-
Danthonia spicata (L.) Roem. & Schult.	9	Poaceae	-
Ophiopogon japonicus (Thunb.) Ker Gawl.	8	Asparagaceae	-
Erigeron canadensis L.	8	Asteraceae	-
Armeria maritima (Mill.) Willd.	8	Plumbaginaceae	49, 70, 356
Pilosella officinarum Vaill.	8	Asteraceae	-
Leucanthemum vulgare (Vaill.) Lam.	8	Asteraceae	-
Sedum hybridum L.	7	Crassulaceae	253
Sedum hispanicum L.	7	Crassulaceae	253

* = SM2 Reference number

Table S4.4. Selected species most frequently found in publications from tropical climate as being adequate for green roof use. For those species recognized as being of full-scale consolidated use, corresponding references in Supplementary Material 2 are indicated in the last column. All names were checked in IPNI.

**Species name**	**Frequency**	**Family**	**Full-scale use ***
Sedum lineare Thunb.	16	Crassulaceae	196, 402, 420
Portulaca grandiflora Hook.	9	Portulacaceae	50, 109
Sedum spurium M.Bieb.	7	Crassulaceae	13, 204, 253
Sedum rupestre L.	5	Crassulaceae	110, 253, 304
Sedum kamtschaticum Fisch.	5	Crassulaceae	110, 204
Sedum mexicanum Britton	5	Crassulaceae	50, 109
Talinum calycinum Engelm. in Wisl.	5	Talinaceae	356
Sansevieria trifasciata Prain	5	Asparagaceae	-
Lactuca sativa L.	5	Asteraceae	-
Phedimus aizoon (L.) ‘t Hart	5	Crassulaceae	-
Sphagneticola trilobata (L.) Pruski	5	Asteraceae	-
Axonopus compressus (Sw.) P.Beauv.	5	Poaceae	-
Arachis pintoi Krapov. & W.C.Greg.	5	Fabaceae	-
Zoysia matrella (L.) Merr.	4	Poaceae	359
Callisia repens (Jacq.) L.	3	Commelinaceae	-

Bulbine frutescens (L.) Willd.	3	Xanthorrhoeaceae	-
Callirhoe involucrata (Torr. & A.Gray) A.Gray	3	Malvaceae	-
Tradescatia spathacea Sw.	3	Commelinaceae	-
Duranta erecta L.	3	Verbenaceae	-
Scaevola tacada (Gaertn.) Roxb.	3	Goodeniaceae	-
Schefflera arborícola (Hayata) Merr.	3	Araliaceae	-
Ipomoa pes-caprae (L.) R. Br.	3	Convolvulaceae	-
Portulacaria afra Jacq.	3	Didiereaceae	-
Sedum acre L.	2	Crassulaceae	50, 204, 380
Sedum sexangulare L.	2	Crassulaceae	110, 204, 253
Allium schoenoprasum L.	2	Amaryllidaceae	174
Armeria maritima (Mill.) Willd.	2	Plumbaginaceae	49, 70, 356
Portulaca oleracea L.	2	Portulacaceae	-
Delosperma cooperi (Hook.f.) L.Bolus	2	Aizoaceae	-
Symphyotricum laeve (L.) Á.Löve & D.Löve	2	Asteraceae	-

* = SM2 Reference number
