# Supplementary material for: Life at the Top: Extensive Green Roof Plant Species and Their Traits for Urban Use
Source: Plants (Basel). 2025 Feb 27;14(5):735. doi: 10.3390/plants14050735 (PMC11902263; doi:10.3390/plants14050735)
Supplement: Supplementary file 1 [file plants-14-00735-s001.zip › S5 - Top families per climate pie charts - R1.pdf]

Supplementary Material S5

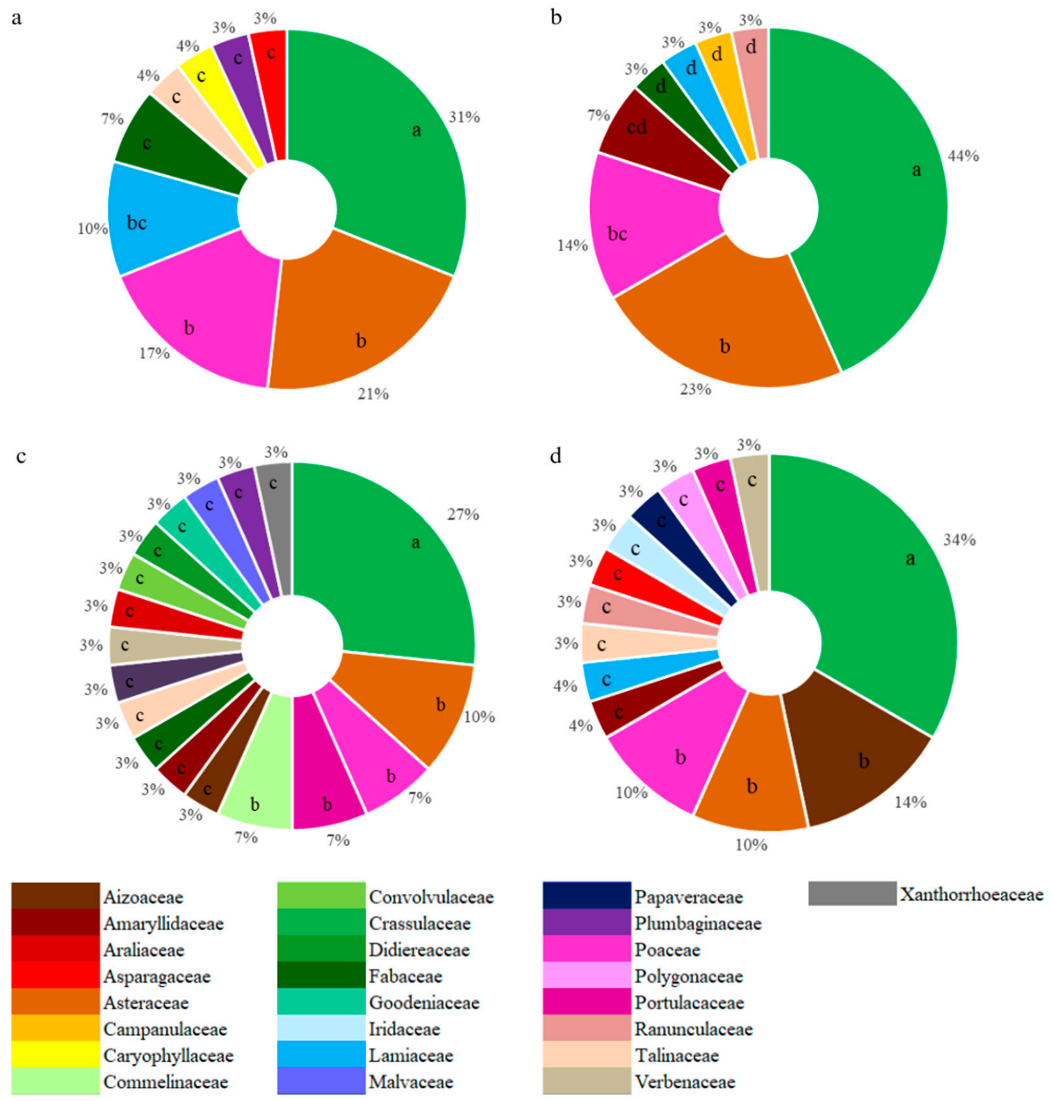

Top mentioned green roof botanical families in each climate: (a) temperate, (b) continental, (c) tropical, and (d) dry. Sections not sharing a letter within each climate are significantly different ($p \leq 0.05$).
